# Isolation, molecular identification, and genomic analysis of *Mangrovibacter phragmitis* strain ASIOC01 from activated sludge harboring the bioremediation prowess of glycerol and organic pollutants in high-salinity

**DOI:** 10.3389/fmicb.2024.1415723

**Published:** 2024-06-25

**Authors:** Hong Soon Chin, Narendrakumar Ravi Varadharajulu, Zhi-Han Lin, Wen-Yu Chen, Zong-Han Zhang, Sankar Arumugam, Ching-Yen Lai, Steve S.-F. Yu

**Affiliations:** ^1^Institute of Chemistry, Academia Sinica, Taipei, Taiwan; ^2^Chemical Biology and Molecular Biophysics Program, Taiwan International Graduate Program, Academia Sinica, Taipei, Taiwan; ^3^Institute of Bioinformatics and Structural Biology, National Tsing Hua University, Hsinchu, Taiwan; ^4^Molecular Science and Technology Program, Taiwan International Graduate Program, Academia Sinica, Taipei, Taiwan; ^5^Department of Chemistry, National Tsing Hua University, Hsinchu, Taiwan; ^6^Institute of Biochemical Sciences, National Taiwan University, Taipei, Taiwan; ^7^Ph.D. Program in Microbial Genomics, National Chung Hsing University, Taichung City, Taiwan

**Keywords:** *Mangrovibacter*, activated sludge, *M. phragmitis*, species delimitation, whole genome sequencing, glycerol degradation, bioremediation

## Abstract

The physiological and genotypic characteristics of *Mangrovibacter* (MGB) remain largely unexplored, including their distribution and abundance within ecosystems. *M. phragmitis* (MPH) ASIOC01 was successfully isolated from activated sludge (AS), which was pre-enriched by adding 1,3-dichloro-2-propanol and 3-chloro-1,2-propanediol as carbon sources. The new isolate, MPH ASIOC01, exhibited resilience in a medium containing sodium chloride concentration up to 11% (with optimal growth observed at 3%) and effectively utilizing glycerol as their sole carbon source. However, species delimitation of MGBs remains challenging due to high 16S rRNA sequence similarity (greater than 99% ANI) among different MGBs. In contrast, among the housekeeping gene discrepancies, the tryptophan synthase beta chain gene can serve as a robust marker for fast species delimitation among MGBs. Furthermore, the complete genome of MPH ASIOC01 was fully sequenced and circlized as a single contig using the PacBio HiFi sequencing method. Comparative genomics revealed genes potentially associated with various phenotypic features of MGBs, such as nitrogen-fixing, phosphate-solubilizing, cellulose-digesting, Cr-reducing, and salt tolerance. Computational analysis suggested that MPH ASIOC01 may have undergone horizontal gene transfer events, possibly contributing unique traits such as antibiotic resistance. Finally, our findings also disclosed that the introduction of MPH ASIOC01 into AS can assist in the remediation of wastewater chemical oxygen demand, which was evaluated using gas chromatograph-mass spectrometry. To the best of our knowledge, this study offers the most comprehensive understanding of the phenotypic and genotypic features of MGBs to date.

## 1 Introduction

Though the genus *Mangrovibater* (MGB) had recently been discovered (Rameshkumar et al., 2010), very little was understood about it. Multiple reports suggested that MGBs are abundant and widely distributed across diverse habitats, highlighting the importance of investigating their role in the ecosystem. At present, there are only three published valid species of MGB. The MGB genus within the Enterobacteriaceae family comprises *M. phragmitis* (MPH), *M. yixingensis* (MYI), and *M. plantisponsor* (MPL). MPH MP23^T^, MYI TULL-A^T^, and MPL MSSRF40^T^ were isolated from the roots of *Phragmites karka* (Behera et al., 2017), farmland soil (Zhang et al., 2015), and mangrove-associated wild rice plants (Rameshkumar et al., 2010), respectively. These strains are facultatively anaerobic and gram-negative, showing plant growth-promoting and nitrogen-fixing properties.

The primary sources of MGB pure cultures were predominantly obtained from the regions of East Asia, South East Asia, and South Asia and extensively identified in various habitats (Table 1, Supplementary Tables S1A, S1B) using 16S rRNA-based amplicon sequencing analysis. The distribution of MGBs includes high-salt sludges (Li et al., 2019, 2020; Yin et al., 2022), tannery effluent (Lian et al., 2016a,b; Sanjay et al., 2020), brackish water (Kiun et al., 2016), mangrove rhizosphere sediment (BioProject no PRJNA682484), farm soil (Zhang et al., 2015), shrimp aquaculture farm (Joseph et al., 2014), storage tanks containing biodiesel (White et al., 2011), water droplets from within the bitumen (Himmelberg, 2019), geothermal lake (MYI strain CEMTC_1,393, GenBank accession no. OP143686.1), and pond (“Shoubuike”) (Fujita et al., 2023).

**Table 1:** The occurrence of MGBs in natural habitat and its fundamental attributes.

Groups	Source	Species/Genus	Abundancy	Experimental procedures	Isolation media	Reference/GenBank	Remarks
Free-living	Salt-tolerant sludge	MPH ASIOC01	<1%	V3-V4 and FL metagenomic	HDB medium with 1,3-DCP & 3-MCPD	This study	Genome sequencing completed (unpublished data)
	Aerobic granular sludge (AGS)	MGB	Up to 35.9%	V3–V4 metagenomics	NIA	Li et al. (2019)	With addition of acyl-homoserine lactone (AHL)
	Salt-tolerant AGS	MGB	Up to 74.9%	V3–V4 metagenomics	NIA	Li et al. (2020)	High abundancy of MGB as initial stage
	Salt-tolerant sludge	MGB	Up to 45.7%	Universal 16S rRNA primers metagenomics	NIA	Yin et al. (2022)	Acclimated sludge containing 70 g/L NaCl
	Tannery effluent sludge	MPL CR1	NIA	Isolation & 16S rRNA sequencing	LB with 0.16 mM AQS & 1 mM Cr (VI)	Lian et al. (2016b)	Cr (VI) reduction
	Tannery effluent (semi solid soil)	MYI MS 2.4	NIA	Isolation & 16S rRNA sequencing	Nutrient agar	Sanjay et al. (2020)	Cr (VI) reduction
	Textile effluent	MYI AKS2	NIA	NIA	NIA	Accession no.: OM189530.1/ Nasrin et al. (2022)	Bioremediation of BR-18 dye
	Blackish water	MPL UMTKB3	NIA	Isolation & 16S rRNA sequencing	Nutrient rich (NR) media	Kiun et al. (2016)	MPL produced PHA when grew in medium using glucose as carbon source and PHA production were confirmed with GC
	Soil sediment from brackish water	MGB sp. AB6	NIA	NIA	NIA	Accession no.: JX188076.1	NIA
	Mangrove rhizosphere sediment	MYI SaN21-3	NIA	Isolation & WGS	Bacto marine broth	BioProjects: PRJNA682484	Draft genome available
	Farmland soil	MYI TULL-A^T^	NIA	Isolation & species determination	LB agar	Zhang et al. (2015)	Type strain
	Copper containing wastewater/ sewage	MGB sp. A1/MGB sp. WG-1	NIA	NIA	NIA	Accession no.: MT762841.1/FJ404760.1	NIA
	AS from oil field wastewater-treating system	*Salmonella*/MGB sp. ZZ-4	NIA	NIA	NIA	Accession no.: FJ667502.1	NIA
	Shrimp aquaculture farm	MGB sp. MFB070	NIA	Isolation and WGS	NIA	Joseph et al. (2014)	Draft genome available
	Automotive biodiesel	Enterobacteriaceae bacterium JW72.7a	5%	DGGE, 16S rRNA gene V6 amplicon pyrosequencing & strain genotyping	Tryptic soy agar	White et al. (2011)/Accession no.: FN556578.1	Contamination bacteria in biodiesel tank
	Geothermal lake	MYI CEMTC 1393	NIA	Isolation & 16S rRNA sequencing	NIA	Accession no.: OP143686.1	NIA
	Pond (“Shoubuike”)	MGB	NIA	Shotgun metagenomic sequencing	NIA	Fujita et al. (2023)	NIA
	Water drop of natural oil emitting lake	MGB	NIA	NIA	NIA	Himmelberg (2019)	Single Cell Sequencing
Endophytes	Roots of *Phragmites karka*	MPH MP23^T^	NIA	Isolation & species determination	LB agar	Behera et al. (2017)	Type strain and draft genome available
	*Spartina alterniflora* (salt-water cordgrass)	Endophytic bacterium SV706, SV708, SV717, SV812	0.17-Oiled 0.33-Unoiled	Morphospecies Sanger sequencing	Nutrient agar	Kandalepas et al. (2015)/ Accession nos.:. KP757589.1, KP757590.1, KP757597.1 & KP757662.1	Root endophyte, 4.68% contributions
	Mangrove-associated wild rice	MPL MSSRF40^T^	NIA	Isolation & species determination	NfM + Y agar	Rameshkumar et al. (2010)	Type strain and draft genome available
	Mangrove rhizosphere/root	MPL MSSRF N80 and MPL BCRP5	NIA	NIA	NIA	Accession nos.: KU131265.1 & MT422011.1	MGB sp. MSSRF N44, MSSRF N87, MSSRF N97, MSSRF N107, P4, P5, P6, P7, P12, P13, P16, P21, *Klebsiella/*MGB sp. P23 (Accession nos listed in Supplementary Table S1B)
	Todos os Santos Bay oil-contaminated mangrove	MGB sp. QUEBA02 & 03	NIA	NIA	NIA	Accession nos.: JQ658399.1 & JQ658400.1	A potential hydrocarbon-degrading bacterium isolated from Brazilian mangrove sediment
	*Bruguiera sexagula* and *Ceriops decandra*	NVVC2a and BDMO2b	NIA	Isolation & 16S rRNA sequencing	Burk’N free medium or NBRIP medium	Tam et al. (2019)	Media supplied with 2% NaCl, most likely belongs to MPL
	Rhizosphere soil	MGB	NIA	V3–V4 metagenomics	NIA	Chaluvadi and Bennetzen (2018)	Root endophytic microbes
	*Ginkgo Biloba* Leaves	MGB	>1%	V4 region (799F & 1115R) metagenomics	NIA	Lei et al. (2021)	NIA
	*Euterpe oleracea* fruits	MGB	<1%	V4 region (341F & 805R) metagenomics	NIA	Moura et al. (2018)	Detected during the spontaneous decay process
	Pulped natural fermented coffees	MGB	NIA	V3–V4 metagenomics	NIA	Martinez et al. (2022)	Distinctive species at altitude of 800 m
	*Garcinia mangostana* pericarp	MPL 21A1 & 21A3	NIA	NIA	Plate count agar	So’aib et al. (2019)	Detected at fermentation day 20–30
Zoonotic	Gut of *Zophobas atratus larvae*	MGB	Up to 21.88%	V3–V4 metagenomics	NIA	Luo et al. (2021)	PU-fed superworms
	Gut of *Zophobas atratus larvae*	MGB	Up to 21.28%	V3–V4 metagenomics	NIA	Wang et al. (2022)	PU-fed superworms
	Cecum of *Gallus gallus domesticus*	MGB	0.01–0.4%	Set 1 (V2–4–8) and Set 2 (V3–6, 7–9)	NIA	Neveling et al. (2017)	NIA
	Intestine of juvenile *Micropterus salmoides*	MGB	Up to 5.88%	V4–V5 metagenomics	NIA	Zhao et al. (2022)	Fed diets with methionine hydroxy analogue
	Intestine of shrimp	MGB	4.99%	V4 region (15F and 806R) metagenomics	NIA	Sun and Xu (2021)	NIA
	*Bursaphelenchus xylophilus* (pine wood nematode)	MGB sp. Arv-29-1.1a	1.7% (% frequency from *P. nigra*)	NIA	Reasoner’s 2A agar	Proença et al. (2014)/Accession no.: KF214973.1	Frequencies of bacteria taxa carried by *B. xylophilus* isolated from *Pinus nigra*
	*Euryphorus nordmannii*	MYI JvC08	NIA	RAPD typing and sequencing	NIA	Accession no.: OL347579.1	Present in parasitic copepods and isopods
	Hindgut of *Neosarmatium indicum*	MGB sp. Nin4-HG	NIA	Isolation & 16S rRNA sequencing	NIA	Accession nos.: OP393728.1 & OP393723.1	Potential contribution to cellulose digestion in host
	Midgut or hindgut of *Episesarma versicolor*	MGB sp. Eve2-HG	NIA	Isolation & 16S rRNA sequencing	NIA	Accession nos.: OP393571.1 to OP393580.1	Potential contribution to cellulose digestion in host (Complete list was tabulated in Supplementary Table S1B)

NIA, No information available.

MGBs have been observed to exhibit endophytic characteristics, wherein they establish a symbiotic relationship with plants. MGBs have been documented to occur within the root systems or foliage of various plant species, such as *Bruguiera sexagula*, *Ceriops decandra*, *Porteresia coarctata* Tateoka, *Phragmites karka*, *Setaria viridis, Ginkgo biloba,* and *Spartina alterniflora* (Rameshkumar et al., 2010; Kandalepas et al., 2015; Behera et al., 2017; Chaluvadi and Bennetzen, 2018; Tam et al., 2019; Lei et al., 2021). Additionally, they have been found in the fruits of *Euterpe oleracea* (Moura et al., 2018) and plant-derived fermented products such as coffee beans (Martinez et al., 2022) and *Garcinia mangostana* pericarps (So’aib et al., 2019). Endophytic microorganisms play a crucial role in facilitating the growth of plants through their ability to enhance nutrient absorption, bolster stress tolerance, and fortify resistance against diseases. These beneficial effects ultimately contribute to the enhancement of crop yields (Watts et al., 2023).

MGBs have also been identified as symbiotic bacteria in several organisms throughout the animal kingdom, including the gut of superworm (Luo et al., 2021; Wang et al., 2022), cecum of *Gallus gallus domesticus* (Neveling et al., 2017), intestine of largemouth bass *Micropterus salmoides* (Zhao et al., 2022), intestine of shrimp (Sun and Xu, 2021), hindgut of *Neosarmatium indicum* (GenBank accession numbers (nos.) OP393728.1 & OP393723.1), and midgut or hindgut of *Episesarma versicolor* (GenBank accession nos. OP393571.1 to OP393580.1). Additionally, there have been reports indicating the potential for symbiotic growth between MGB and *Euryphorus nordmannii*, which are parasitic copepods and isopods (GenBank accession no OL347579.1), as well as *Bursaphelenchus xylophilus*, often known as the pinewood nematode (Proença et al., 2014). It is noteworthy that the gastrointestinal tract of superworms fed with polyurethane (PU) presented a significant predominance of MGBs, accounting for a remarkable 21% of the overall gut microbiota (Luo et al., 2021; Wang et al., 2022). A somewhat moderate prevalence of MGBs, accounting for approximately 6% of the total microbiota, was observed in the intestinal tract of largemouth bass which was provided with a diet supplemented with methionine hydroxy analog (Zhao et al., 2022). Similarly, in the case of shrimp, MGBs constituted approximately 5% of its total microbiota in the intestine (Sun and Xu, 2021). A significant prevalence of MGBs was observed in high-salt sludge (Yin et al., 2022), aerobic granular sludge (AGS) with the introduction of acyl-homoserine lactone (Li et al., 2019), and at the early phase of halotolerant aerobic granulation for AGS formation (Li et al., 2020). The MGBs account for 45.7, 35.9, and 74.9% of the overall microbiome abundance observed in sludges as mentioned earlier (Table 1). The present observation of a notably elevated prevalence of MGBs, particularly during the initial phases of AGS development, suggests that MGBs could potentially fulfill the role of primary colonizers (Lee et al., 2023a). MGBs might play a vital part in supporting the formation and modification of biofilms and modulating surface characteristics, hence facilitating the colonization of diverse species inside activated sludge (AS) systems.

Currently, the ecological role of MGBs remains to be determined. The sole unequivocal biochemical characteristic that has been documented is its capacity to diminish the carcinogenic chromium ions [Cr(VI)] (Lian et al., 2016b; Sanjay et al., 2020). Previous studies have suggested that MGBs have the ability to effectively reduce basic Red-18 dye (Nasrin et al., 2022), polyurethane (Luo et al., 2021; Wang et al., 2022), and even bitumen (Himmelberg, 2019). Lately, it was suggested that when the microbial community is at the simplest network structure (originally sampled from a pond), MGB provides arginine and ethanol to other bacteria such as *Hydrotalea*, *Terracidiphilus*, and *Rhizomicrobium* (Fujita et al., 2023). However, it is imperative to perform additional analysis on these observations in order to fully understand their bioremediation capabilities and ecological significance in the microbiome.

The biodiesel industry has experienced significant growth as a result of many initiatives undertaken by the transportation sector to achieve carbon-neutral mobility. However, the additional production of glycerol as a waste causes a significant drop in its market value due to surplus availability (Chilakamarry et al., 2022; Agrawal et al., 2023). As a result, glycerol-utilizing microbes enable the production of beneficial chemical intermediates and valuable products in a sustainable manner. Similarly, it has been noted that MGBs had the capacity to efficiently utilize glycerol as a way of biomass accumulation. This finding indicates the potential for repurposing glycerol with the use of MGBs (Chilakamarry et al., 2022; Moklis et al., 2023).

Several experiments employed nutrient-rich media to facilitate the growth of MGBs, including bacto marine broth, LB broth supplemented with 15% (v/v) glycerol (Zhang et al., 2015), LB agar (Behera et al., 2017), brain heart infusion agar (Sanjay et al., 2020), and a medium consisting of meat extract, yeast extract, and peptone (Kiun et al., 2016). These nutrient-rich media are not recommended due to their inability to eradicate undesirable microbial flora effectively. The second strategy for strain separation was based on the distinctive physiological and biochemical characteristics of MGBs. MGBs are nitrogen-fixing bacteria that are capable of independent growth and thrive in a nitrogen-depleted environment. Consequently, they can be isolated using Burk’N free medium supplemented with 2% NaCl (Tam et al., 2019) or an N-free medium with the inclusion of yeast extract (NfM + Y agar) (Rameshkumar et al., 2010). MGBs can also be obtained through isolation techniques utilizing the phosphate growth (NBRIP) medium developed by the National Botanical Research Institute, which is supplemented with a 2% concentration of NaCl (Nautiyal, 1999; Tam et al., 2019). The NBRIP medium was initially formulated for the purpose of screening bacteria that possess the ability to solubilize phosphate. Due to the ability of MGBs to effectively decrease Cr(VI) levels, it is feasible to cultivate and isolate MGBs on LB agar plates that have been supplemented with Cr(VI) (Lian et al., 2016b; Sanjay et al., 2020). All documented isolation strategies for MGB have been compiled and presented in Table 1.

In this study, we opted to use the cutting-edge PacBio HiFi method to sequence the genome of MPH ASIOC01, which was isolated from high-salt AS. The objective of this study is to investigate potential enrichment methods and assess various species delimitation strategies. The primary goal is to enhance the isolation of novel MGBs with greater efficacy and examine the underlying phenotypic and biochemical characteristics of these MGBs. These findings are anticipated to offer valuable insights into the identification of new MGB species and aid in enhancing our understanding of their ecological impact across various habitats.

## 2 Materials and methods

### 2.1 Preparation of AS samples for metagenomic analysis

AS and wastewater samples from a large-scale membrane bioreactor (MBR) were supplied periodically by the wastewater treatment facility of a local petrochemical refinery plant. The AS and wastewater samples contain residual amount of epichlorohydrin (ECH) and acrylonitrile (AN). The AS was pre-enriched with 1,3-dichloro-2-propanol (1,3-DCP) and 3-chloro-1,2-propanediol (3-MCPD) at 30°C with 195 rpm shaking for 48 h. The genomic DNA (gDNA) was extracted using Presto™ Soil DNA Extraction Kit (Geneaid Biotech, Taiwan).

### 2.2 Isolation and characterization of MPH

In total, 0.5 g of fresh AS was added to 45 mL hydrocarbon-degrading bacteria (HDB) medium (0.5 g/L NH_4_Cl, 0.5 g/L MgSO_4_·7H_2_O, 0.4 g/L NaCl, 0.05 g/L KH_2_PO_4_, 0.05 g/L Na_2_HPO_4_·H_2_O and trace elements). Overall, 25 μL of 1,3-dichloro-2-propanol (1,3-DCP, cat. No. A16003, Alfa Aeser, United States) and 3-chloro-1,2-propanediol (3-MCPD, Cat no: 107271, Aldrich, United States) were added as enrichment carbon source. The mixture was then incubated at 30°C with 195 rpm agitation for 72 h. Subsequently, 50 μL of the 3-day-old culture was sub-cultured into 50 mL of fresh HDB medium containing the same quantity of carbon source and incubated for another 72 h. We selected a single colony from the second HDB medium culture using HDB agar plate and repeated the single colony selection procedure until pure culture was obtained. Pure culture was maintained on Luria–Bertani (LB) agar, and glycerol stock of the cultures was maintained at −80°C. MPH ASIOC01 was propagated in LB agar supplied with NaCl to determine its maximal salt tolerance (8–12% NaCl). MPH ASIOC01 was also grown in modified mineral salt medium (MMSM) with 1% (v/v) glycerol as a sole carbon source at a wide range of salt concentrations (1.5–6% NaCl). MMSM contains 2.0 g/L (NH_4_)_2_HPO_4_, 0.2 g/L NH_4_H_2_PO_4_, 1.0 g/L KNO_3_, 1.0 g/L MgSO_4_, 0.2 g/L CaCl_2_, and 0.25 g/L yeast extract and trace element with final pH adjusted to 7.5. Finally, the modified M9 (MM9) medium, supplied separately with glycerol, glucose, fructose, ethanol, or acetate (final concentration at 1% v/v) as sole carbon sources, was additionally applied for the culture of MPH ASIOC01. MM9 contains 6.78 g/L Na_2_HPO_4_, 3.0 g/L KH_2_PO_4_, 1.0 g/L NH_4_Cl, 0.25 g/L Na_2_SO_4_, 1.0 g/L, MgSO_4_, 0.2 g/L CaCl_2_, and 0.25 g/L yeast extract and trace element with final pH adjusted to 7.5. Overall, 1,000X trace element stock solution contains 50 mM FeCl_3_, 20 mM CaCl_2_, 10 mM MnCl_2_, 10 mM ZnSO_4_, 2 mM CoCl_2_, 2 mM CuCl_2_, and 2 mM NiCl_2_.

### 2.3 Identification, isolation, and characterization of polyphosphate (polyP) accumulated in the MPH ASIOC01

The bacterial solutions were grown in a 5% NaCl MM9 medium, to detect polyphosphate (polyP) accumulated in the MPH cells, which were then analyzed using a laser scanning microscope (Carl Zeiss LSM 780) equipped with Plan-Apochromat 100× oil objectives with 1.40 numerical aperture (NA). DAPI-DNA complexes were excited at the wavelength of 405 nm, and the filters were set at 410 nm, whereas DAPI-PolyP was excited at the wavelength of 405 nm and the filters were set at 694 nm. The acquired Images were further analyzed with Zen blue software version 3.5 (Carl Zeiss Microscopy, Deutschland GmbH).

After incubation at 30°C for 72 h, 20 mL of MPH ASIOC01 cultured in MM9 containing 5% NaCl was centrifuged at 8,000 rpm for 10 min. The cell pellet was then re-suspended in 25 mL of NaOH/EDTA mixture (1:1 mix of 0.5 M NaOH prepared with autoclaved milliQ water and 0.1 M autoclaved Na_2_EDTA). The samples were extracted overnight (18 h) at an incubator 30°C with 200 rpm of shaking and then centrifuged at 10,000 rpm for 25 min. Before ^31^P-NMR analysis, an aliquot of polyP sample solution was mixed with 10% D_2_O. The chemical shifts were determined relative to an external alkaline standard of 0.05 M KH_2_PO_4_ in a NaOH/EDTA solution with 10% D_2_O. Pyrophosphate (Cat. No. P8010, Aldrich, United States) and sodium hexametaphosphate (SHMP, cat. No. 71600, Aldrich, United States), at final concentrations of 2 mM and 0.2 g/L, respectively, were served as the external standards. Resonance peaks for different phosphorus species were assigned at the sample pH condition, according to the literature (Sannigrahi and Ingall, 2005). Overall, 162 MHz ^31^P-NMR spectra were acquired by a Bruker Avance NMR spectrometer (400 MHz ^1^H-NMR frequency). The procedure for the polyP NMR study was modified according to the reported procedures (Wang et al., 2021).

The scanning electron micrographs of MPH were performed according to the following procedure. In brief, the bacterial sample was fixed in 0.1 M sodium phosphate buffer (pH 7.0) containing 2.5% glutaraldehyde and 4% formaldehyde at room temperature for 1 h, rinsed and post-fixed in 1% OsO_4_ in the same buffer for another 1 h, and then rinsed again and dehydrated in an ethanol solution. Critical point drying was performed with a Leica EM CPD 300 critical point dryer. Sample coatings were carried out with Hitachi E-1010 ion sputter. Finally, the processed samples were visualized with field-emission scanning electron microscopy (FE-SEM, Zeiss Group, Model ULTRA PLUS).

### 2.4 Design of oligonucleotide primers specific for the genus and species of MGBs

The identity of pure culture was then determined via 16S rRNA gene sequencing. Overall, 27F forward primer (5’-AGAGTTTGATCCTGGCTCAG-3′) and 1492R reverse primer (5’-GGTTACCTTGTTACGACTT-3′) were used for the amplification of 16S rDNA (Innis et al., 1990; Lane, 1991). Universal forward primer 5’-CGTATTTTGGTGAATTCGG-3′ and reverse primer of 5’-CGTGAACCGTGAAAATG-3′ were designed for the amplification of MGB tryptophan synthase beta chain genes (*trpB*). A single colony was picked and directly dip into the PCR mix (Taq DNA Polymerase Master Mix RED, Ampliqon, Denmark) containing the corresponding primer set without the need of gDNA extraction. Polymerase chain reaction (PCR) was performed using a Biometra T3000 Thermocycler (Biometra, Germany). Overall, 16S rDNA and *trpB* DNA fragments were excised for agarose gel and subjected to Sanger sequencing, which was conducted by Genomics Inc., Taiwan using Applied Biosystems 3730xl DNA Analyzer (Applied Biosystems, United States). The output chromatograms were analyzed and evaluated using SnapGene Viewer version 5.3 (GSL Biotech, United States).

### 2.5 Genomic analysis, comparison, and visualization

Evaluation of genome assemblies was performed using the Quality Assessment Tool for Genome Assemblies (QUAST) (Gurevich et al., 2013). The nucleotide and whole genome sequencing (WGS) data were then analyzed using QIAGEN CLC Genomics Workbench,^1^ Biocyc Pathway Tools v27 (Karp et al., 2019), and PATRIC v3.6.12^2^ (Davis et al., 2020). BLAST (basic local alignment search tool) search was performed using NCBI services and databases (Altschul et al., 1990). Classification of protein family (pfam) search was performed via InterPro server (Paysan-Lafosse et al., 2023). Multi-locus species tree was constructed using the on-line Automated Multi-Locus Species Tree (autoMLST) program (Alanjary et al., 2019). Ribosomal Multilocus Sequence Typing (rMLST) was performed using PubMLST^3^ (Jolley et al., 2012). Multiple sequence alignment, calculation of percent identity matrix, and phylogeny analysis were performed using MEGA-11 software (Tamura et al., 2021) and Clustal Omega v1.2.4 (Sievers et al., 2011).^4^ Poisson tree processes (PTP) for single-locus species delimitation were performed as described (Kapli et al., 2017). The display, annotation, and management of the phylogenetic tree were performed using iTOL v6^5^ (Letunic and Bork, 2021) and Reference Sequence Alignment-based Phylogeny Builder (REALPHY v1.13) (Bertels et al., 2014). Genomic comparison and visualization were carried out using both the BLAST Ring Image Generator (BRIG) and Proksee. BRIG was utilized to construct circular comparison maps of the genomes with the genome of MPH ASIOC01 as a reference. The other 4 MGB genomes were compared against this reference to identify the conserved regions (Alikhan et al., 2011). Additionally, the web-based genome map visualization tool Proksee was employed for an enhanced genome comparison (Grant et al., 2023). Proksee facilitated the addition of an extra ring to the genome map using the Alien Hunter plugin (Vernikos and Parkhill, 2006) to predict potential horizontal gene transfer (HGT) events by identifying regions of atypical sequence composition.

### 2.6 Gene annotation analysis

The genomes and the corresponding coding sequences (CDSs) of MPH MP23^T^, MYI SaN21-3, MPL DSM19579, MGB sp. MFB070, and *Salmonella enterica* str. LT2 were retrieved from NCBI Reference Sequence Database and presented in Supplementary Tables S1A, S1B. COGclassifier v1.0.5 was used to categorize the CDS of each genome into Clusters of Orthologous Groups (COGs) (Tatusov et al., 2000; Shimoyama, 2022). Gene Ontology (GO) terms were annotated via Interproscan-5.63-95.0 (Harris et al., 2004; Jones et al., 2014). The gene assignments from Kyoto Encyclopedia of Genes and Genomes (KEGG) Orthology (KO) and the resulting KEGG pathway analysis were performed by BlastKOALA version 3.0 and KEGG Mapper through the KEGG web service (Kanehisa et al., 2016a,b). CAZyme genes were identified in the genomes using dbCAN3 server (Zheng et al., 2023). The biosynthetic gene clusters (BGC) were identified by antiSMASH bacterial version 7.0.0 (Blin et al., 2023).

### 2.7 Phylogenetic tree construction, functional annotation, and distribution analysis at pan-genome level

The pan-genome matrix, phylogenetic tree, principal component analysis (PCA) plot, and core-accessory gene pie chart were generated using Roary v3.7.0 (Page et al., 2015). We reannotated the downloaded DNA fasta files (Supplementary Table S1A) using Prokka v1.13 (Seemann, 2014), to ensure consistency in the subsequent analyses. The gff files generated by Prokka were used as input for Roary. The pan-genome matrix was constructed with a 90% BLASTP identity cutoff. The phylogenetic tree was constructed using FastTree (Price et al., 2010) integrated within Roary. The KEGG and COG distribution analyses were performed using the Bacterial Pan Genome Analysis tool (BPGA v1.3) (Chaudhari et al., 2016). The annotated protein sequences from Prokka were used as input for BPGA. The tool was run with USEARCH (Edgar, 2010), a user-defined clustering process for clustering at 80% sequence identity.

### 2.8 Nucleotide sequence and accession number

In this study, the list of MGBs together with its genome assembly numbers and GenBank accession numbers (nos.) are shown in Supplementary Tables S1A, S1B. The GenBank BioSampleID for MPH strain ASIOC01 is SAMN37132003, which is deposited on 24 August 2023.

### 2.9 Chemical oxygen demand (COD) and chemical pollutant reduction analysis

For COD reduction analysis, a wastewater solution (50 mL) in a 250-mL Erlenmeyer flask containing 75% ECH wastewater and 2.5% AN wastewater and top-up with inorganic wastewater was incubated with MBR-high salt AS (approximately 3% NaCl), MPH strain ASIOC01, *Vibrio proteolyticus* strain B610AS (in-house isolated strain from AN AS), and *Acinetobacter ventiatus* RAG-1 (ATCC 31012/BCRC 14357) for 48 h in a 30°C incubator (Firstek Inc. Taiwan) with 195 rpm of shaking. The samples were then subjected to quantitation using COD test kit following the manufacturer’s recommendations (HACH, United States).

The reduction in chemical pollutants for the 2-day bioremediation procedure was analyzed using Agilent 7890B GC/5977B MSD equipped with DB-1MS Ultra Inert GC column (60 m × 0.25 mm × 0.25 μm) (Agilent, United States). Trifluorotoluene (200 ppm) was added to the remediated solution as an external standard. The bacterial and sludge-treated samples were also derivatized with BSTFA:TMCS (99:1) (TCI, Japan) at 55°C for 30 min prior to GC–MS analysis (Cavalheiro et al., 2014).

### 2.10 Statistical analysis

Statistical analyses and plot construction were performed using OriginPro v2021 (OriginLab Corporation, United States). *p* < 0.05 was considered statistically significant.

## 3 Results and discussion

### 3.1 Isolation of MGB from AS

MGBs comprised roughly 0.002% of the total microbiota found in the AS collected from the large-scale MBR facility at the ECH and AN manufacturing plant. The distribution ratio of MPH, MYI, and MPL within MBR sludges was found to be approximately 77:22:1, as shown in Supplementary Figure S1, utilizing V3-V4 short-read metagenomics technology (see Supplementary Document S1). Multiple bacterial strains were isolated from MBR sludges, utilizing HDB medium supplemented with 1,3-DCP and 3-MCPD as carbon sources for enrichment. These chemicals are present in significant quantities within the collected MBR-ECH wastewater. By harnessing its capacity to effectively utilize these distinct carbon sources, strains E301, E304, and E311 were isolated, purified, and later identified as MGB through the application of 16S rRNA PCR and Sanger sequencing techniques. The MGB isolates have the capability to undergo at least five passages and be sustained on LB agar or nutritional agar supplemented with 6% NaCl concentration.

**Figure 1:**
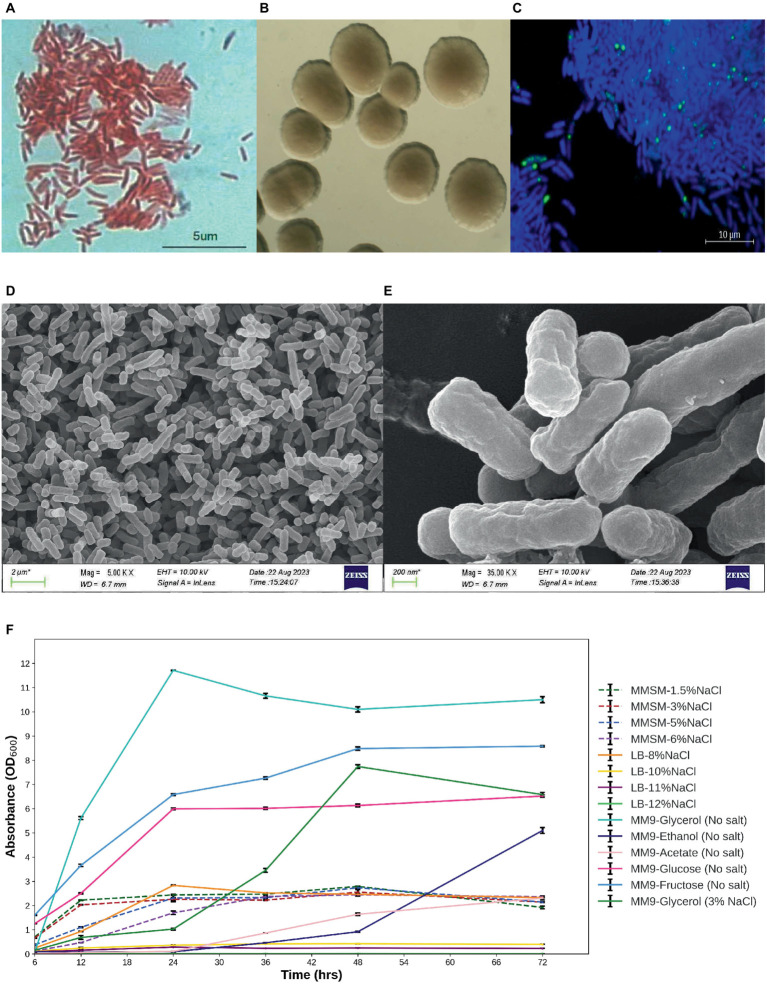
**(A)** Gram staining. **(B)** Colony morphology. **(C)** PolyP accumulation of MPH isolate E311 (renamed as MPH ASIOC01 after WGS) was determined using confocal laser scanning microscopy. **(D)** Field emission scanning electron micrograph (SEM) of MPH ASIOC01 at 5,000× magnification with scale bar of 2 μm. **(E)** Field emission SEM of MPH ASIOC01 at 35,000 × magnification with scale bar of 200 nm. **(F)** Growth curves of MPH isolate E311 in modified minimal salt medium (MMSM) with varying salt concentrations (1.5–6% NaCl) and 1% glycerol as the carbon source in salt-free modified M9 medium (MM9) with various carbon sources except for one containing glycerol and 3% NaCl and in LB medium with varied salt concentrations (8–12% NaCl).

The microbes under investigation exhibit characteristics of gram-negative bacteria and possess a rod-shaped morphology (Figure 1A). Following a 48-h incubation period at 30°C, these isolates displayed circular colonies with a creamy white appearance and smooth texture. The diameter of these colonies ranged from 1 to 2 mm on LB agar medium (Figure 1B). The high-resolution scanning electron micrographs of isolate E311 (MPH ASIOC01) are shown in Figures 1D,E. The strain can also grow in a modified minimal salt medium (MMSM) supplemented with 6% NaCl and in an LB medium supplemented with NaCl concentrations of up to 8%. MPH ASIOC01 can also grow effectively in a broad-spectrum carbon nutrient in MM9 using 1% (v/v) glycerol, glucose, fructose, ethanol, or acetate as the sole carbon sources without the addition of yeast extract (Figure 1F). In any case, the strain MPH ASIOC01 demonstrates effective utilization of glycerol on a salt-free MM9 medium, resulting in an optical density (OD_600_) of 12 after 24 h of incubation.

Accumulation of polyP in MPH ASIOC01 grown in an MM9 medium containing 5% NaCl is shown in Figure 1C. DAPI-stained polyP granules present in MPH show a bright yellow-green fluorescence with an excitation at 405 nm. The presence of polyP in MPH was further confirmed with distinct resonances at δ = −21.59 ppm for the ^31^P NMR analysis (Supplementary Figure S2) (Sannigrahi and Ingall, 2005). Utilizing confocal microscopy and ^31^P NMR investigation, MPH ASIOC01 can be recognized as a phosphate-accumulating organism (PAO).

### 3.2 Determination of MGB via 16S rDNA and housekeeping gene sequencing

#### 3.2.1 Determination of MGB via 16S rRNA sequencing

**Figure 2:**
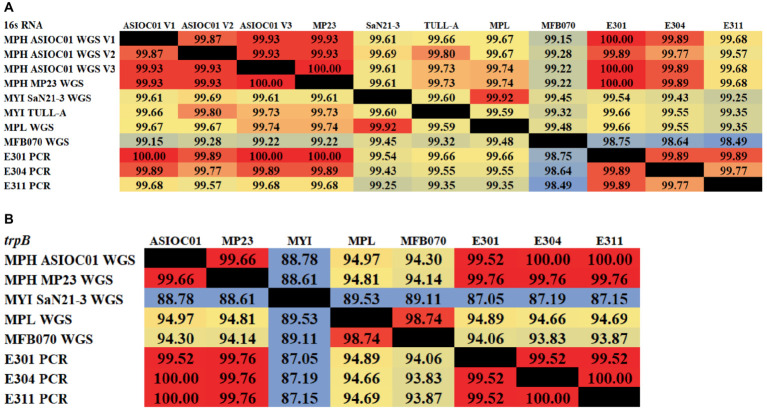
The average nucleotide identity (ANI) similarity index was determined for **(A)** the 16S rDNA and **(B)** *trpB* housekeeping gene among the MGBs with disclosed genome and the MPH isolated colonies of E301, E304, and E311. The analysis was performed using Clustal Omega v1.2.4. The sequences exhibiting the highest and lowest ANI similarity were visually distinguished by the use of dark red and pale blue colors, respectively.

rRNA sequencing has commonly been utilized for the identification of bacterial species. Bacterial taxonomists have proposed criteria of 97 and 98.65% similarity in the 16S rDNA sequence to distinguish between two bacterial species (Stackebrandt and Goebel, 1994; Kim et al., 2014). Nevertheless, the effectiveness of 16S rDNA analysis is intrinsically constrained due to the high prevalence of conserved regions within the 16S rDNA sequence (Ferraz Helene et al., 2022). The 16S rDNA sequences of MGBs exhibit a high degree of similarity. For example, the complete 16S rDNA sequence of MPH MP23^T^ displayed an ANI similarity of 99.61, 99.74, and 99.22% when compared with MYI SaN21-3, MPL MSSRF40^T^, and MGB sp. MFB070, respectively (Figure 2A). The task of accurately identifying MGB at the species level solely depends on rRNA Sanger sequencing approach and is challenging due to the high ANI similarity and poor taxonomic resolution. In accordance with the proposal put forth by Lin et al. (2022), it was argued that the utilization of 16S rRNA gene sequencing should not be regarded as the definitive method for the precise classification of *Elizabethkingia* species. This assertion is based on the observation that the divergence among the various copies of the 16S rDNA was consistently below 1% in all strains of *Elizabethkingia* (Lin et al., 2022).

**Figure 3:**
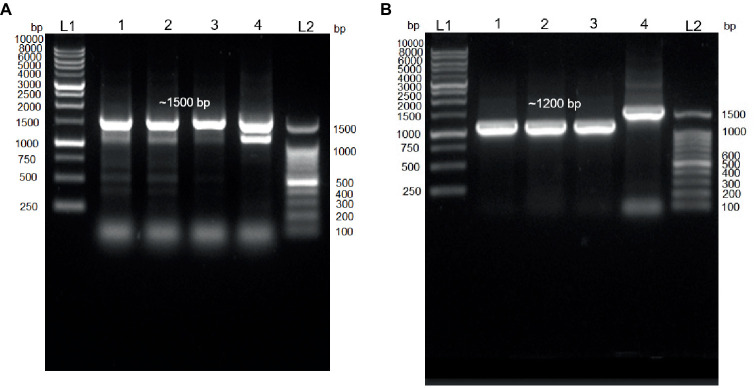
Colony PCR. **(A)** Agarose gel electrophoresis (1.0%) of PCR amplification showing ~1,500 bp of 16S rDNA. Lane 1–3 = Isolated E301, E304, and E311 colonies; Lane 4 = control positive (*Vibrio proteolyticus*); Lane (L1 and L2) DNA ladder of 1 kb and 100 bp, respectively. **(B)** Agarose gel electrophoresis (1.0%) of PCR amplification showing ~1,200 bp of *trpB* gene. Lane 1–3 = Isolated E301, E304, and E311 strains; Lane 4 = control positive (16S rDNA of E311 strain); Lane (L1 and L2) DNA ladder of 1 kb and 100 bp, respectively.

**Figure 4:**
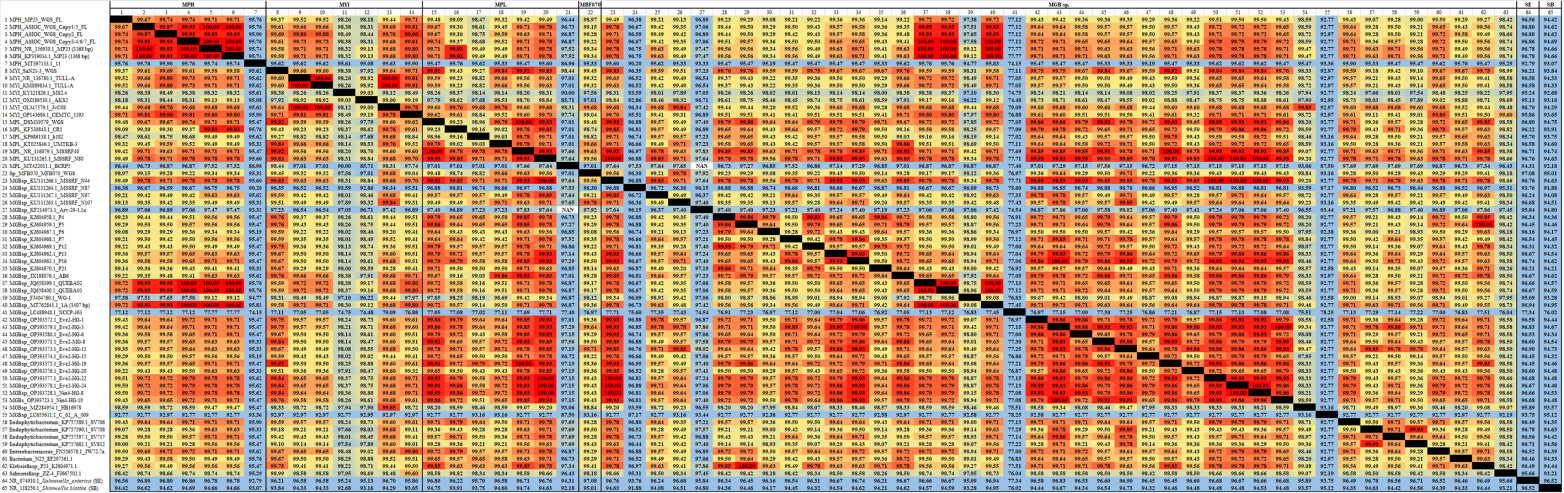
The average nucleotide identity (ANI) similarity index for 16S rDNA among MGBs. The sequences exhibiting the highest and lowest ANI similarity were visually distinguished by the use of dark red and pale blue colors, respectively.

In this study, the 16S rDNA sequences of strains E301, E304, and E311 were acquired using colony PCR (Figure 3A) and, subsequently, analyzed using Sanger sequencing. The isolates displayed a significant degree of similarity to MPH MP23^T^, as evidenced by ANI indices of 100, 99.89, and 99.68% for each corresponding isolate (Figure 2A). The process of determining MGB at the species level solely based on 16S rDNA sequences is complicated, unless a complete and unabbreviated 16S rDNA ANI similarity of 100% is achieved (Figure 4). This is exemplified by the comparison between *Gordonia cholesterolivorans* and *G. sihwensis*, which demonstrates a remarkable 99.9% ANI similarity (Supplementary Figure S3). The utilization of this technology proves to be of great value in instances where conventional taxonomic approaches encounter difficulties in distinguishing creatures that are closely related. It is frequently observed that bacterial species belonging to the families Clostridiaceae and Peptostreptococcaceae exhibit significant sequence homology, reaching up to 99%, in their complete 16S rDNA sequences (Jovel et al., 2016).

#### 3.2.2 Determination of MGB via *trpB* gene sequencing

Alternative taxonomic markers are developed through the utilization of conserved protein-coding genes, such as housekeeping genes. Several housekeeping genes such as DNA gyrase subunit B (*gyrB*) (Poirier et al., 2018) and RNA polymerase subunit B (*rpoB*) (Ogier et al., 2019) have been exploited to assess microbial diversity. From the 4 MGB genomes (MPH MP23^T^, MGB sp. MFB070, MYI SaN21-3 and MPL MSSRF40^T^) submitted to the NCBI database (Supplementary Table S1A), 14 housekeeping genes, namely, chromosomal replication initiator protein DnaA (*dnaA*), CTP synthase (*pyrG*), *rpoB*, chaperonin GroEL (*groL*), protein RecA (*recA*), ATP-dependent Clp protease, ATP-binding subunit ClpX (*clpX*), carbamoyl-phosphate synthase large chain (*carB*), UDP-N-acetylmuramate-L-alanine ligase (*murC*), aminopeptidase N (*pepN*), phenylalanine-tRNA ligase alpha subunit (*pheS*), *gyrB*, RNA polymerase sigma factor RpoD (*rpoD*), chaperone protein DnaK (*dnaK*), and *trpB* (Xu et al., 2014; Liu et al., 2017) were identified from the MGB genome. These genes were subsequently subjected to analysis using multiple sequence alignment and ANI similarity calculation (Clustal Omega v1.2.4), to identify a gene marker that exhibits high discrimination between MPL and MYI, which reveals high similarity in 16S rDNA ANI (99.92%). We found that *pepN* and *trpB* genes show a notable divergence in the ANI index among MGBs (Supplementary Figure S4). It is worth mentioning that MGB sp. MFB070 may be classified as a member of the MPL species, as indicated by their significant similarity in ANI across all 14 housekeeping genes, with an average ANI similarity of 99.06% (Supplementary Figure S4).

A set of primers specifically targeting the *trpB* gene (forward primer 5’-CGTATTTTGGTGAATTCGG-3′ and reverse primer of 5’-CGTGAACCGTGAAAATG-3′), which is known to be present in all MGB species, was intentionally created (Supplementary Figure S5). The resulting PCR amplification using these primers will produce a DNA fragment of approximately 1,200 base pairs in size (Figure 3B), which were subsequently subjected to Sanger sequencing. The isolates E301, E304, and E311 indicated a highly conserved *trpB* gene with a similarity of 99.76% to MPH MP23^T^, which was evaluated using Clustal Omega v1.2.4. Furthermore, the ANI similarity of these isolates, specifically in relation to MPL and MYI, was found to be substantially lower at 95 and 87%, respectively. The application of double-validation, utilizing both 16S rDNA and *trpB* gene sequences, provides additional support for the classification of isolated E301, E304, and E311 as members of the MPH species (Figures 2A,B). The utilization of colony PCR and Sanger sequencing for the analysis of *trpB* and/or *pepN* genes exhibit high discriminatory power for MGB species determination and present a practical method for the identification of MGB at the species level. The PCA analysis of MGB’s 16S rDNA and *trpB* sequences, aligned using the MAFFT algorithm, demonstrated the *trpB* gene’s enhanced species delimitation capabilities, as evidenced by the closer clustering of MGB strains from the same species when *trpB* was used as the identification marker (Supplementary Figure S6). Furthermore, our adoption of the colony PCR technique underscores a critical aspect of reliability and precision compared with alternative methods that require an initial extraction of bacterial gDNA.

### 3.3 Genome of MGBs

#### 3.3.1 WGS of MGBs for contiguous genome assembly

The utilization of genome sequences has the potential to provide insights into the ecological functions and evolutionary standing of MGBs. Thus far, four genomes of MGB have been submitted to the NCBI database. However, there have been limited comprehensive studies conducted on this particular subject. In this investigation, we utilized PacBio SMRT HiFi long-read sequencing technology to sequence the genome (Supplementary Document S2 and Supplementary Figure S7). We are the first to assemble an MGB genome of MPH ASIOC01, via long-read sequencing technology, that showed a high level of contiguity, as shown by the presence of a single contig (Table 2).

**Table 2:** Genome and assembly information of reported MGBs.

Features	*M. phragmitis*	*M. phragmitis*	*M. plantisponsor*	*M. yixingensis*	*Mangrovibacter* sp.
GenBank BioSample ID	SAMN37132003	SAMN05177220	SAMN09064728	SAMN21557958	SAMN02719562
Stain ID	ASIOC01	MP23^T^	MSSRF40^T^	SaN21-3	MFB070
NCBI Taxon ID	1,691,903	1,691,903	451,513	1,529,639	NA
Isolate source	AS	Roots of *Phragmites karka*	Roots of mangrove-associated wild rice	Mangrove rhizosphere sediment	Shrimp aquaculture farm
Lifestyles	Free-living	Endophyte	Endophyte	Probably free-living	Probably free-living
Assembly level	Contig	Contig	Scaffold	Contig	Scaffold
Coarse consistency (%)^a^	99.0	99.0	98.7	99.0	98.7
Fine consistency (%)^a^	96.0	96.8	95.9	96.2	96.0
Completeness (%)^a^	98.0	98.0	98.0	97.9	98.0
Contamination (%)^a^	1.5	0.7	1.5	1.0	1.0
Contigs^a^	1	50	56	25	57
GC Content^a^	50.39 (50.3^1^)	49.91 (50.3^1^)	50.43 (50.1^3^)	49.66 (52.0^2^)	50.4
Contig L50^a^	1	4	7	2	8
Genome length^a^	5,765,145 bp	4,947,475 bp	5,352,990 bp	4,983,067 bp	5,361,575 bp
Contig N50^a^	5,765,145	428,946	356,458	684,126	222,077
CDS^a^	5962/5427^c^	4,981	5,416	4,960	5,435
Repeat regions^a^	183	0	0	0	0
tRNA^a^	82	78	72	73	78
rRNA^a^	22	14	9	8	13
Hypothetical proteins^a^	1,855	1,308	1,422	1,170	1,488
Proteins with functional assignments^a^	4,107	3,673	3,994	3,790	3,947
Proteins with EC number assignments^a^	1,290	1,228	1,276	1,271	1,298
Proteins with GO assignments^a^	1,063	1,008	1,048	1,041	1,075
Proteins with pathway assignments^a^	909	885	929	919	948
Proteins with PATRIC cross-genus family (PGfam) assignments^a^	4,429	3,782	4,070	3,778	4,046
Gene^b^	5,506	4,570	4,943	4,622	4,968
Total number of pathways predicted^b^	401	383	389	400	375
Enzyme reactions^b^	2,126	2037	1998	2,150	1874
Enzymes^b^	1,465	1,393	1,379	1,401	1,503
Transport reactions^b^	207	192	76	208	207
Transporters^b^	555	497	798	568	667
Transcription unit^b^	3,663	2,959	3,138	3,171	3,181
Sequencing technology	PacBio Sequel	Illumina MiSeq	Illumina HiSeq	Illumina	Illumina MiSeq
Deposited date	2023/8/24	2016/6/3	2018/5/31	2021/10/18	2014/6/12
Type strain	MP23^T^	MP23^T^	MSSRF40^T^	TULL-A^T^	MFB070

a Predicted with PATRIC v3.6.12—RAST annotation.

b Predicted with Pathway Tool v26.0—PGAP annotation.

c Prokka annotation.

1 Behera et al., 2017.

2 Zhang et al., 2015.

3 Rameshkumar et al., 2010.

**Figure 5:**
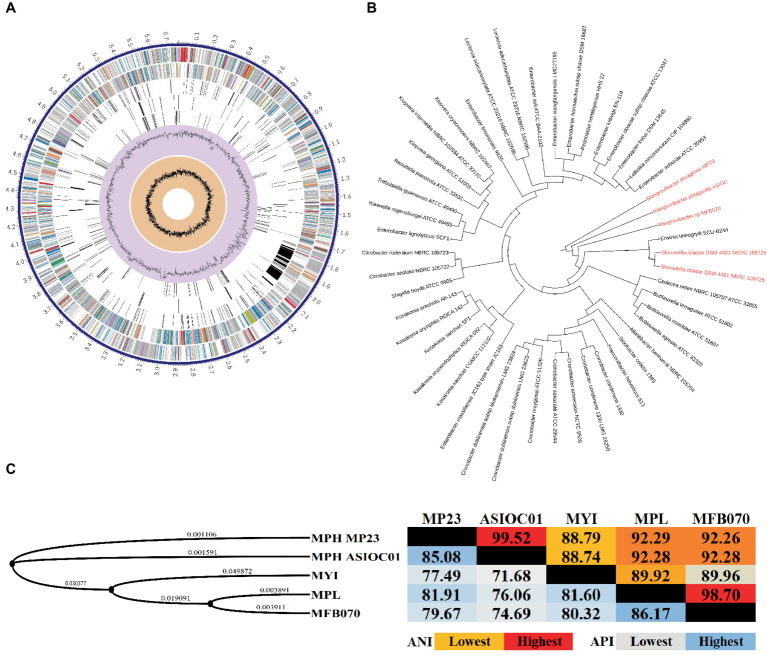
Circular map and phylogenetic analysis of MGB genomes. **(A)** The circular genome map of MPH ASIOC01. The circles represent from the outer circle to the inner circle: the first circle represents the contigs; the second circle represents CDS on the forward strand; the third circle represents CDS on the reverse strand; the fourth circle represents RNA genes; the fifth circle represents CDS with homology to known antimicrobial resistance genes; the sixth circle represents CDS with homology to known virulence factors; seventh circle represents GC content, and the eighth circle represents GC skew. The colors of the CDS on the forward and reverse strands indicate the subsystem these genes belong to (Supplementary Table S2). The genome map was generated using PATRIC v3.6.9 Comprehensive Genome Analysis. **(B)** The evolutionary relationships of the MPH strain ASIOC01 were determined by employing the multi-locus species tree features in AutoMLST program. **(C)** The phylogenetic tree was generated with REALPHY v1.13 and iTOL v6. The calculation of average nucleotide identity (ANI, Right) and average protein identity (API, Left) was performed using QIAGEN CLC Genomics Workbench v22. This result was attained using the “Create Average Nucleotide Comparison tool”.

The genome of MPH ASIOC01 is comprised of 5,765,145 base pairs (bps) and has a GC content of 50.39%, which represented a resemblance to the reported value for MPH MP23^T^ (50.3 mol%) as obtained by the fluorimetric method (Behera et al., 2017). The G + C content of the genome assembly of MPH MP23^T^ was determined to be 49.91% (Behera et al., 2016). A slight variation might be attributed to a systematic underestimation in the high-GC content region (Whibley et al., 2021). The assembly of MPH ASIOC01 is 15% larger in size than the genome of MP23^T^, which consists of 4,947,475 bps due to the short-read assemblies. GC contents predicted from the fluorimetric method (50.3%) were higher compared to the calculated value obtained from WGS (49.91%) indicating that the genome size of MPH MP23^T^ was underestimated. We additionally applied MPH MP23^T^ as a reference genome for QUAST analysis. The resulted BUSCO completeness score was determined at 98.65%, where >95% is regarded as satisfactory. The genome of MPH ASIOC01 comprises of 5,962 complete CDS and 0 incomplete CDS. The dataset consists of 104 RNA genes, specifically comprising 82 transfer RNAs (tRNAs) and 22 ribosomal RNAs (rRNAs), as shown in Table 2. Figure 5A illustrates the genomic map of the MPH ASIOC01 chromosome.

The utilization of WGS data has significantly advanced the taxonomical categorization process, establishing a more reliable technique for species circumscriptions and delineation (Rosselló-Móra and Amann, 2015; Ferraz Helene et al., 2022). By employing WGS data, it becomes feasible to determine the taxonomic classification of MGBs at the species level through the utilization of overall genome-related index (OGRI) analysis (Chun et al., 2018). The evolutionary relationships of MPH ASIOC01 were deduced by employing multi-locus species tree features in the AutoMLST program (Alanjary et al., 2019). A phylogenomic tree was created using maximum-likelihood methodology, utilizing concatenated genes of conserved core proteins. This tree revealed that the phylogenetic position of MPH ASIOC01 was closely related to the type culture MPH MP23^T^ and MGB sp. MFB070. The subsequently observed genus is *Shimwellia*, which is expected given that both MGBs and *Shimwellia* are classified within the subfamilies of the *Enterobacteriaceae incertae sedis* clade (Figure 5B). According to earlier reports, *Shimwellia* and MGBs do not share significant average amino acid identity (API) with any group or species within the Enterobacteriaceae family (Alnajar and Gupta, 2017; Janda and Abbott, 2021). The AutoMLST algorithm, which is used for the automatic generation of species phylogeny (tree building) with reference organisms, also provided a noteworthy suggestion that *Erwinia teleogrylli* SCU-B244 has a close association with MGBs, hence indicating the need for additional inquiry (Figure 5B).

The genome of MPH ASIOC01 was also analyzed using PubMLST in order to determine its species identity. The Ribosomal Multilocus Sequence Typing (rMLST), also known as Species ID feature in PubMLST, uses index variation of selected 53 genes encoding its ribosome protein subunits (*rps* genes) as a means of integrating microbial taxonomy and typing (Jolley et al., 2012). The rMLST inquiry, using the genome of MPH ASIOC01, % supported that MPH ASIOC01 belongs to the taxon of MPH (at the species level).

The delineation of bacterial species through OGRIs relies on the principle that bacteria sharing an average nucleotide identity (ANI) similarity score of 95% or higher are classified within the same species (Richter and Rosselló-Móra, 2009; Chun et al., 2018). The ANI similarity is a crucial metric for OGRIs. The genome level ANI similarity between MPH ASIOC01 and MPH MP23^T^ was determined at 99.52% (Figure 5C). Therefore, MPH ASIOC01 can be confidently classified as a new MPH isolate.

Moreover, it is worth mentioning that, for the species delimitation between strains MGB sp. MFB070 and MPL MSSRF40^T^, the key housekeeping genes (*dnaA*, *pyrG*, *rpoB*, *groL*, *recA*, *clpX*, *carB*, *murC*, *pepN*, *pheS*, *gyrB*, *rpoD*, *dnaK*, and *trpB*) displayed a significant degree of similarity, with an average ANI similarity of 99.06% (Supplementary Figure S4). Molecular typing of the genome of MGB sp. MFB070 using rMLST indicated a 96% likelihood of this strain belonging to MPL, whereas there was a 3% probability of it being an MYI. Finally, MGB sp. MFB070 and MPL MSSRF40^T^ exhibited a genomic ANI (OGRI) similarity of 98.7% (Figure 5C), providing another strong evidence that the MGB sp. MFB070 and MPL belong to the same taxon.

#### 3.3.2 MGBs pan-genome analysis by Roary software

**Figure 6:**
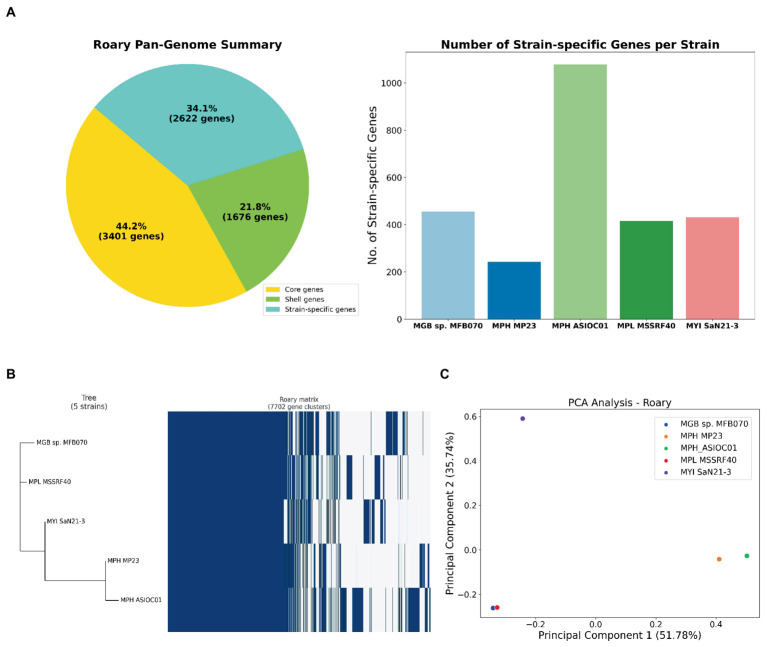
Pan-genome analysis of MGBs. **(A)** Gene distribution in five MGB genomes. The left panel categorizes genes into core, shell, and strain-specific types. Core genes are conserved across all strains, shell genes are present in multiple, but not all strains and strain-specific genes are unique to individual strains. The right panel displays the distribution of strain-specific genes among the five MGBs, extrapolated from the gene presence-absence matrix generated by Roary 3.7.0. MPH ASIOC01 leads with 1,078 unique genes, followed by MGB sp. MFB070, MYI SaN21-3, and MPL MSSRF40^T^ with 456, 431 and 415, respectively. MPH MP23^T^ has the fewest 243 unique genes. **(B)** Phylogenetic analysis and pan-genome matrix of MGBs. The combined representation displays a phylogenetic tree, inferred using FastTree 2.1.9, alongside a pan-genome heatmap generated by Roary 3.7.0. This heatmap visualizes the presence (indicated in blue) and absence (shown in white) of genes across the five strains. Of the 7,702 identified gene clusters, 3,402 are core genes present in all strains, while the shell genes account for the remaining 4,300 clusters. **(C)** PCA on the pan-genome. This PCA plot was constructed using the Bray–Curtis distance method on the gene presence-absence matrix of the five MGB strains. Each point represents a strain, plotted according to the first two principal components, which capture the major variances in the genomes. The proximity of points indicates the similarity in gene content, with MPH ASIOC01 and MPH MP23^T^ appearing closely related, while MGB sp. MFB070 stands distinct, suggesting dissimilar gene profiles.

Roary software tool was employed to assess the pan-genome of 5 MGB strains (Supplementary Table S1A). The pan-genome embodies the entire gene repertoire across all strains. We can classify these gene sets into three categories: core genes, which present in all five strains; shell genes, which was found in multiple but not all strains; and strain-specific genes, which was unique to individual strains. The pan-genome consisted of 7,669 genes (Figure 6A). The core genome, containing 3,401 genes, is conserved across all strains, suggesting that these genes are fundamental to MGB’s essential cellular processes (Tettelin et al., 2005). Our analysis also identified 1,676 shell genes, which was indicative of considerable genomic diversity and potential ecological adaptability (Polz et al., 2013).

A comprehensive set of 2,623 strain-specific genes was identified, where each gene that uniquely associated with 1 of the 5 strains was under investigation (Figure 6A). MPH ASIOC01 has the most abundant strain-specific genes, with approximately 1,078, emphasizing its unique genetic repertoire. The result underscores MPH ASIOC01’s genomic distinctiveness, even compared with MPH MP23^T^. MGB sp. MFB070, MYI SaN21-3, and MPL MSSRF40^T^ follow with approximately 456, 431, and 415 unique genes, respectively. MPH MP23^T^ has the fewest, with approximately 243 unique genes. These genes may contribute to environmental adaptability and virulence of each strain (Wu et al., 2018).

A phylogenetic tree was constructed based on the pan-genome matrix generated by Roary’s output to reveal the evolutionary relationships among the five MGB strains (Figure 6B). From the phylogenetic tree, we observed that the two MPH strains, MPH ASIOC01 and MPH MP23^T^, cluster closely together, indicating a high degree of genomic similarity. The outcome is expected, given that they belong to the same species. The other three strains, namely, MPL MSSRF40^T^, MYI SaN21-3, and MGB sp. MFB070, form separate branches in the tree, reflecting their distinct genomic content. Despite their divergence, all five strains share a common ancestral node, reinforcing their classification within the same genus.

We employed PCA to analyze a distance matrix using the Bray–Curtis dissimilarity metric (Snipen and Liland, 2015), which was derived from Roary’s gene presence/absence matrix generated with 95% BlastP identity threshold (Figure 6C). This method helps discern high-dimensional genomic patterns and visualize variance. Genomes with similar gene sets are closely clustered, while divergent ones are distant. Notably, despite being the same species, MPH ASIOC01 and MPH MP23^T^ are slightly apart in the PCA plot, suggesting significant gene content variation possibly due to the adaptation to different environments or genome sequence quality. Conversely, MPL MSSRF40^T^ and MGB sp. MFB070 are close on the PCA plot, implying similar gene repertoires and potentially similar metabolic capabilities or ecological niches. The data are consistent with ANI data of the WGS and housekeeping gene analysis. This result was consistent with the PCA plot outcome which was generated using *trpB* sequences described earlier (Supplementary Figure S6).

#### 3.3.3 KEGG pathway and subsystem analysis

**Figure 7:**
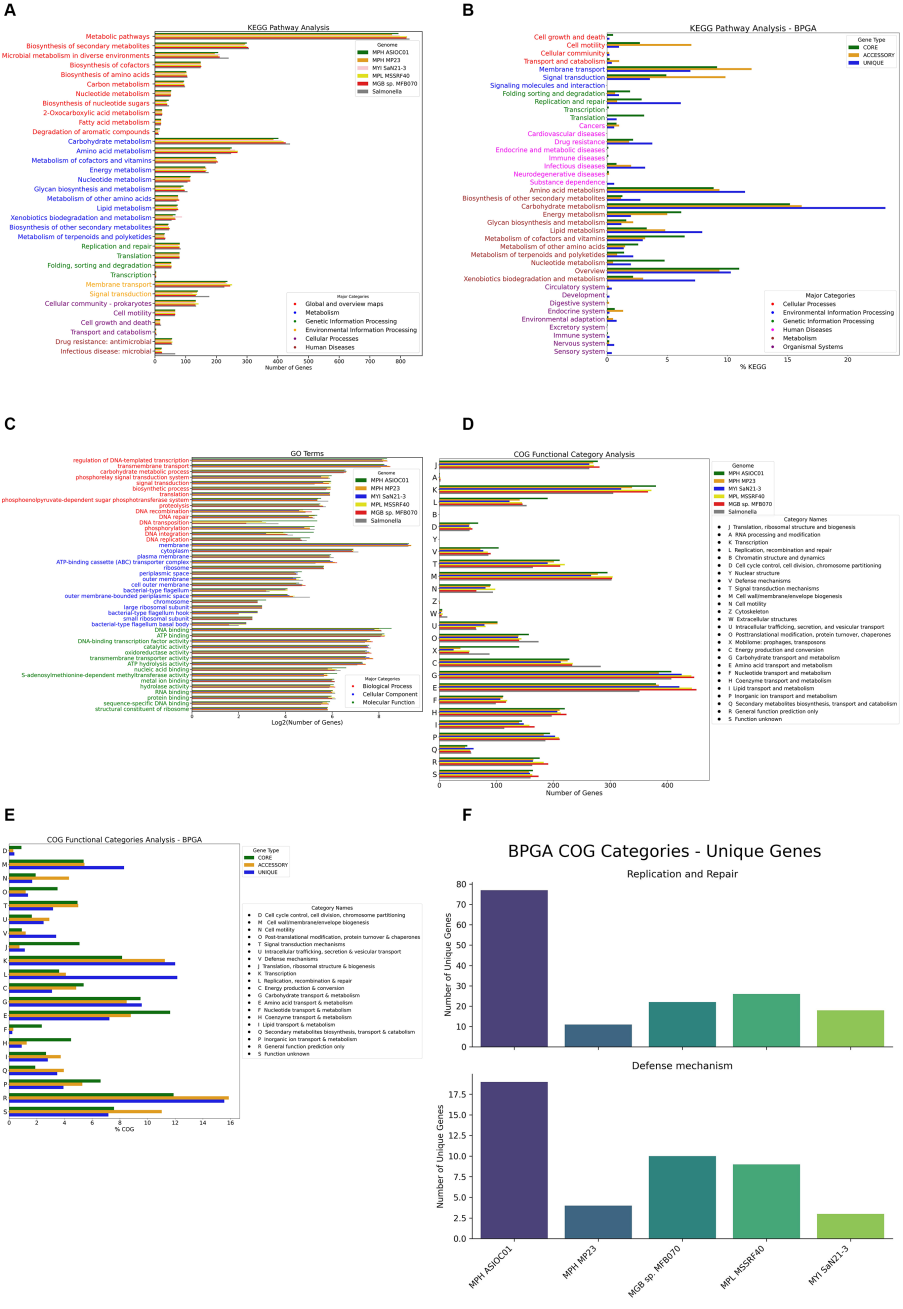
Gene annotation and analysis. **(A)** KEGG pathway analysis for MGBs. Each bar represents the number of genes associated with a particular KEGG pathway. The pathways are broadly categorized into metabolism, genetic information processing, environmental information processing, cellular processes, organismal systems, and human diseases. **(B)** KEGG pathway analysis for the pan-genome. This histogram presents the KEGG pathway analysis for the pan-genome of the five MGB strains, as performed by the BPGA tool. **(C)** Gene ontology (GO) analysis for MGBs. **(D)** Clusters of orthologous groups (COG) category analysis. **(E)** COG category analysis of the pan-genome of the five MGB strains, as performed by the BPGA tool. Each bar represents the % COG genes associated with a particular COG functional category. **(F)** Strain-specific gene count in “replication and repair” and “defense mechanisms” COG categories. The facet-grid chart depicts the number of unique genes associated with the “replication and repair (L)” COG category (on the top panel) and “defense mechanisms (V)” COG category (on the bottom panel) in each of the five MGBs’ genomes extrapolated from the BPGA analysis.

KEGG pathway analysis indicated that the most significant number of KO-annotated genes was related to the metabolism pathway, in which carbohydrate metabolism had the highest gene count (402 genes) (Figure 7A). A subsystem is a set of functional roles that implement a specific biological process or structural complex (Overbeek et al., 2005). The sub-system distribution of MGBs is presented in Supplementary Table S2. The genome annotation of MPH ASIOC01 performed by RAST (BV-BRC Server) revealed the subsystem feature counts that the major categories of protein-coding regions are related to metabolism (38%), energy (14%), and protein processing (10%).

We further investigated the functional potential of the five MGB genomes using Bacterial Pan Genome Analysis Pipeline 1.3 (BPGA) for pan-genome KEGG pathway analysis (Figure 7B). Our findings show high conservation in carbohydrate and amino acid metabolism pathways across all genomes, similar to observations in *Salmonella typhi* (Katiyar et al., 2020), which has the closest 16S rDNA ANI similarity compared with MGBs. A significant number of unique genes for MPH ASIOC01, particularly in pathways such as amino acid, carbohydrate, and lipid metabolism, as well as replication, repair, and xenobiotic biodegradation, are dominant and indicate that the microorganism can utilize xenobiotic pollutants as nitrogen or carbon sources for growth and render its metabolic activity (Mishra et al., 2021). The corresponding result aligns with the concept of genome plasticity, suggesting that MGBs adapt to environments with toxic pollutants by gaining or losing genes. Our analysis also highlighted the significant contribution of accessory genome to other pathways, including membrane transport and signal transduction, supporting the idea of genome plasticity and ecological adaptability (Ariute et al., 2022).

#### 3.3.4 GO and COG category analysis

The GO terms enriched in the biological process were regulation of DNA-templated transcription (325 genes), transmembrane transport (301 genes), and carbohydrate metabolic process (95 genes). Cellular components were dominated by the membrane component (624 genes), followed with cytoplasm (120 genes) and plasma membrane (62 genes). With regard to GO terms for molecular functions, the top three were DNA binding (372 genes), ATP binding (307 genes), and DNA-binding transcription factor activity (197 genes) (Figure 7C).

In total, 4,067 out of 5,247 genes (78.06%) were annotated with COG functional category, in which the top categories were carbohydrate transport and metabolism (9.93%), transcription (9.27%), and amino acid transport and metabolism (9.27%). By compiling the annotation results of the five MGBs along with *Salmonella* sp. as the out-group, we found out that, despite most of the annotation results matching up, MPH ASIOC01 had enriched COG category in Mobilome [X], which includes prophages and transposons. This discovery corresponded to GO annotations, which had relatively higher gene numbers in DNA recombination, transposition, integration, and binding (Figure 7D). This outcome might indicate that our isolate had more HGT events due to the facilitated activities of mobile genetic elements (MGEs) in AS (Zhang et al., 2011; Yang et al., 2013). As an alternative, this observation could also be the consequence of our sequencing method: The short repeats and highly similar sequence of MGEs might pose a challenge in short read assembly (Alkan et al., 2011; Li et al., 2015), resulting in lower copy numbers of MGE in the other four MGB genomes. On the other hand, such short repeats were more likely to be preserved in PacBio sequencing (Teng et al., 2017).

We conducted a COG category analysis using BPGA to further probe the functional profiles of MGB genomes (Figure 7E). The analysis shows that core genes are highly conserved in categories such as “translation, ribosomal structure, and biogenesis” [J], “amino acid transport and metabolism” [E], and “energy production and conversion” [C], emphasizing their role in essential biological processes and environmental survival. Notably, MPH ASIOC01 has a significant number of unique genes in “replication and repair” [L] and “defense mechanisms” [V], which could be key to its adaptive strategies. These include critical molecular entities such as DNA-3-methyladenine glycosylase and DNA adenine methylase in “replication and repair” and omega-amidase YafV and multidrug ABC transporter permease YbhS in “defense mechanisms” (Figure 7F).

Salt shock exposure was observed to induce an increase in ‘defense mechanism’ genes within the COG category in *Mesorhizobium loti* MAFF303099, with a notable overexpression of 5 out of 67 genes in this category, representing approximately 7.5% of the genes involved (Laranjo et al., 2017). This upregulation is indicative of a transcriptional response to osmotic stress, suggesting that a similar adaptive response may be occurred in MPH ASIOC01. Our COG category analysis revealed that MPH ASIOC01 harbors 104 genes associated with ‘defense mechanism,’ surpassing the 72 genes identified in MPH MP23^T^ and representing the greatest number of genes within this category across the five genomes studied (Figure 7D). Furthermore, our BPGA pan-genome analysis delineated that ‘defense mechanism’ genes constitute 3.4% of the unique genomic repertoire, while accessory and core genomes encompass only 1.2 and 0.9%, respectively, in this category. Notably, MPH ASIOC01 possesses the highest number (counts) of unique ‘Defense Mechanism’ genes in the pan-genome (Figures 7E,F), suggesting MPH ASIOC01’s unique genes in this category could be a response to the high-salinity (approx. 3% NaCl), pollutant-rich environment of the membrane bioreactor acrylonitrile (MBR_AN) AS system from which it was isolated, supported by our *in vitro* findings that MPH ASIOC01 efficiently propagates in mediums containing 8% NaCl (Figure 1F). The difference in unique genes in “defense mechanisms” between MPH MP23^T^ and MPH ASIOC01 indicates divergent adaptive strategies possibly due to their different environments. MPH MP23^T^, isolated from *Phragmites karka* roots, has fewer unique genes in this category, suggesting less selective pressure for robust defense mechanisms. This also hints at genome content variations between free-living and plant-associated MGBs.

#### 3.3.5 Comparative genomic analysis using BRIG

**Figure 8:**
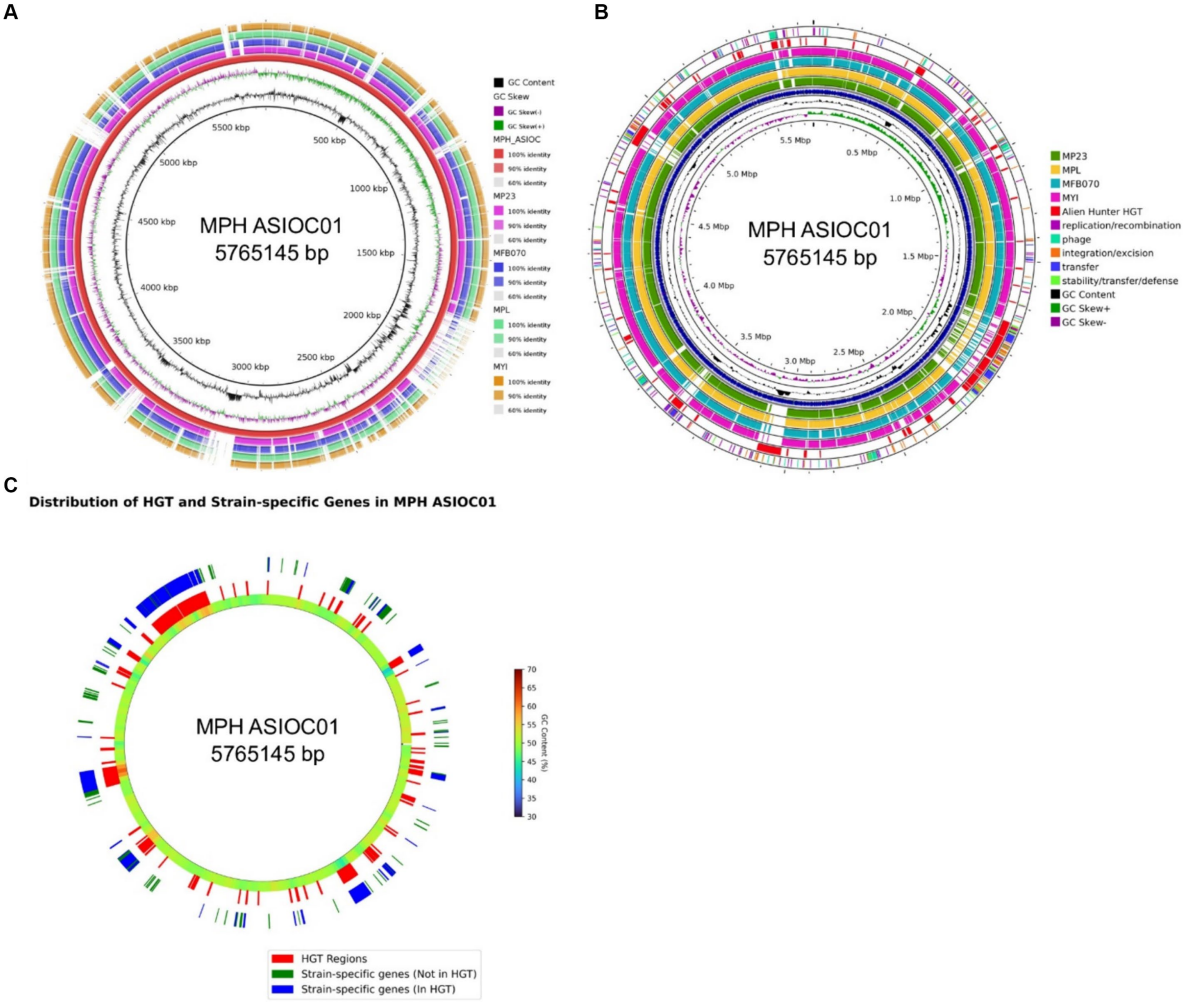
BRIG analysis. **(A)** Circular comparison of MGB genomes using BLAST Ring Image Generator (BRIG). Each concentric ring represents a genome, with MPH ASIOC01 as the reference genome. The genomes, in order from the inner to outer ring, are MPH ASIOC01, MPL MSSRF40^T^, MGB sp. MFB070, MPH MP23^T^, and MYI SaN21-3. The color gradient in each ring represents the BLAST identity percentage to the reference genome. Darker regions indicate high identity (90% or more), lighter regions indicate moderate identity (between 60 and 90%), and the white regions indicate sequences unique to MPH ASIOC01. **(B)** Circular comparison of MGB genomes with Proksee web-based ring generator. The figure displays a graphical representation of the five MGB genomes. MPH ASIOC01 is the reference genome. The genomes, in order from the inner to outer ring, are MPH ASIOC01 (Blue), MPL MSSRF40^T^, MGB sp. MFB070, MPH MP23^T^, and MYI SaN21-3. Expect value cutoff was set at 0.1. The penultimate ring represents the predicted regions of HGT in MPH ASIOC01 determined by the Alien Hunter plugin in the web-based Proksee tool. The outermost ring represents the various mobilome regions of MPH ASIOC01 identified using mobileOG-db plug-in from Proksee server. **(C)** Circular comparison of strain-specific gene regions within the MPH ASIOC01 genome against HGT regions. The innermost ring delineates the genome according to GenBank annotations, with GC content represented through a gradient scale variation in color intensity directly correlate with fluctuations in GC content throughout the genome. The middle ring highlights genomic regions associated with HGT in red, as inferred from the Alien Hunter plugin. The outermost ring maps strain-specific genes: those not located within HGT regions are marked in green, while strain-specific genes situated within HGT regions are depicted in blue.

To visualize the genomic architecture and sequence distribution in MGB genomes, the Blast Ring Image Generator (BRIG) was employed, with MPH ASIOC01 serving as the reference (Figure 8A). The BRIG plot highlights conserved regions across genomes, indicating functionally important and evolutionarily conserved elements. Deviations in GC content suggest incorporation of external genetic material, serving as a record of past genomic events such as HGT, inversions, and plasmid integrations (Zhang et al., 2014; Hubert, 2022). Overlaying this on our ProkSee plot (Figure 8B), the penultimate ring, representing predicted HGT regions by the Alien Hunter plugin (Vernikos and Parkhill, 2006), hints at diverse donor organisms with distinct GC content. These HGT regions (Figure 8A) often coincide with gaps in other genome rings, suggesting that MPH ASIOC01 has more horizontally transferred genes. The result could explain why MPH MP23^T^ is slightly distant from MPH ASIOC01 in the PCA plot (Figure 6C).

We also utilized the mobileOG-db plugin within Proksee (Figure 8B) to delineate regions associated with mobile elements in MPH ASIOC01. These regions contain a part of the mobilome, which is crucial for functions, such as DNA replication, recombination, repair, and transfer. In the MGB context, they indicate areas where genome of MPH ASIOC01 might have undergone significant shuffling, potentially acquiring new genes or functionalities. Evaluating these mobile genetic elements (MGEs) is vital for understanding antibiotic resistance origins, phenotypic variability, and evolutionary patterns (Brown et al., 2022). It is worth noting that a distinct convergence exists between the regions identified as probable HGT events by the Alien Hunter tool and revealed by the mobileOG-db database. Among the 1,078 strain-specific genes observed in MPH ASIOC01, 794 (73.65%) were localized within regions of the genome predicted to be associated with HGT. This overlap suggests that most of the strain-specific genes in MPH ASIOC01 could have been acquired during HGT events (Figure 8C). This observation emphasizes the noteworthy prevalence of HGT incidents associated with the MPH ASIOC01 genes.

### 3.4 Genes conferring key functional traits for MGBs

#### 3.4.1 Glycerol degradation

**Figure 9:**
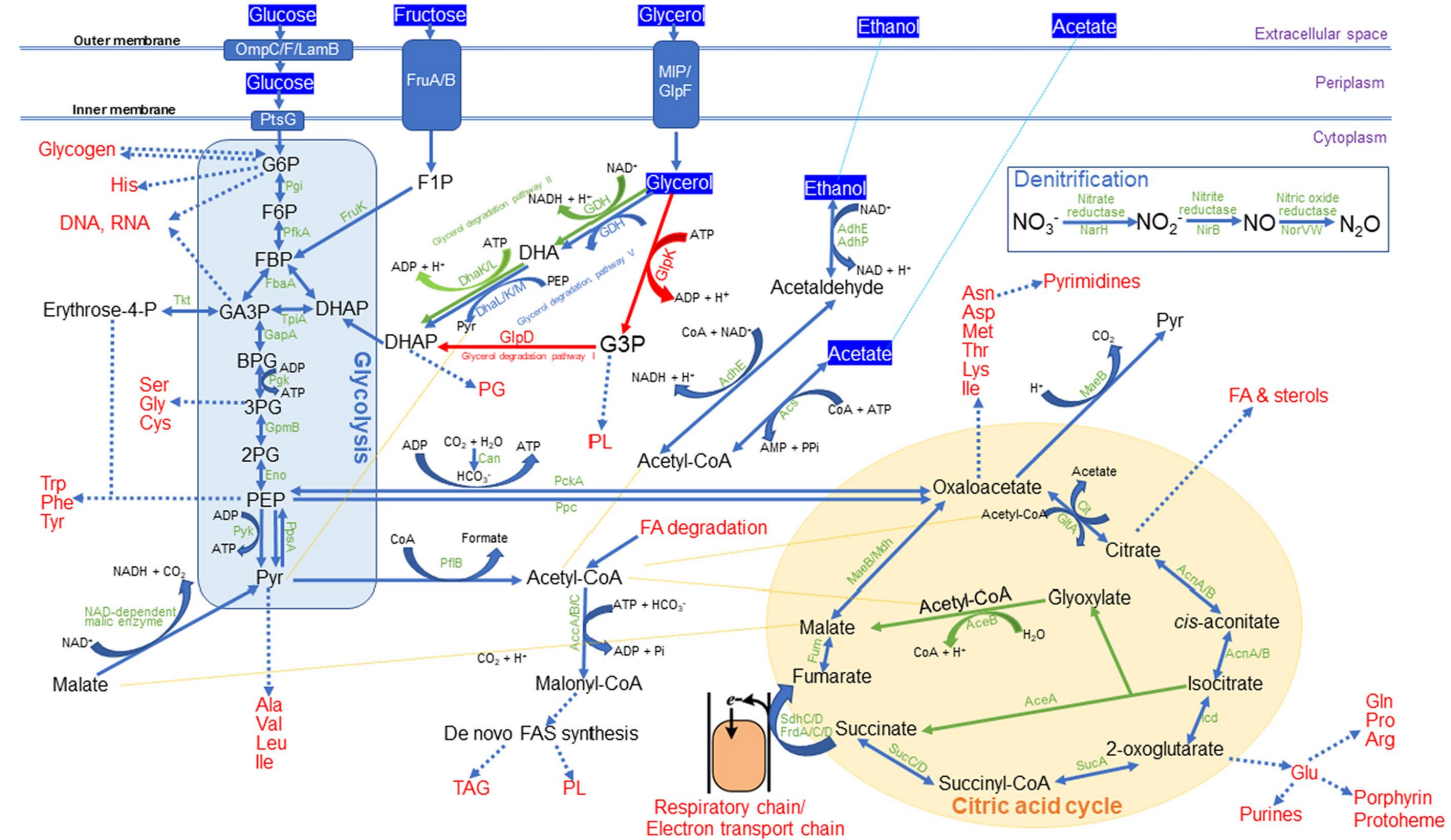
Predicted carbon assimilation and dissimilation patterns in MPH ASIOC01.

Examination of MPH ASIOC01’s WGS data provides insights into the genetic characteristics that have a direct or indirect impact on its biological activity, including its capacity to thrive in wastewater with high glycerol content. The MBR, where MPH ASIOC01 was isolated, is employed for treating residual ECH discharged from manufacturing pipelines. The initially carcinogenic ECH compounds were subsequently transformed into non-toxic glycerol and NaCl through the process of low-pressure alkaline hydrolysis (McGrath et al., 2012). Hence, the presence of glycerol at approximately 2.8% and NaCl at approximately 3% was detected in the MBR-ECH wastewater. The capacity of MPH ASIOC01 to survive in ECH wastewater is probably due to its acquisition of a comprehensive glycerol degradation system, including citric acid cycle, glycerol degradation I, II, and V, and gluconeogenesis I and glycolysis III (as shown in Figure 9 and Supplementary Figure S8). The presence of the toxic compounds’ dissimilation processes in MPH ASIOC01 suggests that this MPH variant has undergone environmental adaptation, allowing it to effectively utilize glycerol and thrive in this particular wastewater environment. Figure 9 demonstrates the carbon assimilation by MPH AISOC01 when utilizing glycerol or other exclusive carbon sources. The enzymes responsible for incorporating fructose, acetate, and ethanol into the major metabolic pathways are also shown in Figure 9. It is noteworthy that MPH ASIOC01 may convert pyruvate (C3) to oxaloacetate (C4) with the aid of phosphoenolpyruvate carboxylase (Ppc), phosphoenolpyruvate carboxykinase (PckA), and carbonic anhydrase (Can). In addition, the key enzymes for denitrification, such as nitrate reductase (NarH), nitrite reductase (NirB), and nitric oxide reductase (NorVW), were also found in the genome of MPH ASIOC01. Therefore, MPH ASIOC01 might serve as a denitrifying PAO (DPAO) in the AS system. The initial findings indicate that MPH ASIOC01 has a notable capacity for rapid biomass accumulation when cultivated on a medium with high phosphate content, such as M9 or 910 P1 mineral salt medium, utilizing glycerol as the sole carbon source. MPH ASIOC01 has the ability to utilize many types of sole carbon sources, including glucose, fructose, acetate, and ethanol. It is worth mentioning that the genomes of MPH MP23^T^, MYI SaN21-3, MPL MSSRF40^T^, and MGB sp. MFB070 shared a similar glycerol degradation pathway, as shown in Table 3. This observation suggests that glycerol utilization is a shared characteristic among MGBs. Therefore, it is feasible to enhance the growth of MGB variants in wastewater with glycerol derivatives, such as 1,3-DCP and 3-MCPD, as carbon source.

**Table 3:** Genotype–phenotype correlations of MGBs.

MPH ASIOC01						Present/absent or copy numbers in genome				
No	Phenotype	Locus tag/pathways^a^	Gene ID^a^	AA length^a^	Gene function^a^	ASIOC01^b^	MP23^b^	MPL^b^	MYI^b^	MFB070^b^
1	Glycerol utilization	Glycerol degradation I	N/A	N/A	Glycerol utilization	Yes	Yes	Yes	Yes	Yes
2	Glycerol utilization	Glycerol degradation II	N/A	N/A	Glycerol utilization	Yes	Yes	Yes	Yes	Yes
3	Glycerol utilization	Glycerol degradation V	N/A	N/A	Glycerol utilization	Yes	Yes	Yes	Yes	Yes
4	Cellulose-degradation	MPHASIOC01_000290	*bcsZ*	369	Cellulase	1	1	1	1	1
5	Cellulose-degradation	MPHASIOC01_000013	NA	299	Cellulase family glycosylhydrolase	1	1	1	1	2
6	Cellulose-degradation	MPHASIOC01_002426	*bglX*	765	β-glucosidase	1	1	1	1	1
7	Cellulose-degradation	MPHASIOC01_000282, 000135	NA	333	Glycosyl hydrolase family 8/Endoglucanase	2	2	2	2	2
8	Cellulose biosynthesis	MPHASIOC01_000141, 000278, 000288	*bcsA*	876	UDP-forming cellulose synthase catalytic subunit	3	3	3	3	3
9	Cellulose biosynthesis	MPHASIOC01_000279, 000289	*bcsB*	793	Cellulose biosynthesis cyclic di-GMP-binding regulatory protein	2	2	2	2	2
10	Cellulose biosynthesis	MPHASIOC01_000291	*bcsC*	1,177	Cellulose synthase complex outer membrane protein	1	1	1	1	1
11	Cellulose biosynthesis	MPHASIOC01_000138, 000281	*bcsD*	159	Cellulose synthase	2	2	2	2	2
12	Cellulose biosynthesis	MPHASIOC01_000285	*bcsE*	520	Cellulose biosynthesis protein	1	1	1	1	1
13	Cellulose biosynthesis	MPHASIOC01_000284	*bcsF*	65	Cellulose biosynthesis protein	1	1	1	1	1
14	Cellulose biosynthesis	MPHASIOC01_000283	*bcsG*	554	Cellulose biosynthesis protein	1	1	1	1	1
15	Cellulose biosynthesis	MPHASIOC01_000276	*bcsO*	252	Cellulose biosynthesis protein	1	1	1	1	1
16	Cellulose biosynthesis	MPHASIOC01_000277, 000287	*bcsQ*	267	Cellulose biosynthesis protein	2	2	2	2	2
17	Cellulose biosynthesis	MPHASIOC01_001652	*dgcQ*	552	Cellulose biosynthesis regulator diguanylate cyclase	1	1	1	1	1
18	Nitrogen-fixation	MPHASIOC01_000881	*nifA*	523	nif-specific transcriptional activator	1	1	1	1	1
19	Nitrogen-fixation	MPHASIOC01_000882	*nifB*	472	Nitrogenase cofactor biosynthesis protein	1	1	1	1	1
20	Nitrogen-fixation	MPHASIOC01_000866	*nifD*	482	Nitrogenase molybdenum-iron protein alpha chain	1	1	1	1	1
21	Nitrogen-fixation	MPHASIOC01_000870	*nifE*	458	Nitrogenase iron-molybdenum cofactor biosynthesis protein	1	1	1	1	1
22	Nitrogen-fixation	MPHASIOC01_000865	*nifH*	293	Nitrogenase iron protein	1	1	1	1	1
23	Nitrogen-fixation	MPHASIOC01_000864, 001342	*nifJ*	1,177	Pyruvate: ferredoxin (flavodoxin) oxidoreductase	2	2	2	2	2
24	Nitrogen-fixation	MPHASIOC01_000867	*nifK*	521	Nitrogenase molybdenum-iron protein subunit beta	1	1	1	1	1
25	Nitrogen-fixation	MPHASIOC01_000880	*nifL*	497	Nitrogen fixation negative regulator	1	1	1	1	1
26	Nitrogen-fixation	MPHASIOC01_000878	*nifM*	270	Nitrogen fixation protein	1	1	1	1	1
27	Nitrogen-fixation	MPHASIOC01_000871	*nifN*	461	Nitrogenase iron-molybdenum cofactor biosynthesis protein	1	1	1	1	1
28	Nitrogen-fixation	MPHASIOC01_000874	*nifS*	402	Cysteine desulfurase	1	1	1	1	1
29	Nitrogen-fixation	MPHASIOC01_000873	*nifU*	278	Fe-S cluster assembly protein	1	1	1	1	1
30	Nitrogen-fixation	MPHASIOC01_000875	*nifV*	378	Homocitrate synthase	1	1	1	1	1
31	Inorganic phosphate-solubilizing	MPHASIOC01_002786	*ppx*	514	Exopolyphosphatase	1	1	1	1	1
32	Inorganic phosphate-solubilizing	MPHASIOC01_004901	*ppa*	176	Inorganic diphosphatase	1	1	1	1	1
33	Inorganic phosphate-solubilizing	MPHASIOC01_004482	*gcd*	794	Glucose/quinate/shikimate family membrane-bound PQQ-dependent dehydrogenase	1	1	1	1	1
34	Organic phosphorus mineralizing	MPHASIOC01_004203	*phoA*	469	Alkaline phosphatase	1	1	1	1	1
35	Phosphate transporter	MPHASIOC01_000317	*pitA*	497	Inorganic phosphate transporter	1	1	1	1	1
36	Phosphate transporter	MPHASIOC01_005489, 005494	*pstA*	296	Phosphate ABC transporter permease	2	1	1	1	1
37	Phosphate transporter	MPHASIOC01_005490, 005495	*pstB*	257	Phosphate ABC transporter ATP-binding protein	2	1	1	1	1
38	Phosphate transporter	MPHASIOC01_000377	*ugpQ*	246	Glycerophosphodiester phosphodiesterase	1	1	1	1	1
39	Phosphate intake regulatory	MPHASIOC01_004193	*phoB*	229	Phosphate response regulator transcription factor	1	1	1	1	1
40	Phosphate intake regulatory	MPHASIOC01_004192	*phoR*	436	Phosphate regulon sensor histidine kinase	1	1	1	1	1
41	PolyP synthesis	MPHASIOC01_002785	*ppk1*	686	PolyP kinase 1	1	1	1	1	1
42	Cr reduction	MPHASIOC01_000547	*nfsA*	240	Oxygen-insensitive NADPH nitroreductase	1	1	1	1	1
43	Cr reduction	MPHASIOC01_001267	*nfsB*	214	Oxygen-insensitive NAD(P)H nitroreductase	1	1	1	1	1
44	Cr reduction	MPHASIOC01_001090	*nemA*	365	Alkene reductase (Blast)/N-ethylmaleimide reductase (Pathway Tools)	1	1	1	1	1
45	Dye degradation	MPHASIOC01_001328	*azrG*	199	FMN-dependent NADH azoreductase	1	1	1	1	1

a Information from Pathway Tools.

b Locus tags for all MGB strains are presented in Supplementary Table S8.

#### 3.4.2 Nitrogen fixation

According to earlier reports, MGBs are nitrogen-fixing bacteria that thrive in a nitrogen-free environment (Rameshkumar et al., 2010; Tam et al., 2019). MYI TULL-A^T^ (Zhang et al., 2015), MPH MP23^T^ (Behera et al., 2017), MPL MSSRF40^T^ (Rameshkumar et al., 2010), and MGB sp. MFB070 (Joseph et al., 2014) were also reported as nitrogen-fixing bacteria. The constituent parts of nitrogenase enzyme complex are encoded by the bacterial *nif* genes. The *nifH*, *nifD*, and *nifK* genes encode the structural subunit of dinitrogenase reductase and the two subunits of dinitrogenase (Dai et al., 2014). Notably, the genome of MGBs (Table 3) contained a complete set of known *nif* genes (*nifA*, *nifB*, *nifD*, *nifE*, *nifH*, *nifJ*, *nifK*, *nifL*, *nifM*, *nifN*, *nifS*, *nifU*, *nifV*, and *nifQ*) which were found to be closely clustered in the region between 917,880 and 941,477 in the genome map position of MPH ASIOC01. Therefore, it is justifiable to make a generalization that nitrogen fixation is a common trait observed among MGBs.

#### 3.4.3 Phosphate solubilization

The utilization of NBRIP medium, originally designed for the screening of phosphate-solubilizing bacteria, was found to be effective in the isolation of MGBs (Nautiyal, 1999; Tam et al., 2019). The genes implicated in the phosphorus cycle encompass several processes, such as inorganic phosphate solubilization (e.g., *gcd*, *ppa*, and *ppx*), organic phosphorus mineralization (e.g., *phoA* and *phoD*), transporters (e.g., pit, *pstA*, *pstB*, and *ugpQ*), and regulatory genes (e.g., *phoB* and *phoR*) (Wu et al., 2022). The above-mentioned genes, namely, *gcd*, *ppa*, *ppx*, *phoD*, *pitA*, *pstA*, *pstB*, *ugpQ*, *phoB,* and *phoR* were found in all MGB genomes (Table 3). The findings validate the capacity of MGBs to solubilize, import, and metabolize phosphate from their external surroundings. The identification of polyP kinase 1 (refer to Table 3) within the MGB genomes, responsible for PolyP accumulation, underscores its crucial function in phosphate storage.

#### 3.4.4 Chromium reduction

Cr(VI) has been identified as a hazardous waste and requires appropriate treatment prior to its disposal. According to recent studies conducted by Sanjay et al. (2020) and Lian et al. (2016a,b), it has been demonstrated that MYI and MPL have the capability to effectively carry out the bio-reduction process of Cr(VI). These findings indicate a significant potential for the use of MYI and MPL in the field of environmental bioremediation. Previous studies have proposed that nitroreductases (specifically NfsA and NfsB) derived from *Vibrio harveyi* and *E. coli* (Kwak et al., 2003) and N-ethylmaleimide reductase (NemA) from *E. coli* exhibit notable efficacy as chromate reductases (Robins et al., 2013). The presence of *nfsA*, *nfsB*, and *nemA* genes in the genome of MGBs is a noteworthy observation, as shown in Table 3. The enzymes NfsA, NfsB, and NemA, which are present in MPH ASIOC01, with high identity to the other MGBs, demonstrate a significant similarity in protein size compared with their corresponding orthologs in *E. coli*. The level of similarity in the API between NfsA, NfsB, and NemA and their respective counterparts in *E. coli* is 77.5, 49.1, and 77.3%, respectively. Therefore, it implicate that all MGBs possess the ability to reduce Cr.

#### 3.4.5 Azo dye remediation

According to Nasrin et al. (2022), it has been proposed that the MYI strain AKS2 can break down Basic Red-18 dye (BR-18). BR-18 is a cationic azo dye commonly employed for textile coloring purposes. Azo dyes used in the textile industry have been found to possess toxicological properties, including carcinogenic and mutagenic effects (Zahran et al., 2019; Nasrin et al., 2022). Nevertheless, using acid dyes containing azo groups remains highly prevalent within the leather or tannery sector (Christovam et al., 2022). Azoreductases have been identified as enzymes capable of facilitating the degradation of azo dyes. This enzyme has been widely employed within the pharmaceutical, food, cosmetic, and textile sectors. It catalyzes the reductive cleavage of azo bonds (–N=N–) to give colorless aromatic amine (Zahran et al., 2019). FMN-dependent NADH azoreductase (AzrG) is present in the genome of all MGBs, as shown in Table 3. This finding suggests a potential bioremediation role of MGBs in textile and tannery effluent (Lian et al., 2016b; Sanjay et al., 2020).

#### 3.4.6 Carbohydrate-active enzyme (CAZyme) analysis

The analysis of CAZyme annotations using dbCAN-sub (HMMER) revealed the presence of 122 CAZyme genes in the genome of MPH ASIOC01 (Supplementary Table S3). These genes constitute approximately 2.29% of the total coding genes in the genome. These enzymes that play a crucial role in the metabolism of complex carbohydrates, specifically those carbohydrate-active enzymes, were analyzed using HMMER and DIAMOND platforms. These algorithms were employed to search against the dbCAN HMM, CAZy pre-annotated CAZyme sequences, and conserved CAZyme short peptide database. The analysis was conducted using the web-based dbCAN3 server (Zheng et al., 2023). In the present investigation, the results obtained using dbCAN-sub (HMMER) were selected for further analysis as previous reports suggested that the DIAMOND + CAZy search bears a higher likelihood of inaccurate CAZyme family annotation (Zhang et al., 2018). The genomic investigation conducted with the CAZymes database has predicted the existence of numerous enzymes engaged in carbohydrate metabolism. The analysis has identified the existence of 53 glycoside hydrolases (GHs), 43 glycosyl transferases (GTs), 8 carbohydrate-binding modules (CBMs), 7 polysaccharide lyases (PLs), 8 carbohydrate esterases (CEs), and 5 auxiliary activities (AAs) in the genome of MGBs, on average. The data presented in Supplementary Figure S9 represent the mean values derived from the analysis of five MGB genomes. In the context of MPH ASIOC01, the main components consist of GHs, GTs, and CBMs, making up 47.5, 35.0, and 7.5%, respectively, of the total annotated CAZymes. The most prevalent GH and GT genes detected in the MPH ASIOC01 dataset are GH23 (19.3%) and GT2 (33.3%), respectively.

The genome annotation has revealed that all MGBs possess genes that encode for cellulase and enzymes involved in cellulose biosynthesis, as shown in Table 3. Consequently, one might deduce that MGBs could be cellulotrophs. Recently, two novel MGB isolates, namely, strains Eve2-HG and Nin4-HG (GenBank accession nos. OP393571–OP393580), have been demonstrated to possess the capability of cellulose digestion. The genome of MGBs contained four enzymes, namely α-glucosidase, glycosyl hydrolase family 8 (endoglucanase, cellulase family D), cellulose synthase complex periplasmic endoglucanase, and glycosyl hydrolase family A (cellulase family A) which were likely involved in the degradation of cellulose (Table 3 and Supplementary Table S4). The main enzymes involved in the breakdown of cellulose are cellulase and endoglucanases. They are crucial in enabling the endophyte’s penetration into the plant roots (Li et al., 2023). Additionally, Table 3 shows that MGBs contain a complete gene cluster for cellulose biosynthesis that includes *bcsA*, *bcsB*, *bcsC*, *bcsD*, *bcsE*, *bcsF*, *bcsG*, *bcsO*, *bcsQ*, and *dgcQ*. UDP-forming cellulose synthase catalytic subunit (BcsA) and cellulose biosynthesis cyclic di-GMP-binding regulatory protein (BcsB) were also found in the genome of MPH ASIOC01 (Table 3). The compressive array of cellulose biosynthesis proteins probably enables the synthesis of bacterial cellulose (BC). The notable physicochemical properties of BC include its porosity, mechanical and tensile strength, elasticity, transparency, high degree of polymerization, nanostructure, purity, water retention capacity, biodegradability, and biocompatibility. It is also non-cytotoxic and non-genotoxic. Hence, BC is utilized across various industries (Katyal et al., 2023).

#### 3.4.7 Genome mining for secondary metabolites and bacteriocins

Using antiSMASH version 7.0.0, the genomes of MGBs were examined for the presence of secondary metabolites (Blin et al., 2023). Four potential BGCs were forecasted to exist in all MGB genomes. These clusters are members of the thiopeptide, arylpolyene, NRP-metallophore, and RiPP-like compound families (Supplementary Table S5). The aryl polyene (APE) biosynthetic gene pathway (Region 3) of MPH showed 100% similarity to the APE genes from *Xenorhabdus doucetiae*, according to antiSMASH analysis. Host-associated bacteria, which include commensals and diseases that affect human, animals, and plants, are frequently known to contain APEs (Cimermancic et al., 2014). This result was consistent with the finding that MGBs can exist as zoonotic or endophyte organisms (Table 1). A 14% (~26 kb) gene similarity was found between the O-antigen biosynthetic BGC from *P. aeruginosa* and the O-antigen BGC (saccharide) (Region 1) of MPH ASIOC01. A 60% (~50 kb) gene similarity was found between the enterobactin (NRP) cluster (Region 2) of MPH ASIOC01 and the enterobactin BGC from *E. coli* K-12 MG1655. Region 1 (Supplementary Table S5) showed a good-sized projected peptide core cluster (e.g., >20Kb), indicating that this pathway might encode for new natural compounds with uncharacterized BGCs, despite its overall low similarity (e.g., < 35%) with known natural products. Region 4 (Supplementary Table S5) was uncharacterized, suggesting the possible discovery of a new natural compound (Gosse et al., 2019).

#### 3.4.8 Antimicrobial resistance genes

Using the Genome Annotation Service in PATRIC, MGB genomes were examined for genes linked to antimicrobial resistance (AMR) using k-mer-based detection (Davis et al., 2020). All MGB genomes contain a similar amount of AMR-related genes (*KatG*, *MarA*, *MarR*, *Alr*, *Ddl*, *dxr*, *EF-G*, *EF-Tu*, *folA, Dfr*, *folP*, *gyrA*, *gyrB*, *inhA*, *fabI*, *Iso-tRNA, kasA*, *MurA*, *rho*, *rpoB*, *rpoC*, *S10p*, *S12p, BcrC*, *fabV*, *AcrAB-TolC*, *AcrAD-TolC*, *AcrZ*, *EmrAB-OMF*, *EmrAB-TolC*, *MacA*, *MacB, MdfA/Cmr*, *MdtABC-OMF*, *MdtABC-TolC*, *TolC/OpmH*, *gidB*, *GdpD*, *PgsA*, *OccD6/OprQ*, *OprB*, *AcrAB-TolC*, *EmrAB-TolC*, *H-NS,* and *OxyR*) (Supplementary Table S6). The genes of MGBs associated with virulence and pathogenicity were analyzed using different databases (CARD, PATRIC, DrugBank, TTD, TCDB, PATRIC_VF, VFDB, and Victors), to avoid the limited list of identifiers and genes available in the database. The average number of genes that encoded AMR (PATRIC, 58), drug target specific genes (DrugBank, 209), virulence factors (Victors, 107), and transporters (TCDB, 328) is shown in Supplementary Table S7. It has been determined that MPH MP23^T^^6^ and MPH ASIOC01 were both susceptible to kanamycin and chloramphenicol. A comparison between MPH MP23^T^ and MPH ASIOC01 revealed that the latter was resistant to ampicillin and sensitive to streptomycin. It is likely that resistance of MPH to antibiotics developed as a result of HGT, which allowed it to acquire genetic material from a shared environmental population (Lee et al., 2023b).

### 3.5 Cross-examining the MGB 16S rDNA database for species delimitation

In light of the hypothesis that MGBs had a very high 16S rDNA ANI similarity, we have carefully cross-examined the MGB 16S rDNA data deposited by the other researchers. The partial 16S rDNA sequences of MBG isolates were validated using the ANI similarity matrix (Figure 4) and reconfirmed by a BLAST search. PCA analysis of 64 aligned 16S rDNA sequences was performed; yet, no clear species clustering patterns were observed (Supplementary Figure S10). Among them, MGB sp. strain NCCP-463 (GenBank accession no. LC488948.1, 1,393 bp) and MGB sp. strain C_62_A_009 (GenBank accession no. LC655611.1, 512 bp) shared low 16S rDNA similarity, with the highest similarity among other MGBs was 79.09 and 93.44%, respectively. The identified strains were determined not to belong to MGB and were likely attributed to *Rhodococcus* sp. and *Escherichia* sp. with ANI similarity of 98.92 and 98.84%, respectively.

The MGB species, including MPH strain 11 (which has a similarity of only 95.74% with MPH MP23^T^), MYI MS2.4 (which has similarities of 98.38 and 98.26% with MYI SaN21-3 and TULL-A^T^, respectively), and MPL BCRP5 (which has a similarity of 97.66% with MPL MSSRF40^T^), were found to have a low homology in their 16S rDNA compared with the type strains. Therefore, further investigation is necessary (see Figure 4). Based on the results of a BLAST search, it can be inferred that the MPH strain 11 is likely associated with the *Erwiniaceae* bacterium, as indicated by a high similarity of 97.60%. Consequently, more investigation is warranted to examine its evolutionary ancestry in greater depth.

In contrast, it is highly probable that *Mangrovibacter* sp. isolate QUEBA02 (with a partial 16S rDNA sequence of 1,438 bps) and isolate QUEBA03 (with a partial 16S rDNA sequence of 1,443 bps) can be categorized as MPH isolates, as they exhibit a complete match in terms of 16S rDNA sequence with MPH MP23^T^ (as shown in Figure 4). The length of the 16S rDNA for these strains exceeds 1,000 base pairs, therefore providing a relatively persuasive basis for our research. A comparable approach can be employed to determine the species delimitation of other MGB isolates, including MGB sp. strain 1A (which has 100% similarity with MPH MP23^T^), MGB sp. MSSRF N44 (which shares 100% similarity with MPL strain MSSRF N80), and MGB sp. strain Eve2-HG-22 (which also shares 100% similarity with MPL strain MSSRF N80). It is noteworthy that the MPL strain isolates BCRP5, MGB sp. MSSRF N87, and MGB sp. Arv-29-1.1a had a 97.01, 96.88, and 98.02% similarity, respectively, in terms of their 16S rDNA sequence when compared with MPH TULL-A^T^, MGB sp. P4, and MGB sp. strain Eve2-HG-11. However, despite the relatively low ANI %, the BLAST search outcomes revealed that the closest related species to these isolates are still MGBs, implying the possibility that they could represent previously unidentified new species within the MGB genus.

In addition, we suggest that NCBI entries presenting a high degree of similarity in their 16S rDNA ANI (≥98%) to known MGBs, such as Endophytic bacterium SV706, SV708, SV717, SV812, *Enterobacteriaceae* bacterium JW72.7a, Bacterium N25, *Klebsiella* sp. P23, and *Salmonella* sp. ZZ-4 (as shown in Supplementary Table S1B and Figure 4), should be reclassified as MGBs. The genus *Salmonella* sp. is the closest genus in association with MGB, exhibiting a 16S rDNA ANI similarity of approximately 97.3%. It is justifiable to consider such isolates as MGB if they demonstrate a 16S rDNA ANI similarity of >98% with other MGB strains, with the exceptional cases of MPL isolate BCRP5, MGB sp. MSSRF N87, and MGB sp. Arv-29-1.1a, as previously mentioned. It is strongly recommended to employ *trpB*-gene-targeted or *pepN*-gene-targeted colony PCR, in conjunction with Sanger sequencing, to validate the identification of these strains further. Additional biochemical characterizations, such as fatty acid methyl ester (FAME) analysis and API testing, are advisable for any MGB isolate that has a distinct *trpB* or *pepN* gene in comparison to the reference strains. This outcome indicates the potential existence of a novel MGB species.

Interestingly, it was observed that the 16S rDNA sequences of MYI TULL-A^T^ depicted a higher degree of similarity to MPH (99.73%), but the 16S rDNA sequences of MYI SaN21-3 showed a higher degree of similarity to MPL (99.92%). Based on the analysis of the *groL* (KM435308.1), *rpoB* (KM435306.1), and *gryB* (KM435307.1) housekeeping genes from MYI TULL-A^T^ (Zhang et al., 2015), we found that this specific MGB strain exhibited a closer relationship with MPH, while MYI SaN21-3 displayed a stronger association with MPL (Supplementary Figure S11). Finally, according to Bayesian phylogenetic inference conducted using multi-rate PTP, it was determined that strain TULL-A^T^ and strain SaN21-3 represented a closer relationship with MPH and MPL, respectively. This finding suggests that these strains might have originated from two separate ancestral clades, as shown in Supplementary Figures S12, S13. Given the observed discrepancy, it is crucial to investigate and determine whether MYI strain TULL-A^T^ and strain SaN21-3 belong to the same taxonomic species. Additionally, the Bayesian inference of phylogeny demonstrated a strong ancestral connection between MPH ASIOC01 and MPH MP23^T^, and MGB sp. MFB070 and MPL, respectively. This outcome aligns with our previous hypothesis derived from the examination of housekeeping genes’ ANI similarities (Figure 2), OGRIs index (Figure 5C), PCA analysis of *trpB* sequence (Supplementary Figure S6B), and PCA analysis of pan-genome (Figure 6C).

### 3.6 Bioremediation prowess of MGBs

**Figure 10:**
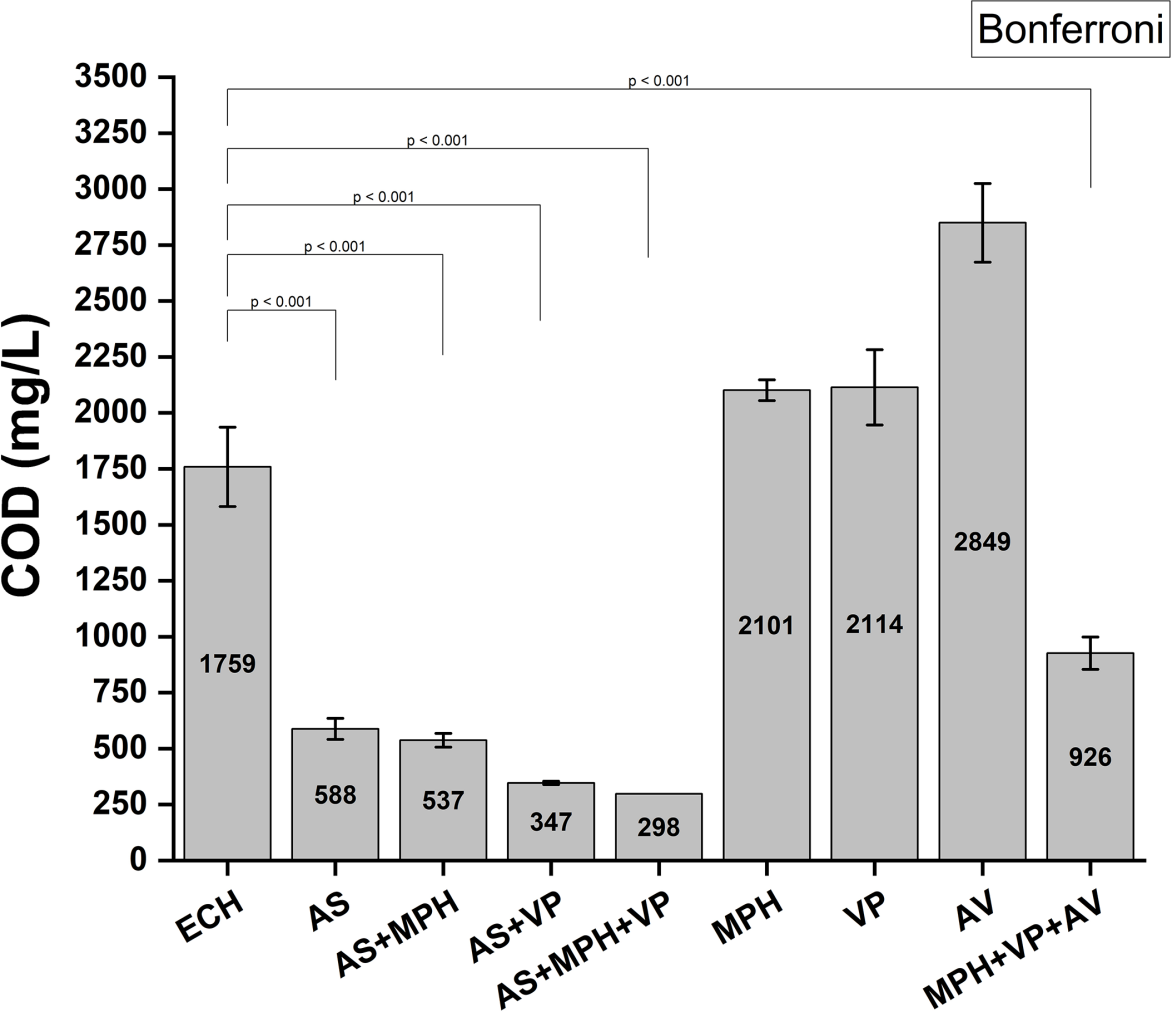
COD reduction by AS and MPH ASIOC01. ECH, wastewater; AS, activated sludge; MPH, MPH ASIOC01; VP, *Vibrio proteolyticus* strain B610AS [unpublished in-house isolate from ECH AS); AV, *Acinetobacter venetianus* RAG-1 (American Type Cultural Collection (ATCC 31012) or Bioresource Collection Research Center, Hsinchu, (BCRC 14357)].

Figure 10 illustrates the demonstrated capacity of AS and MPH ASIOC01 to effectively reduce the COD in wastewater. Our study demonstrated that AS is highly effective in the removal of approximately 67% of COD from MBR wastewater consisting of the pollutants from the wastes accumulated from ECH and AN manufacture in the laboratory incubation. By introducing selected bacteria into the sludge, an additional decrease of approximately 10% in COD was observed. The present result aligns with the findings reported by Li et al. (2019), which indicate a negative correlation between COD and MGB population during the dominance stage of AGS reactors (Li et al., 2019). The release of organic compounds during the stationary growth phase of microorganisms may lead to an elevation in COD, which can result in cell mortality or impede the formation of bacterial communities (Lee et al., 2019). It is noteworthy that a significant reduction in COD was observed when the wastewater underwent treatment using a mixed culture consisting of MPH ASIOC01, *V. proteolyticus* strain B610AS, and *A. venetianus* RAG-1. Co-culture systems provide symbiotic and synergistic advantages in the removal of nutrients from wastewater, hence addressing the challenge of elevated COD associated with pure culture approaches (Mujtaba et al., 2017).

The degradation efficacy of MPH ASIOC01 against xenobiotics present in wastewater is documented and presented in Table 4. AS has demonstrated a high degree of efficacy in the removal of organic contaminants from wastewater. Remarkably, the pure culture of MPH ASIOC01 exhibits the ability to eliminate over 60% of 1,2-propanediol, 3-chlorobutanedioyl dichloride, 2-(2-Methyl-[1,3]dithiolan-2-ylmethyl)-tetrahydro-pyran-3,4,5-triol, 2-(cyclohexyloxy)ethanol, glycerol, and trimethylolpropane present in the wastewater following a 48-h bioremediation treatment. These findings serve as a valuable addition to the aforementioned experiment on reducing COD. The enhancement of organic pollutant degradation in wastewater might potentially be achieved with more efficiency through the utilization of a microbial consortium system that is supplemented with MGBs (Chen et al., 2014; Chan et al., 2022). The bioremediation potential of MPH ASIOC01 is likely ascribed to the proteins expressed by distinct genes within the xenobiotic biodegradation pathway. The utilization of a biofilm-embedded bacterial population for the concurrent bioremediation of heavy metal and organic contaminants exhibits significant promise as a technology that is both environmentally sustainable and economically viable. However, this approach requires further scrutiny and inquiry to fully grasp its effectiveness and applicability (Ajiboye et al., 2021).

**Table 4:** The percentage of organic pollutants eliminated with the treatment either by AS or MPH ASIOC01.

Chemical pollutants	Retention time (min)	AS	MPH ASIOC01	Derivatization
1,2-Propanediol, 3-chloro	11.89	100.0 ± 0.0%	74.9 ± 22.9%	N/A
Butanedioyl dichloride	19.82	87.6 ± 7.1%	73.1 ± 23.3%	N/A
Propane,1,1′-oxybis [2,3-dichloro]	28.54	96.0 ± 1.9%	32.2 ± 23.1%	N/A
Succinic acid, monochloride, 3-hexyl ester	40.60	52.1 ± 5.4%	27.5 ± 28.7%	N/A
3-Chloro-1,2-propanediol, 2TMS derivative	21.24	95.3 ± 4.8%	85.8 ± 11.1%	BSTFA:TMCS
2-(2-Methyl-[1,3]dithiolan-2-ylmethyl)-tetrahydro-pyran-3,4,5-triol, 3TMS derivative	24.21	88.8 ± 7.2%	81.6 ± 13.1%	BSTFA:TMCS
2-(Cyclohexyloxy)ethanol, TMS derivative	25.14	80.3 ± 11.2%	77.9 ± 15.9%	BSTFA:TMCS
Glycerol, 3TMS derivative	33.54	94.4 ± 2.3%	61.2 ± 28.8%	BSTFA:TMCS
Trimethylolpropane, 3TMS derivative	42.51	57.3 ± 3.8%	63.1 ± 27.4%	BSTFA:TMCS
Glycerol monostearate, 2TMS derivative	55.35	39.4 ± 25.1%	34.4 ± 46.1%	BSTFA:TMCS
2-Chlorocyclohexanol, TMS derivative	20.97	63.6 ± 31.5%	57.6 ± 36.7%	BSTFA:TMCS

N/A, Not applicable.

## 4 Conclusion

Despite its relatively recent discovery, MGBs are widespread in nature, with new strains continuously being identified in various habitats. Based on the available data, MGBs can exist not only in a free-living form found in AS and geothermal lakes but also in an endophytic form identified on the leaves and roots of plants, as well as in a zoonotic form in the gastrointestinal tracts of insects and seaborne organisms. It is notable that MGBs are resilient to high-salt conditions and can often be found in mangroves and coastal areas. Multiple methods have succeeded in isolating pure cultures of MGBs. However, by taking advantage of its high salt-tolerance, nitrogen-fixing, and phosphate-solubilizing traits, increasing the NaCl concentration to over 6% or utilizing a nitrogen-depleted or NBRIP medium could likely facilitate the isolation of MGBs.

A new strain of MPH, MPH ASIOC01, was isolated from MBR AN-rich sludges enriched with 1,3-DCP and 3-MCPD as carbon sources. Due to the challenges in taxonomically distinguishing MGB strains based on their highly similar 16S rDNA sequences, the housekeeping gene *trpB* has been established as a valuable marker for efficiently delineating MPH, MYI, and MPL in a rapid and cost-effective manner. To gain insights into the ecological functions and evolutionary significance of MGBs, we sequenced the genome of MPH ASIOC01 utilizing PacBio SMRT HiFi sequencing and successfully obtained the complete genome as a circularized single contig. The result represents the first MGB genome assembled using long-read sequencing technology. Pan-genome analysis of the 5 MGB genomes revealed that MPH ASIOC01 possesses the most abundant strain-specific genes, highlighting its distinct genetic repertoire. This phenomenon could potentially be attributed to HGT, as supported by the enriched [X] category in COG and the HGT events identified in the PROKSEE analysis. These HGT events might contribute to the unique antibiotic-resistant phenotype carried by MPH ASIOC01.

Genes involved in glycerol degradation, nitrogen fixation, and phosphate solubilization pathways were found in all five MGB genomes. Potential genes for chromium reduction, Azo dye remediation and cellulose degradation, and synthesis were also present in its genomes. Our research has provided experimental evidence that MPH ASIOC01 is effective in eliminating organic pollutants present in wastewater. Based on empirical evidence, it is posited that MGBs could potentially play a significant role in facilitating xenobiotics bioremediation processes. The main objective of this study is to bridge the current information gap and improve our understanding of the ecological role of MGBs in diverse habitats.

## Data availability statement

The author selected the following statement: the datasets presented in this study can be found in online repositories. The names of the repository/repositories and accession number(s) can be found in the article/Supplementary material.

## Author contributions

HC: Conceptualization, Data curation, Formal analysis, Investigation, Methodology, Software, Validation, Visualization, Writing – original draft, Writing – review & editing. NRV: Conceptualization, Data curation, Formal analysis, Investigation, Methodology, Software, Validation, Visualization, Writing – original draft, Writing – review & editing. Z-HL: Conceptualization, Data curation, Formal analysis, Investigation, Methodology, Software, Validation, Visualization, Writing – original draft, Writing – review & editing. W-YC: Formal analysis, Methodology, Writing – review & editing. Z-HZ: Formal analysis, Methodology, Writing – review & editing. SA: Methodology, Writing – review & editing. C-YL: Methodology, Writing – review & editing. SS-FY: Conceptualization, Funding acquisition, Project administration, Resources, Supervision, Validation, Visualization, Writing – original draft, Writing – review & editing, Investigation.

## Footnotes

^1^ https://digitalinsights.qiagen.com/

^2^ https://www.patricbrc.org/

^3^ https://pubmlst.org/

^4^ https://www.ebi.ac.uk/Tools/msa/clustalo/

^5^ https://itol.embl.de/

^6^ https://bacdive.dsmz.de/strain/140470
