# Supplementary material for: Isolation, molecular identification, and genomic analysis of Mangrovibacter phragmitis strain ASIOC01 from activated sludge harboring the bioremediation prowess of glycerol and organic pollutants in high-salinity
Source: Front Microbiol. 2024 Jun 25;15:1415723. doi: 10.3389/fmicb.2024.1415723 (PMC11231211; doi:10.3389/fmicb.2024.1415723)
Supplement: Supplementary file 1 [file Data_Sheet_1.PDF]

SUPPLEMENTARY MATERIAL

Isolation, molecular identification, and genomic analysis of *Mangrovibacter phragmitis* strain ASIOC01 from activated sludge harboring the bioremediation prowess of glycerol- and organic pollutants in high-salinity

Hong Soon Chin[1,2,3] Narendrakumar Ravi Varadharajulu[1,4,5], Zhi-Han Lin[1,2,6], Wen-Yu Chen[1], Zong-Han Zhang[1,7], Sankar Arumugam[1], Ching-Yen Lai[1] and Steve S.-F. Yu[1,2,4,7*]

[1]Institute of Chemistry, Academia Sinica, Taipei, Taiwan

[2]Chemical Biology and Molecular Biophysics Program, Taiwan International Graduate Program, Academia Sinica, Taipei, Taiwan

[3]Institute of Bioinformatics and Structural Biology, National Tsing Hua University, Hsinchu, Taiwan

[4]Molecular Science and Technology Program, Taiwan International Graduate Program, Academia Sinica, Taipei, Taiwan

[5]Department of Chemistry, National Tsing Hua University, Hsinchu, Taiwan

[6]Institute of Biochemical Sciences, National Taiwan University, Taipei, Taiwan

[7]Ph.D. Program in Microbial Genomics, National Chung Hsing University, Taichung City, Taiwan

*** Correspondence:**

Steve S.-F. Yu

sfyu@gate.sinica.edu.tw

SUPPLEMENTARY DOCUMENTS

Supplementary Document 1: V3-V4 short-read (V3V4) metagenomics

The study of V3-V4 short-read metagenomics were undertaken by Genomics. Inc., Taipei. The extracted DNA was then quantitated and adjusted to 5 ng/ul using NanoDrop 2000 Spectrophotometers (Thermo Scientific, USA). For the 16S rRNA gene sequencing, V3-V4 region was amplified by a specific primer set (341F: 5'-CCTACGGGNGGCWGCAG-3', 806R: 5'-GACTACHVGGGTATCTAATCC-3') according to the 16S Metagenomic Sequencing Library Preparation procedure (Part:.15044223, Illumina, USA). In brief, 12.5 ng of gDNA was used for the PCR reaction carried out with KAPA HiFi HotStart ReadyMix (Cat. no. KK2602, Roche, Switzerland) under the PCR condition: 95°C for 3 minutes; 25 cycles of: 95°C for 30 seconds, 55°C for 30 seconds, 72°C for 30 seconds; 72°C for 5 minutes and hold at 4°C. The PCR products were monitored on 1.5% agarose gel. Samples with bright main strips around 500 bp were chosen and purified by using the AMPure XP beads (Beckman Coulter, USA) for the following library preparation. The Sequencing library was prepared according to the 16S Metagenomic Sequencing Library Preparation procedure (Illumina, USA). In brief, a secondary PCR was performed by using the PCR amplicons at the 16S rRNA V3-V4 region and Nextera XT Index Kit with dual indices and Illumina sequencing adapters (Illumina, USA). The indexed PCR product quality was assessed on the Qubit 4.0 Fluorometer (Thermo Scientific, USA) and Qsep100 system (BiOptic, Taiwan). An equal amount of the indexed PCR product was mixed to generate the sequencing library. Finally, the library was sequenced on an Illumina MiSeq platform, and paired 300-bp reads were generated. De-multiplexing was carried out based on barcode identification. The sequencing outcomes were then processed (adaptor trimming) and analyzed (microbial community composition) using OTU clustering function under Amplicon-based analysis workflow in the Microbial Genomics Module of QIAGEN CLC Genomics Workbench v22 (https://digitalinsights.qiagen.com/). 16s rRNA were aligned and grouped into operational taxonomic units (OTUs) with 97% similarity. The NCBI DB (BioProject accession no. PRJNA33175) was used as the metataxonomic classifiers in this study.

SUPPLEMENTARY DOCUMENTS

Supplementary Document 2: Whole genome sequencing (WGS) and assembly

The genome study of MPH was conducted by Genomics, Inc. Taiwan, and sequenced by a shotgun sequencing method using the PacBio Sequel platform (PacBio, USA). The library construction was performed according to the manufacturer's instruction provided by SMRTbell Express template Prep Kit 2.0 (P/N PB100-938-900, PacBio, USA). The genomic DNA was sheared by using Covaris g-TUBE (Covaris, USA) and purified via AMPure PB beads (Beckman Coulter, USA). The A-tailed inserts were then ligated with T-tailed adaptors. Small insert SMRTbell templates were removed using BluePippin System (Sage Science, USA). The final SMRTbells were annealed with sequencing primers and bound to the proprietary polymerase using the Sequel Binding Kit v3.0 (P/N PB100-626-600) to form the binding complex. After dilution, the library was loaded onto the instrument with Sequel Sequencing Kit 3.0 (P/N PB101-597-900, PacBio, USA) and SMRT Cell 1M v3 Tray (P/N PB101-531-000 or PB101-531-001, PacBio USA) for sequencing. A primary filtering analysis was performed with the Sequel instrument, including signal processing of the movie, base calling of the traces and pulses, quality assessment of the base calls, trimming of the sequences to the high-quality (HQ) regions, identifying adapter, controlling sequence reads, assigning read scores, and generating the subread data in a BAM file. The hifi reads were generated by subreads with 3 passes and 0.99 predicted accuracy. They were going to be assembled by hifiasm v0.8 (Cheng et al., 2021). The longer subreads were assembled by "flye" and polished by "Arrow algorithm from GenomicConsensus v2.3.3. package "(PacBio, USA) and circularized by "Circlator v1.5.5" (Hunt et al., 2015). The secondary analysis was performed using the SMRT analysis pipeline version 8.0, which includes filtering of data that meets a desired criterion, comparison of reads to a reference or between each other for mapping and variant calling, consensus sequence determination, alignment, and assembly (de novo or reference-based) and quality evaluations for a sequencing run. SSPACE-LongRead v1.1 (Boetzer and Pirovano, 2014) and PBJelly v15.8.24 software (English et al., 2012) were used for closing gaps in genome assemblies reads. Lastly, QUAST v4.6.3 (Gurevich et al., 2013) was used to evaluate the quality of the assembled genome. After completing the genome assembly, Prokka v1.12(Seemann, 2014) was used for gene prediction. An overall PacBio de-novo assembly experiment workflow for MPH ASIOC01 was shown in Supplementary Figure 1.

References:

Boetzer, M., and Pirovano, W. (2014). SSPACE-LongRead: scaffolding bacterial draft genomes using long read sequence information. BMC Bioinformatics 15, 211.

Cheng, H., Concepcion, G.T., Feng, X., Zhang, H., and Li, H. (2021). Haplotype-resolved de novo assembly using phased assembly graphs with hifiasm. Nat Methods 18, 170-175.

English, A.C., Richards, S., Han, Y., Wang, M., Vee, V., Qu, J., Qin, X., Muzny, D.M., Reid, J.G., Worley, K.C., and Gibbs, R.A. (2012). Mind the gap: upgrading genomes with Pacific Biosciences RS long-read sequencing technology. PLoS One 7, e47768.

Gurevich, A., Saveliev, V., Vyahhi, N., and Tesler, G. (2013). QUAST: quality assessment tool for genome assemblies. Bioinformatics 29, 1072-1075.

Hunt, M., Silva, N.D., Otto, T.D., Parkhill, J., Keane, J.A., and Harris, S.R. (2015). Circlator: automated circularization of genome assemblies using long sequencing reads. Genome Biol 16, 294.

Seemann, T. (2014). Prokka: rapid prokaryotic genome annotation. Bioinformatics 30, 2068-2069.

Supplementary Figures

A

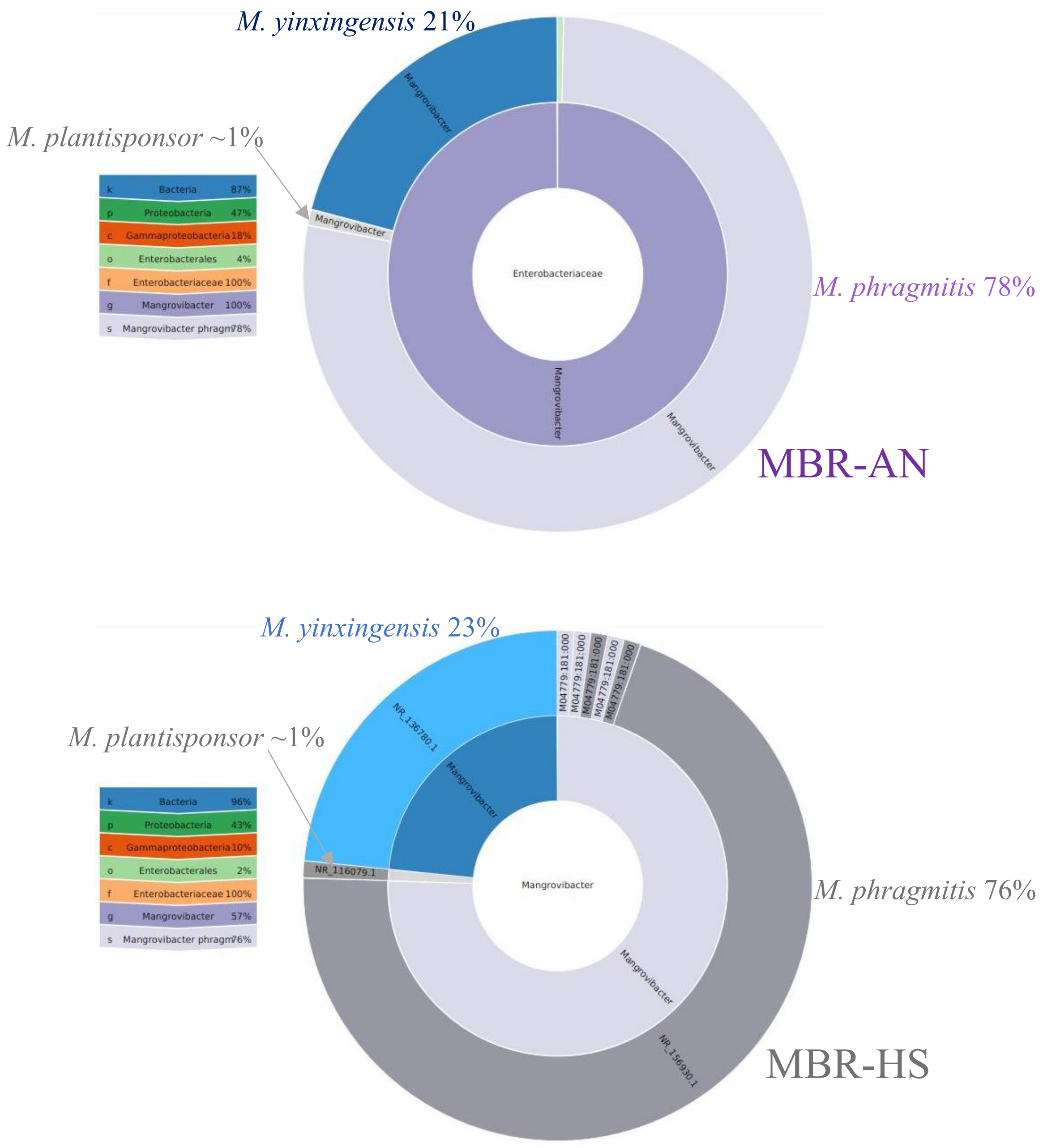

FIGURE S1

MPH and MYI distribution in (A) MBR-AN and (B) MBR-HS activated sludge, identified by NCBI DB (BioProject accession no. PRJNA33175) using Qiagen CLC Genomics Workbench v24 (https://digitalinsights.qiagen.com/). MBR: Membrane bioreactor; AN: Acrylonitrile; HS: High-salt.

Supplementary Figures

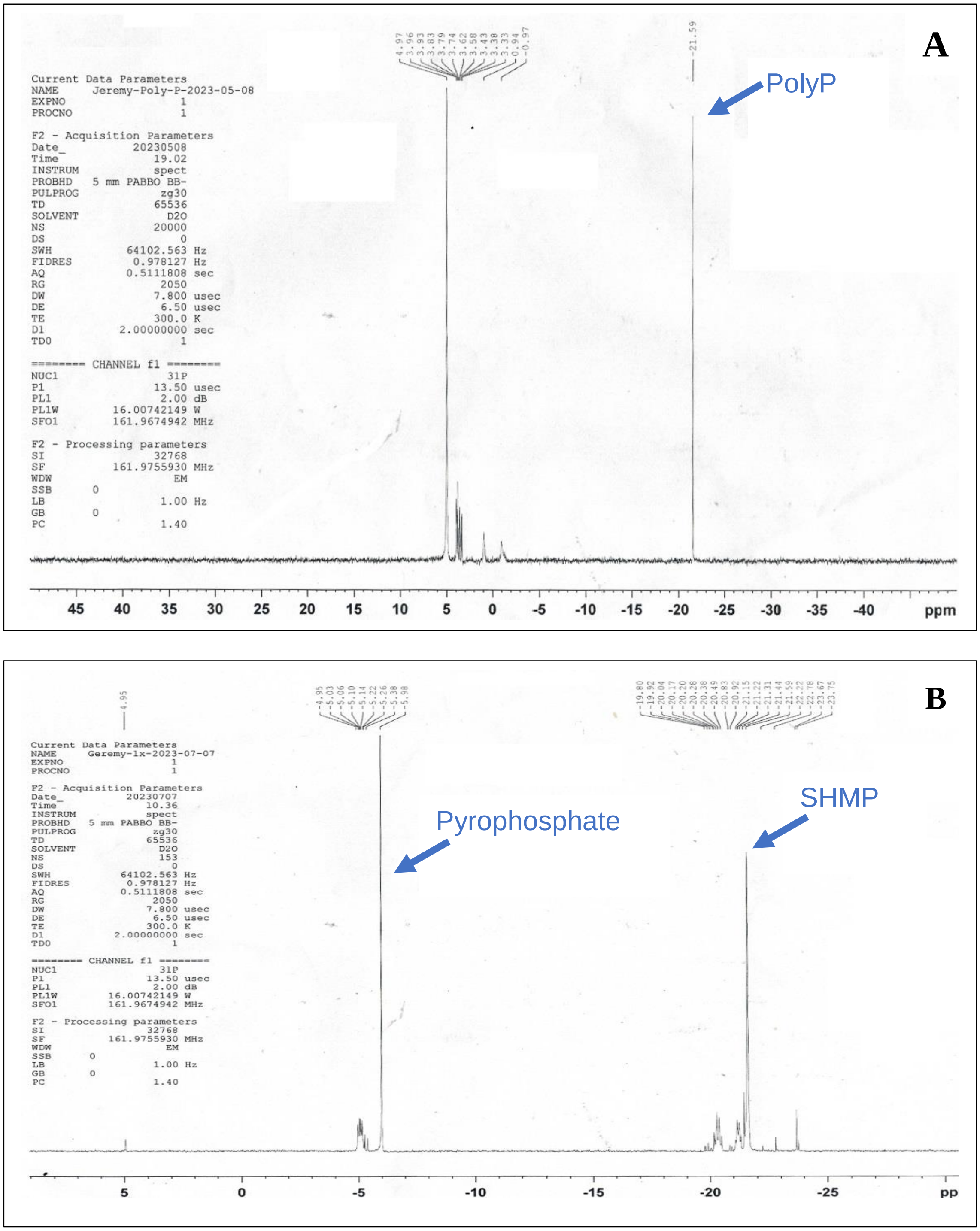

FIGURE S2

^{31}P NMR spectra of a variety of phosphate derivatives. (A) ^{31}P NMR spectrum of polyP extracted from the MPH ASIOC01 cell lysate. (B) ^{31}P NMR spectrum of pyrophosphate (Aldrich-P8010) and sodium hexametaphosphate (SHMP) polyP standard (Supelco Supelco-106529, $(NaPO_3)_n$).

Supplementary Figures

	1	2	3	4	5	6	7	8	9	10	11
1 GS_WGS_NBRC108236		99.93	99.78	99.79	99.92	97.20	99.86	99.79	100.00	98.21	99.92
2 GS_SPR2			99.71	99.72	99.85	97.20	99.79	99.72	99.90	98.14	99.84
3 GS_E2_18				99.71	99.77	98.50	99.78	99.86	99.70	98.06	99.76
4 GS_LW_XY5					99.92	98.43	99.79	99.86	99.90	98.21	99.92
5 GS_SBUG_67						99.62	99.92	99.92	100.00	98.39	99.84
6 GS_OH_4							98.22	98.36	99.40	97.21	99.84
7 GC_Chol3								99.86	100.00	98.21	99.92
8 GC_GEEL									100.00	98.21	99.92
9 GC_Sl2										97.87	99.90
10 GC_QB8											98.31
11 GC_V93DM											

List of GS and GC strains and its 16S rRNA analyzed in this study.

No	Species	Strain	Accession no.	Data type	Length (bp)
1	GS	NBRC 108236	Genome (GCF_000333035.1)	16S rRNA	1521
2	GS	SPR2	NR_025505.1	16S rRNA	1499
3	GS	E2-18	OQ753133.1	16S rRNA	1392
4	GS	LW-XY5	MN880097.1	16S rRNA	1406
5	GS	SBUG 67	FR745417.1	16S rRNA	1306
6	GS	oh-4	EU862318.1	16S rRNA	1394
7	GC	Chol-3	NR_044445.1	16S rRNA	1445
8	GC	GEEL-03	MW486646.1	16S rRNA	1420
9	GC	Sl2	MT632490.1	16S rRNA	1042
10	GC	QB8.2	KU597103.1	16S rRNA	1343
11	GC	V93DM	JN400330.1	16S rRNA	1242

FIGURE S3

The 16S rRNA average nucleotide identity (ANI) matrix of *Gordonia sihwensis* (GS) and *G. cholesterolivorans* (GC) generated using Clustal Omega v1.2.4 Multiple Sequence Alignment (https://www.ebi.ac.uk/Tools/msa/clustalo/). The list of various *Gordonia* strains used in this study was tabulated.

Supplementary Figures

ANI Index (%)

	MPH vs MYI	MPH vs MPL	MYI vs MPL		MFB070 vs MPH	MFB070 vs MYI	MFB070 vs MPL	MP23 vs ASIOC
dnaA	94.00	94.79	94.22	dnaA	95.30	94.36	98.34	99.70
pyrG	94.93	96.64	94.57	pyrG	97.01	95.05	98.90	99.88
rpoB	96.97	97.79	98.21	rpoB	97.89	98.16	99.65	99.80
groL	95.08	95.99	95.51	groL	96.05	95.75	99.39	99.94
recA	93.90	97.56	93.90	recA	97.28	93.43	99.34	99.72
clpX	91.45	93.96	92.78	clpX	93.88	92.71	99.53	99.76
carB	92.71	94.05	93.80	carB	94.11	93.86	99.41	99.91
MurC	90.11	93.50	92.14	MurC	93.63	92.21	99.25	99.80
pepN	89.32	93.19	89.93	pepN	93.07	89.97	99.12	99.00
pheS	93.60	98.48	93.70	pheS	98.48	93.90	99.19	96.75
gyrB	94.46	95.29	95.33	gyrB	95.04	95.82	97.68	99.46
rpoD	95.60	98.21	96.58	rpoD	98.21	96.47	99.67	99.95
dnaK	94.21	94.89	96.35	dnaK	95.04	96.45	98.64	99.69
trpB	88.61	94.81	89.53	trpB	94.14	89.11	98.74	99.66

No	Gene	
1	dnaK	Chromosomal replication initiator protein DnaA
2	pyrG	CTP synthase
3	rpoB	DNA-directed RNA polymerase subunit beta
4	groL	Chaperonin GroEL
5	recA	Protein RecA
6	clpX	ATP-dependent Clp protease ATP-binding subunit ClpX
7	carB	Carbamoyl-phosphate synthase large chain
8	murC	UDP-N-acetylmuramate-L-alanine ligase
9	pepN	Aminopeptidase N
10	pheS	Phenylalanine-tRNA ligase alpha subunit
11	gyrB	DNA gyrase B
12	rpoD	RNA polymerase sigma factor RpoD
13	danK	Chaperone protein DnaK
14	trpB	Tryptophan synthase beta chain

ANI index (%) High similarity Low similarity

dnaA	MPH	MYI	MPL	MFB070	
MPH_MP23_Genome		94.00	94.79	95.30	MPH
MYI_SaN23-3_Genome			94.22	94.36	MYI
MPL_DSM19579_Genome				98.34	MPL
Sp_MFB070_Genome					MFB070

pyrG	MPH	MYI	MPL	MFB070	
MPH_MP23_Genome		94.93	96.64	97.01	MPH
MYI_SaN23-3_Genome			94.57	95.05	MYI
MPL_DSM19579_Genome				98.90	MPL
Sp_MFB070_Genome					MFB070

rpoB	MPH	MYI	MPL	MFB070	
MPH_MP23_Genome		96.97	97.79	97.89	MPH
MYI_SaN23-3_Genome			98.21	98.16	MYI
MPL_DSM19579_Genome				99.65	MPL
Sp_MFB070_Genome					MFB070

groL	MPH	MYI	MPL	MFB070	
MPH_MP23_Genome		95.08	95.99	96.05	MPH
MYI_SaN23-3_Genome			95.51	95.75	MYI
MPL_DSM19579_Genome				99.39	MPL
Sp_MFB070_Genome					MFB070

recA	MPH	MYI	MPL	MFB070	
MPH_MP23_Genome		93.90	97.56	97.28	MPH
MYI_SaN23-3_Genome			93.90	93.43	MYI
MPL_DSM19579_Genome				99.34	MPL
Sp_MFB070_Genome					MFB070

clpX	MPH	MYI	MPL	MFB070	
MPH_MP23_Genome		91.45	93.96	93.88	MPH
MYI_SaN23-3_Genome			92.78	92.71	MYI
MPL_DSM19579_Genome				99.53	MPL
Sp_MFB070_Genome					MFB070

carB	MPH	MYI	MPL	MFB070	
MPH_MP23_Genome		92.71	94.05	94.11	MPH
MYI_SaN23-3_Genome			93.80	93.86	MYI
MPL_DSM19579_Genome				99.41	MPL
Sp_MFB070_Genome					MFB070

murC	MPH	MYI	MPL	MFB070	
MPH_MP23_Genome		90.11	93.50	93.63	MPH
MYI_SaN23-3_Genome			92.14	92.21	MYI
MPL_DSM19579_Genome				99.25	MPL
Sp_MFB070_Genome					MFB070

pepN	MPH	MYI	MPL	MFB070	
MPH_MP23_Genome		89.32	93.19	93.07	MPH
MYI_SaN23-3_Genome			89.93	89.97	MYI
MPL_DSM19579_Genome				99.12	MPL
Sp_MFB070_Genome					MFB070

pheS	MPH	MYI	MPL	MFB070	
MPH_MP23_Genome		93.60	98.48	98.48	MPH
MYI_SaN23-3_Genome			93.70	93.90	MYI
MPL_DSM19579_Genome				99.19	MPL
Sp_MFB070_Genome					MFB070

gyrB	MPH	MYI	MPL	MFB070	
MPH_MP23_Genome		94.46	95.29	95.04	MPH
MYI_SaN23-3_Genome			95.33	95.82	MYI
MPL_DSM19579_Genome				97.68	MPL
Sp_MFB070_Genome					MFB070

rpoD	MPH	MYI	MPL	MFB070	
MPH_MP23_Genome		95.60	98.21	98.21	MPH
MYI_SaN23-3_Genome			96.58	96.47	MYI
MPL_DSM19579_Genome				99.67	MPL
Sp_MFB070_Genome					MFB070

dnaK	MPH	MYI	MPL	MFB070	
MPH_MP23_Genome		94.21	94.89	95.04	MPH
MYI_SaN23-3_Genome			96.35	96.45	MYI
MPL_DSM19579_Genome				98.64	MPL
Sp_MFB070_Genome					MFB070

trpB	MPH	MYI	MPL	MFB070	
MPH_MP23_Genome		88.61	94.81	94.14	MPH
MYI_SaN23-3_Genome			89.53	89.11	MYI
MPL_DSM19579_Genome				98.74	MPL
Sp_MFB070_Genome					MFB070

MFB070 vs MPL average 99.06

FIGURE S4

The housekeeping genes' average nucleotide identity (ANI) similarity index among MGBs. Clustal Omega v1.2.4 Multiple Sequence Alignment (https://www.ebi.ac.uk/Tools/msa/clustalo/) was used to generate the percent identity matrix.

Supplementary Figures

```
                                               Forward primer
MPH_ASIOC_trpB      ATGACAACTTTATTAAACCCGTATTTTGGTGAATTCGGCGGAATGTACGTTCCGCAAATA 60
MP23_trpB           ATGACAACTTTATTAAACCCGTATTTTGGTGAATTCGGCGGAATGTACGTTCCGCAAATA 60
MFB070_trpB         ATGACGACTTTATTAAACCCGTATTTTGGTGAATTCGGCGGAATGTATGTTCCGCAAATA 60
MYI_trpB            ATGACAACTTTATTAAACCCGTATTTTGGTGAATTCGGCGGAATGTATGTTCCGCAAATA 60
MPL_trpB            ATGACGACTTTATTAAACCCGTATTTTGGTGAATTCGGCGGAATGTATGTTCCGCAAATA 60
                    ***** ***************************************** ************

MPH_ASIOC_trpB      CTGATGCCAGCATTACGCCAGCTTGAAGAAGCGTTTGTCAGTGCGCAAAGTGACCCTGAG 120
MP23_trpB           CTGATGCCAGCATTACGCCAGCTTGAAGAAGCGTTTGTCAGTGCGCAAAGTGACCCTGAG 120
MFB070_trpB         CTGATGCCAGCATTACGCCAGCTTGAAGAAGCGTTTGTCAGTGCGCAAAGCGACCCTGAG 120
MYI_trpB            CTGATGCCAGCATTACGCCAGCTTGAAGAAGCGTTTGTCAGTGCACAAAGTGACCCTGAG 120
MPL_trpB            CTGATGCCAGCATTACGCCAGCTTGAAGAAGCGTTTGTCAGTGCACAAAGTGACCCTGAG 120
                    ******************************************** ***** *********

MPH_ASIOC_trpB      TTCCAGGCGCAGTTTACTGATTTGCTGAAAAATTATGCCGGGCGCCCCACTGCCCTTACC 180
MP23_trpB           TTCCAGGCGCAGTTTACTGATTTGCTGAAAAATTATGCCGGGCGCCCCACTGCCCTTACC 180
MFB070_trpB         TTCCAGGCGCAGTTCACTGATTTGCTGAAAAATTATGCCGGGCGCCCCACTGCCCTTACC 180
MYI_trpB            TTCCAGGCGCAGTTTACTGATTTGCTGAAAAATTATGCTGGGCGACCGACTGCCCTTACC 180
MPL_trpB            TTCCAGGCGCAGTTCACTGATTTGCTGAAAAATTATGCCGGGCGCCCAACTGCCCTTACC 180
                    ************** *********************** ***** ** ************

MPH_ASIOC_trpB      CGCTGTCGTAATCTGACTGCGGGAACCAAAACCACTCTGTACCTTAAACGTGAAGATTTG 240
MP23_trpB           CGCTGTCGTAATCTGACTGCGGGAACCAAAACCACTCTGTACCTTAAACGTGAAGATTTG 240
MFB070_trpB         CGCTGCCGTAATCTGACTGCGGGAACCAAAACCACGCTGTACCTTAAACGTGAAGATTTG 240
MYI_trpB            CGTTGCCGTAACCTGACTGCAGGGACAAAAACCACGCTTTACCTTAAGCGTGAAGATTTG 240
MPL_trpB            CGCTGCCGTAATCTGACTGCGGGAACCAAAACCACGCTGTACCTTAAACGTGAAGATTTG 240
                    ** ** ***** ******** ** ** ******** ** ******** ************

MPH_ASIOC_trpB      CTGCATGGCGGCGCACATAAAACAAACCAGGTTCTGGGCCAGGCTCTGCTCGCCAAACGC 300
MP23_trpB           CTGCATGGCGGCGCACATAAAACAAACCAGGTTCTGGGCCAGGCTCTGCTCGCCAAACGC 300
MFB070_trpB         CTCCATGGTGGCGCACATAAAACGAACCAGGTGCTGGGCCAGGCTCTGCTTGCCAAACGC 300
MYI_trpB            CTGCATGGCGGCGCACATAAAACTAACCAGGTGCTGGGGCAAGCTTTGCTCGCCAAGCGG 300
MPL_trpB            CTGCATGGCGGCGCACATAAAACGAACCAGGTGCTGGGCCAGGCTCTGCTTGCCAAACGC 300
                    ** ***** ************** ******** ***** ** *** **** ***** ** 

MPH_ASIOC_trpB      ATGGGCAAAACCGAAATCATTGCTGAGACAGGTGCCGGGCAACACGGTGTGGCATCCGCG 360
MP23_trpB           ATGGGCAAAACCGAAATCATTGCTGAGACAGGTGCCGGGCAACACGGTGTGGCATCCGCG 360
MFB070_trpB         ATGGGCAAAACCGAAATCATTGCTGAGACCGGTGCCGGGCAACACGGTGTGGCATCCGCA 360
MYI_trpB            ATGGGGAAAACTGAAATTATCGCTGAGACTGGTGCCGGTCAACACGGGGTTGCATCCGCA 360
MPL_trpB            ATGGGCAAAACCGAAATCATTGCTGAGACCGGTGCCGGGCAACACGGTGTGGCATCCGCA 360
                    ***** ***** ***** ** ******** ******** ******** ** ******** 

MPH_ASIOC_trpB      CTCGCCAGTGCACTGCTTGGTCTGAAATGCCGAATCTATATGGGTGCAAAGGACGTTGAG 420
MP23_trpB           CTCGCCAGTGCACTGCTTGGTCTGAAATGCCGAATCTATATGGGTGCAAAGGACGTTGAG 420
MFB070_trpB         CTAGCCAGTGCACTGCTCGGGCTGAAATGCCGAATCTATATGGGTGCGAAGGACGTTGAG 420
MYI_trpB            CTTGCCAGTGCACTGCTTGGCCTGAAATGCAGAATTTACATGGGGGCAAAAGATGTTGAA 420
MPL_trpB            CTCGCCAGTGCACTGCTCGGGCTGAAATGCCGAATCTATATGGGTGCGAAGGACGTTGAG 420
                    ** ************** ** ********* **** ** ***** ** ** ** ***** 

MPH_ASIOC_trpB      CGCCAGGCTCCGAATGTTTTTCGTATGCGTTTAATGGGTGCTGAAGTTATCCCTGTCCAT 480
MP23_trpB           CGCCAGGCTCCGAATGTTTTTCGTATGCGTTTAATGGGTGCTGAAGTTATCCCTGTCCAT 480
MFB070_trpB         CGCCAGTCTCCAAACGTTTTCCGTATGCGTTTAATGGGCGCGGAAGTGATTCCTGTTCAT 480
MYI_trpB            CGTCAGTCACCTAATGTATTCCGTATGCGTTTAATGGGTGCGGAAGTCATTCCTGTTCAT 480
MPL_trpB            CGCCAGTCTCCAAACGTTTTCCGTATGCGTTTAATGGGTGCGGAAGTTATTCCTGTTCAT 480
                    ** *** * ** ** ** ** ***************** ** ***** ** ***** ***

MPH_ASIOC_trpB      TCCGGGTCATCAACCCTCAAAGATGCCTGTAACGAAGCACTGCGTGACTGGTCTGGTAGT 540
MP23_trpB           TCCGGGTCATCAACCCTCAAAGATGCCTGTAACGAAGCACTGCGTGACTGGTCTGGTAGT 540
MFB070_trpB         TCCGGGTCATCGACCCTGAAAGACGCCTGTAACGAAGCTCTGCGTGACTGGTCTGGTAGT 540
MYI_trpB            TCTGGTTCATCAACATTAAAAGATGCATGTAATGAAGCACTGCGTGACTGGTCTGGCAGT 540
MPL_trpB            TCCGGGTCATCGACCCTGAAAGACGCCTGTAACGAAGCTCTGCGTGACTGGTCTGGTAGT 540
                    ** ** ***** **  * ***** ** ***** ***** ***************** ***

MPH_ASIOC_trpB      TATGAAACCGCACACTACATGCTGGGTACTGCCGCGGGCCCTCATCCTTATCCAACTATT 600
MP23_trpB           TATGAAACCGCGCACTATATGCTGGGTACTGCCGCGGGCCCTCATCCTTATCCAACTATT 600
MFB070_trpB         TATGAAACCGCGCACTACATGCTGGGTACTGCTGCCGGTCCGCATCCTTACCCGACTATT 600
MYI_trpB            TATGAAACCGCACACTATATGCTGGGCACTGCTGCAGGTCCTCATCCTTACCCAACCATT 600
MPL_trpB            TATGAAACCGCGCACTACATGCTGGGTACTGCTGCCGGTCCGCATCCTTACCCGACTATT 600
                    *********** ***** ******** ***** ** ** ** ******** ** ** ***
```

```
MPH_ASIOC_trpB      GTCCGCGAATACCAGCGCATGATTGGCGAAGAAACCCGGGCGCAAATTCTTGAAAAAGAA 660
MP23_trpB           GTCCGCGAATACCAGCGCATGATTGGCGAAGAAACCCGGGCGCAAATTCTTGAAAAAGAA 660
MFB070_trpB         GTCCGTGAATACCAGCGTATGATTGGCGAGGAAACCCGGGCGCAAATTCTTGAGAAAGAA 660
MYI_trpB            GTCCGCGAATACCAACGTATGATTGGCGAGGAAACCCGTGCGCAAATTCTTGAGAAAGAA 660
MPL_trpB            GTCCGCGAATACCAGCGCATGATTGGCGAGGAAACCCGGGCGCAAATTCTTGAGAAAGAA 660
                    ***** ******** ** *********** ******** ************** ******

MPH_ASIOC_trpB      GGTCGCCTGCCAGATGCCGTTATCGCCTGTGTAGGTGGTGGTTCCAACGCCATTGGTATG 720
MP23_trpB           GGTCGCCTGCCAGATGCCGTTATCGCCTGTGTAGGTGGTGGTTCCAACGCCATTGGTATG 720
MFB070_trpB         GGTCGCCTGCCAGATGCCGTCATCGCCTGTGTAGGTGGAGGTTCTAATGCCATTGGTATG 720
MYI_trpB            GGTCGCCTGCCAGACGCTGTTATTGCCTGTGTGGGCGGTGGCTCTAATGCTATTGGGATG 720
MPL_trpB            GGTCGCCTGCCAGATGCCGTCATTGCCTGTGTAGGTGGTGGTTCTAACGCCATTGGTATG 720
                    ************** ** ** ** ******** ** ** ** ** ** ** ***** ***

MPH_ASIOC_trpB      TTTGCAGATTTTATTGACGAAACCAGTGTCAGCCTGATTGGTGTTGAGCCTGCCGGCCAT 780
MP23_trpB           TTTGCAGATTTTATTGACGAAACCAGTGTCAGCCTGATTGGTGTTGAGCCTGCCGGCCAT 780
MFB070_trpB         TTTGCAGATTTTATTGACGAAACCAGTGTCAGCCTGATTGGTGTTGAGCCAGCCGGCCAT 780
MYI_trpB            TTCGCAGATTTTATCGATGAAACCAGTGTCAACCTGATTGGTGTTGAACCCGCCGGGCAT 780
MPL_trpB            TTTGCAGATTTTATTGACGAAACCAGTGTCAGCCTGATTGGTGTTGAGCCAGCCGGCCAT 780
                    ** *********** ** ************* *************** ** ***** ***

MPH_ASIOC_trpB      GGTATCGAAACAGGTGAACATGGTGCGCCACTCAAACACGGGCGAGTGGGCATCTATTTC 840
MP23_trpB           GGTATCGAAACAGGTGAACATGGTGCGCCACTCAAACACGGGCGAGTGGGCATCTATTTC 840
MFB070_trpB         GGTATTGAAACCGGTGAACATGGTGCGCCACTCAAGCACGGGCGCGTGGGCATCTATTTC 840
MYI_trpB            GGCATCGAAACAGGCGAACACGGGGCACCACTCAAGCATGGGCGCGTGGGCATTTACTTC 840
MPL_trpB            GGTATTGAAACCGGTGAACATGGTGCGCCACTCAAGCACGGGCGCGTGGGCATCTATTTC 840
                    ** ** ***** ** ***** ** ** ******** ** ***** ******** ** ***

MPH_ASIOC_trpB      GGTATGAAATCACCGATGATGCAAACGGATGAAGGGCAAATTGAAGAGTCATACTCTATT 900
MP23_trpB           GGTATGAAATCACCGATGATGCAAACGGATGAAGGGCAAATTGAAGAGTCATACTCTATT 900
MFB070_trpB         GGTATGAAGTCTCCCATGATGCAAACTGATGAAGGGCAAATTGAAGAGTCATACTCTATT 900
MYI_trpB            GGTATGAAGTCCCCTATGATGCAAACCGACGAAGGGCAGATTGAAGAATCTTACTCTATC 900
MPL_trpB            GGTATGAAGTCGCCCATGATGCAAACTGATGAAGGGCAAATTGAAGAGTCATACTCTATT 900
                    ******** ** ** *********** ** ******** ******** ** ******** 

MPH_ASIOC_trpB      TCTGCCGGGCTGGACTTCCCGTCAGTTGGGCCGCAGCATGCATTTCTTAACAGCACTGGC 960
MP23_trpB           TCTGCCGGGCTGGACTTCCCGTCAGTTGGGCCGCAGCATGCATTTCTTAACAGCACTGGC 960
MFB070_trpB         TCTGCCGGGCTGGACTTCCCGTCAGTAGGGCCACAGCATGCATTCCTTAACAGCACTGGC 960
MYI_trpB            TCTGCGGGGCTGGATTTTCCTTCTGTCGGGCCACAGCATGCATTCCTTAACAGTACGGGC 960
MPL_trpB            TCTGCCGGGCTGGACTTCCCGTCAGTAGGGCCGCAGCATGCATTCCTTAACAGCACTGGC 960
                    ***** ******** ** ** ** ** ***** *********** ******** ** ***

MPH_ASIOC_trpB      CGCGCAGATTATGTCTCTATTACTGACGATGAAGCGCTGGACGCGTTTAAAGAGCTGAGC 1020
MP23_trpB           CGCGCAGATTATGTCTCTATTACTGACGATGAAGCGCTGGACGCGTTTAAAGAGCTGAGC 1020
MFB070_trpB         CGCGCAGATTATGTCTCGATCACTGACGATGAAGCGCTGGAAGCGTTTAAAGAACTGAGC 1020
MYI_trpB            CGTGCGGACTATGTCTCTATTACTGACGATGAAGCTCTTGACGCTTTTAAAGAACTGAGC 1020
MPL_trpB            CGCGCAGATTATGTCTCGATCACTGACGATGAAGCGCTGGAAGCGTTTAAAGAACTGAGC 1020
                    ** ** ** ******** ** ************** ** ** ** ******** ******

MPH_ASIOC_trpB      CGTCATGAAGGCATTATCCCGGCACTGGAATCGTCACATGCACTGGCATGGGCGCTCAAA 1080
MP23_trpB           CGTCATGAAGGCATTATCCCGGCACTGGAATCGTCACATGCACTGGCATGGGCGCTCAAA 1080
MFB070_trpB         CGCCATGAAGGCATCATCCCGGCACTGGAATCTTCCCATGCACTTGCGTGGGCGCTCAAA 1080
MYI_trpB            CGCCATGAAGGCATCATTCCGGCACTGGAATCTTCTCATGCACTTGCATGGGCGCTAAAA 1080
MPL_trpB            CGCCATGAAGGCATCATCCCGGCACTGGAATCTTCCCATGCACTTGCGTGGGCGCTCAAA 1080
                    ** *********** ** ************** ** ******** ** ******** ***

MPH_ASIOC_trpB      ATGATCAAACAAAACCCGGATAAAGAACAATTACTGGTTGTTAACTTGTCAGGCCGCGGG 1140
MP23_trpB           ATGGTCAAGCAAAACCCGGATAAAGAACAATTACTGGTTGTTAACTTGTCAGGCCGCGGG 1140
MFB070_trpB         ATGATCAAACAAAACCCGGATAAAGAACAGTTACTGGTTGTTAACTTGTCAGGCCGCGGG 1140
MYI_trpB            ATGATTAAACAAAACCCGGATAAAGAACAGTTACTGGTTGTTAACTTGTCAGGCCGCGGG 1140
MPL_trpB            ATGATCAAACAAAACCCGGATAAAGAACAGTTACTGGTTGTTAACTTGTCAGGCCGCGGG 1140
                    *** * ** ******************** ******************************
                            Reverse primer
MPH_ASIOC_trpB      GATAAAGACATTTTCACGGTTCACGATATTCTGAAATCCCGGGGGGAAATTTGA 1194
MP23_trpB           GATAAAGACATTTTCACGGTTCACGATATTCTGAAATCCCGGGGGGAAATTTGA 1194
MFB070_trpB         GATAAAGACATTTTCACGGTTCACGATATTCTGAAATCCCGGGGGGAAATTTGA 1194
MYI_trpB            GATAAAGACATTTTCACGGTTCACGATATTCTTAAATCCCGGGGAGAAATTTAA 1194
MPL_trpB            GATAAAGACATTTTCACGGTTCACGATATTCTGAAATCCCGGGGGGAAATTTGA 1194
                    ******************************** *********** ******* *
```

FIGURE S5

Clustal Omega v1.2.4 Multiple Sequence Alignment (https://www.ebi.ac.uk/Tools/msa/clustalo/) was used to generate the tryptophan synthase beta chain gene (*trpB*) sequences alignment. The *trpB* sequences were obtained from MGB's genome—selection of *trpB*-gene-targeted primers.

Supplementary Figures

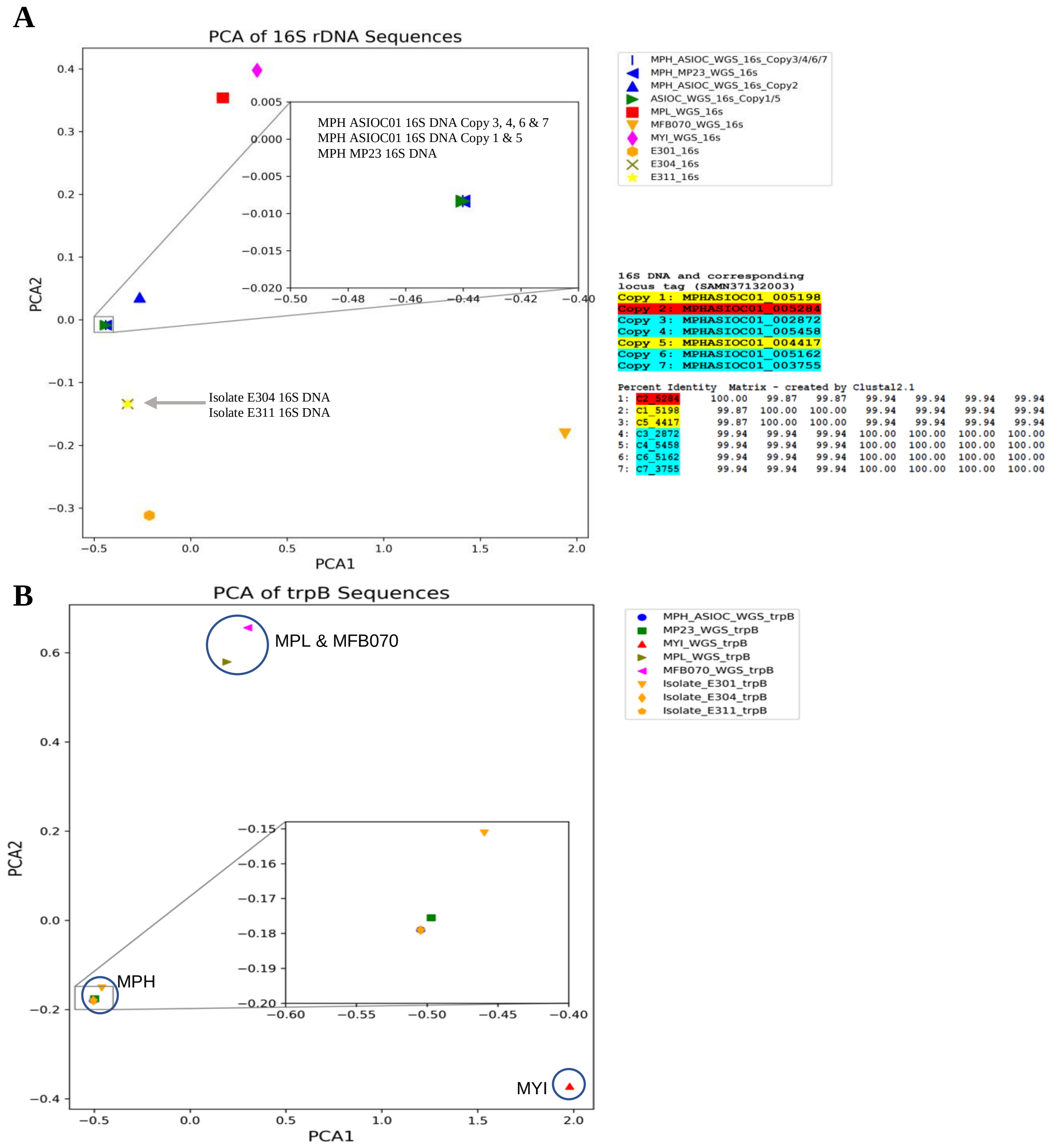

1: C2_5284	100.00	99.87	99.87	99.94	99.94	99.94	99.94
2: C1_5198	99.87	100.00	100.00	99.94	99.94	99.94	99.94
3: C5_4417	99.87	100.00	100.00	99.94	99.94	99.94	99.94
4: C3_2872	99.94	99.94	99.94	100.00	100.00	100.00	100.00
5: C4_5458	99.94	99.94	99.94	100.00	100.00	100.00	100.00
6: C6_5162	99.94	99.94	99.94	100.00	100.00	100.00	100.00
7: C7_3755	99.94	99.94	99.94	100.00	100.00	100.00	100.00

FIGURE S6

PCA analysis of MGB sequences. Sequences were aligned utilizing MAFFT algorithm. Subsequent to alignment, a distance matrix was computed using the 'identity' model in the BioPython framework. This standard distance matrix was transformed into Jaccard distance matrix. PCA was performed on the Jaccard matrix using the scikit-learn library. **(A)** Illustration of the PCA analysis of 10 MGB 16S rDNA sequences. 16S rDNA of MPH ASIOC01 copy 1, 3, 4, 5, 6, 7 and 16S rDNA of MPH MP23^{T} were superimposed at the same position. **(B)** Illustration of the PCA analysis of 8 MGB *trpB* sequences. An inset axis with a zoom factor of 95% was integrated into the plot for enhanced visualization of very closely clustered data points in **(B)**.

Supplementary Figures

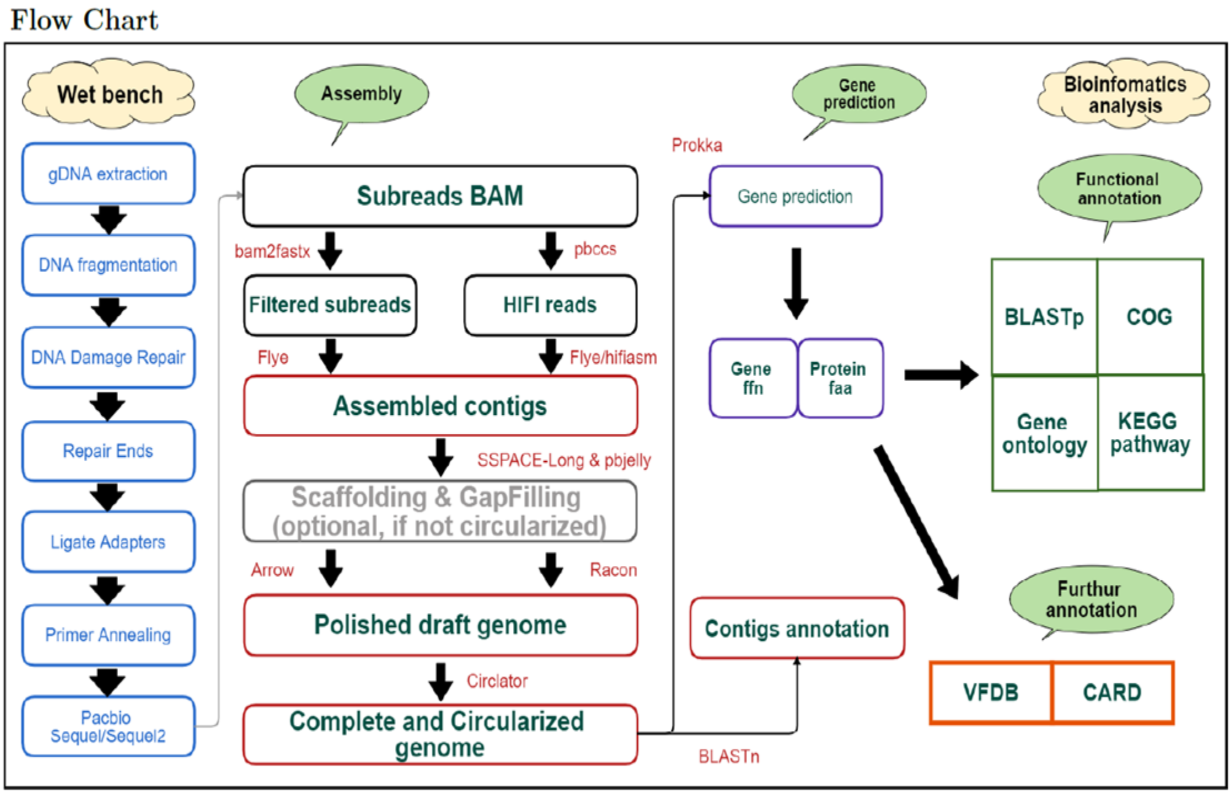

FIGURE S7

Overall PacBio de-novo assembly workflow for MPH ASIOC01.

Supplementary Figures

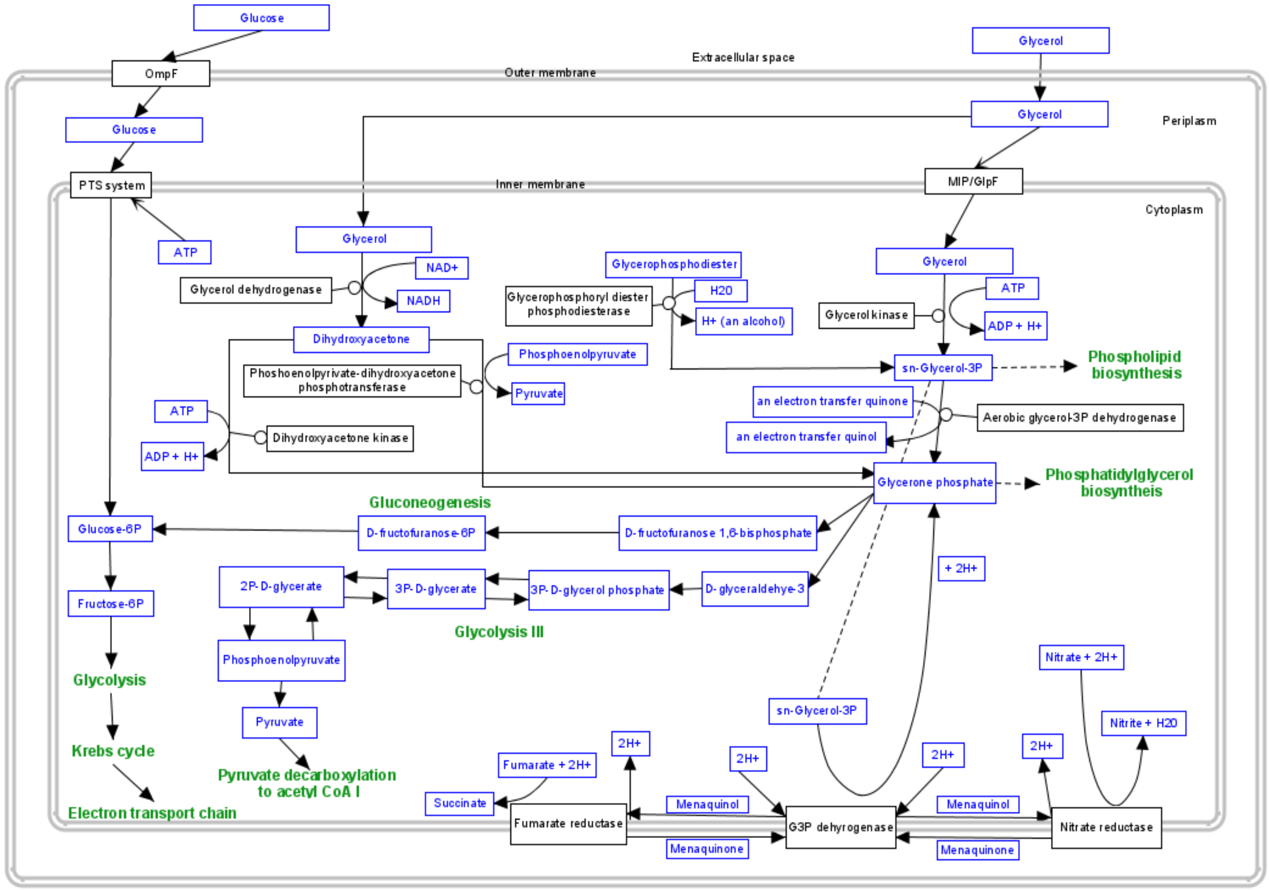

FIGURE S8

Glycerol degradation pathway in MPH ASIOC01. The presence of corresponding enzymes and reaction pathways in MPH ASIOC01 was confirmed with Pathway Tools Bioinformatics Software v27.0. This figure was generated using PathVisio Pathway editor v 3.3.0.

Supplementary Figures

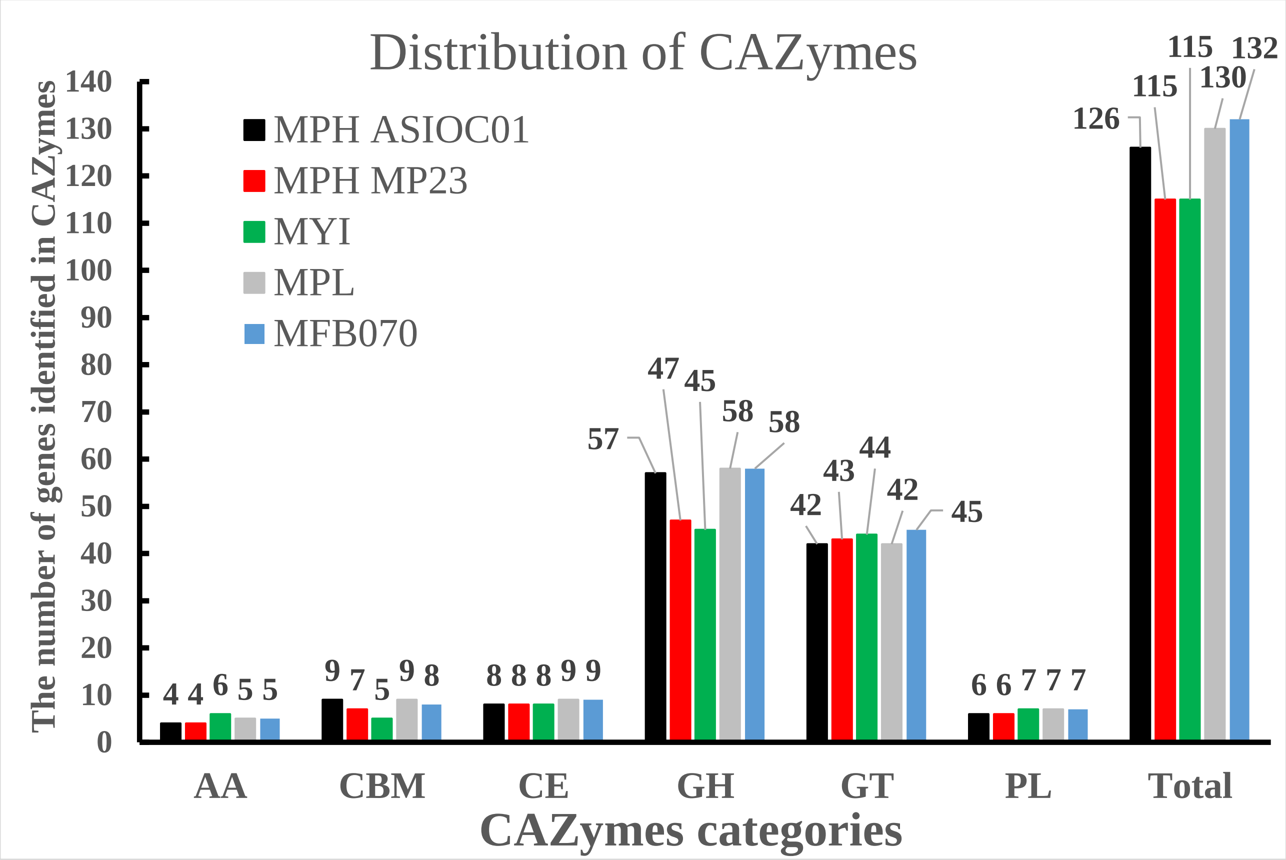

FIGURE S9

CAZymes annotated from the genome of MGBs. The annotation was performed using an automated Carbohydrate-active enzyme server (dbCAN3, https://bcb.unl.edu/dbCAN2/index.php).

Supplementary Figures

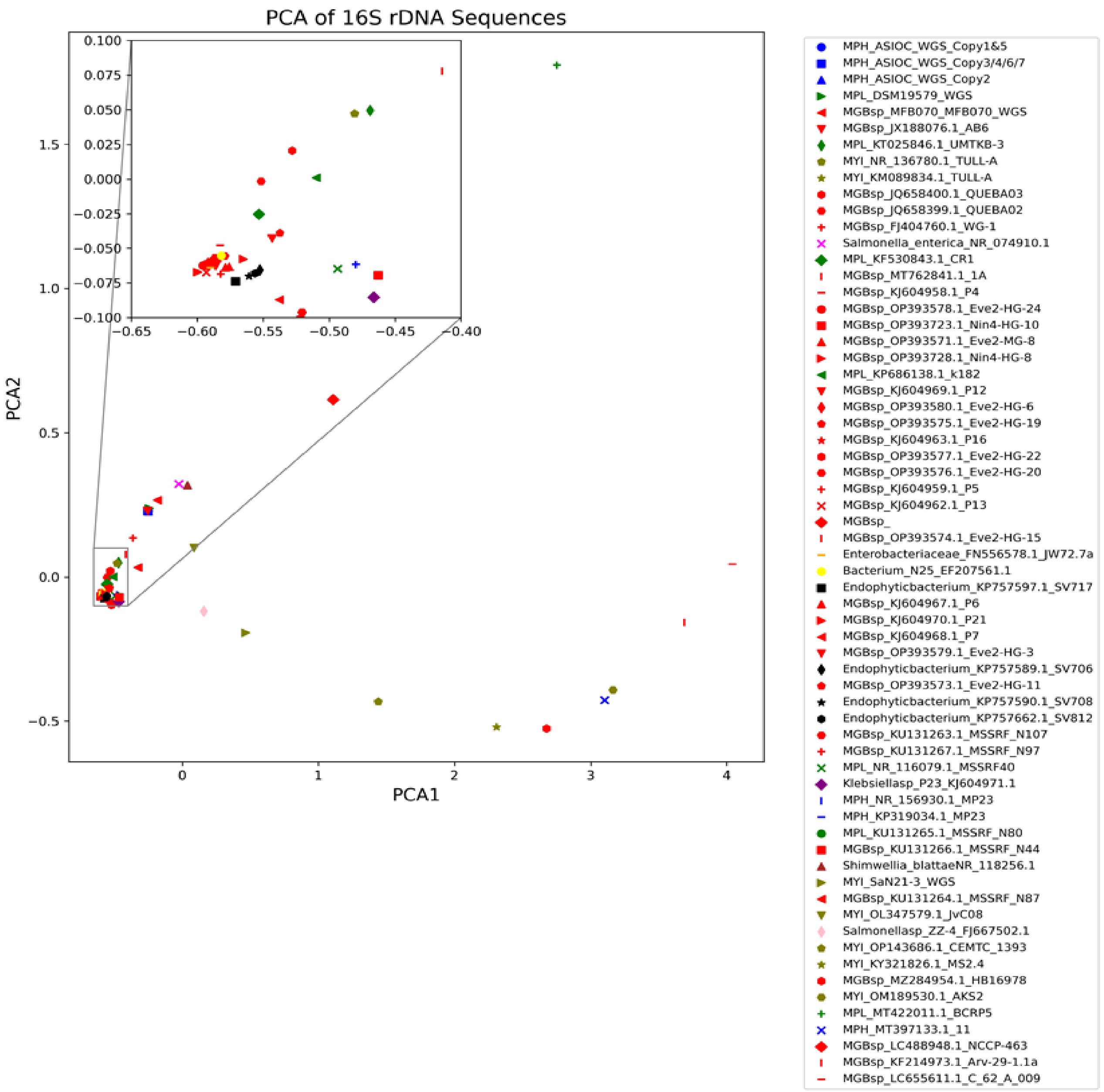

FIGURE S10

PCA analysis of MGB sequences. Sequences were aligned utilizing MAFFT algorithm. Subsequent to alignment, a distance matrix was computed using the 'identity' model in the BioPython framework. This standard distance matrix was transformed into Jaccard distance matrix. PCA was performed on the Jaccard matrix using the scikit-learn library. The figure illustrates the PCA analysis of 64 16S rDNA sequences. An inset axis with a zoom factor of 95% was integrated into the plot for enhanced visualization of very closely clustered data points.

Supplementary Figures

groL - ANI	MPH_ASIOC	MPH_MP23	MYI_SaN21-3	MPL	MFB070	MYI_TULL-A
MPH_ASIOC_groL		99.94	95.14	96.05	96.11	99.34
MPH_MP23_groL			95.08	95.99	96.05	99.02
MYI_SaN21-3_groL				95.51	95.75	93.44
MPL_groL					99.39	93.77
MFB070_groL						93.77
MYI_TULL-A_groL						

Lowest Mid Highest

MGB	Gene	Accession no	bp
MYI TULL-A	Heat shock protein 60 (*hsp60*/*groL*)	KM435308.1	305
MYI TULL-A	Gyrase B subunit (*gyrB*)	KM435307.1	936
MYI TULL-A	Polymerase beta subunit (*rpoB*)	KM435306.1	1090
MYI TULL-A	16S rDNA, partial sequence	NR_136780.1	1464
MYI TULL-A	16S rDNA, partial sequence	KM089834.1	1464
MPH MP23	16S rDNA, partial sequence	NR_156930.1	1368
MPH MP23	16S rDNA, partial sequence	KP319034.1	1368
MPL	16S rDNA, partial sequence	NR_116079.1	1368
MFB070	16S rDNA, partial sequence	KU254647.1	1538

gyrB - ANI	MPH_ASIOC	MPH_MP23	MYI_SaN21-3	MPL	MFB070	MYI_TULL-A
MPH_ASIOC_gyrB		99.46	94.33	95.20	95.00	99.79
MPH_MP23_gyrB			94.46	95.29	95.04	99.36
MYI_SaN21-3_gyrB				95.33	95.82	93.70
MPL_gyrB					97.68	93.91
MFB070_gyrB						93.80
MYI_TULL-A_gyrB						

Lowest Mid Highest

rpoB -ANI	MPH_ASIOC	MPH_MP23	MYI_SaN21-3	MPL	MFB070	MYI_TULL-A
MPH_ASIOC_rpoB		99.8	97.00	97.82	97.92	99.45
MPH_MP23_rpoB			96.97	97.79	97.89	99.36
MYI_SaN21-3_rpoB				98.21	98.16	96.24
MPL_rpoB					99.65	96.97
MFB070_rpoB						97.25
MYI_TULL-A_rpoB						

Lowest Mid Highest

16s RNA -ANI	ASIOC 1	ASIOC 2	ASIOC 3	MP23 WGS	MP23	MP23	WGS SaN21-3	TULL-A	TULL-A	WGS MPL	MPL	WGS MFB070	MFB070
MPH ASIOC WGS V1		99.87	99.93	99.67	100.00	100.00	99.61	99.66	99.66	99.67	99.71	99.15	99.35
MPH ASIOC WGS V2			99.93	99.74	99.93	99.93	99.69	99.80	99.80	99.67	99.63	99.28	99.48
MPH ASIOC WGS V3				99.74	100.00	100.00	99.61	99.73	99.73	99.74	99.71	99.22	99.41
MPH MP23 WGS					99.71	99.71	99.37	99.52	99.52	99.48	99.42	98.97	99.22
MPH MP23 NR_156930.1						100.00	99.58	99.71	99.71	99.71	99.71	99.34	99.63
MPH MP23 KP319034.1							99.58	99.71	99.71	99.71	99.71	99.34	99.63
MYI SaN21-3 WGS								99.60	99.60	99.92	99.92	99.45	99.76
MYI TULL-A NR_136780.1									100.00	99.59	99.56	99.32	99.66
MYI TULL-A KM089834.1										99.59	99.56	99.32	99.66
MPL WGS											100.00	99.48	99.67
MPL MSSRF40 NR_116079.1												99.63	99.93
MFB070 WGS													99.28
MFB070 KU254647.1													

Lowest Mid Highest

Phylogram - *groL*

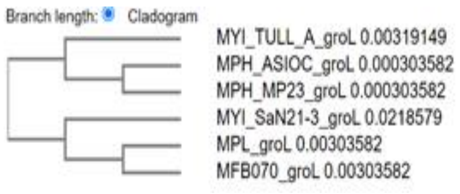

Phylogram - *gyrB*

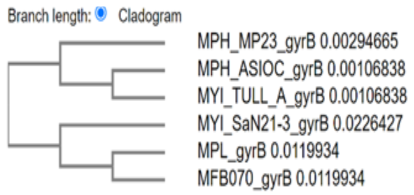

Phylogram - *rpoB*

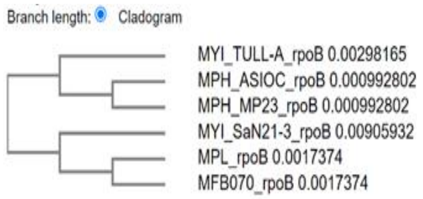

FIGURE S11

Analysis of MYI TULL-A^{T} 16s rDNA and genes. Percent Identity Matrix was created using Clustal Omega v1.2.4. Phylogram were generated using Clustal Omega v1.2.4 Multiple Sequence Alignment (https://www.ebi.ac.uk/Tools/msa/clustalo/). Phylogram, is a tree that depicts the amount of time between each generation or node.

Supplementary Figures

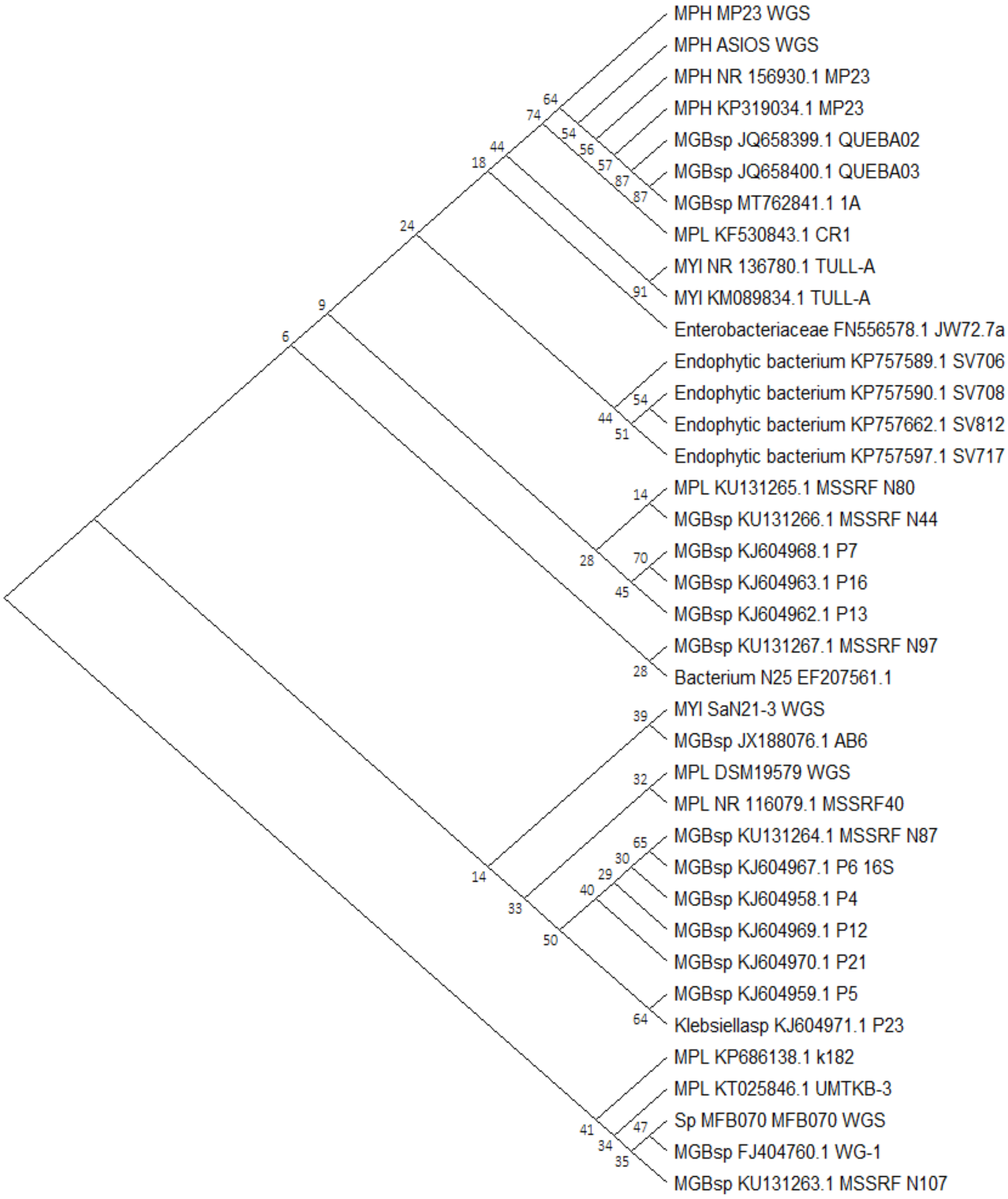

FIGURE S12

Phylogenetic tree of MGBs isolates. 16S rDNA of MGBs were aligned with MEGA v11 using the Multiple Sequence Comparison by Log-Expectation (MUSCLE) alignments program. MGBs with short 16s rDNA readout were removed, and the remaining sequences were trimmed accordingly to ensure that all sequences share an identical nucleotide length. Phylogenetic trees were then constructed and calculated using neighbor-joining algorithm based on the distance between species.

Supplementary Figures

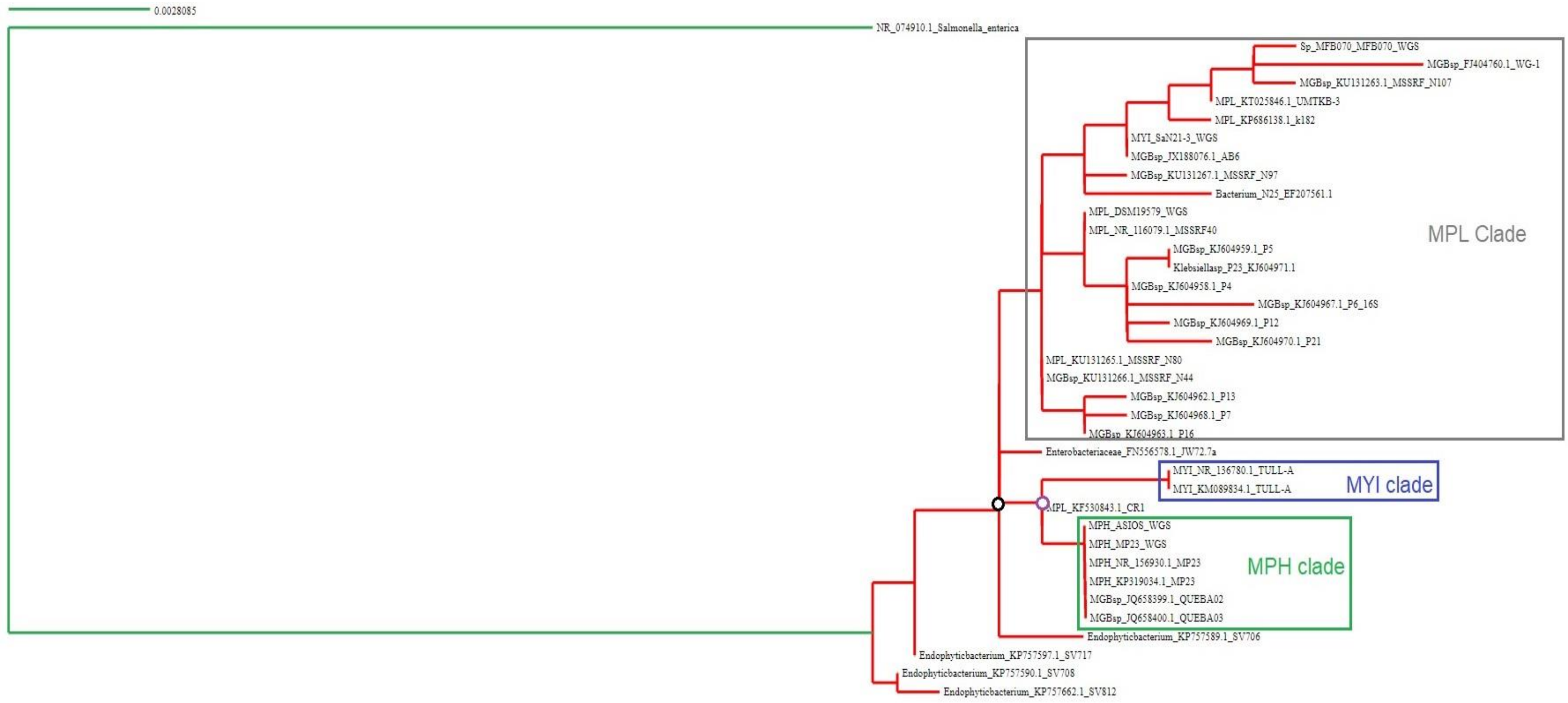

FIGURE S13

Phylogenetic tree of MGBs isolates. 16S rDNA of MGBs were aligned with MEGA v11 using the Multiple Sequence Comparison by Log-Expectation (MUSCLE) alignments program. MGBs with short 16s rRNA readout were removed, and the remaining sequences were trimmed accordingly to ensure that all sequences share an identical nucleotide length. Phylogenetic trees were then constructed and calculated using Maximum likelihood estimation. The phylogenetic tree was then subjected to Multi-rate Poisson tree processes (mPTP) for single-locus species delimitation.

Supplementary Tables

TABLE S1A

Source of genomes and 16s rDNA used in this study.

No	Species	Strain	NCBI Taxonomy ID	Data type	Reference sequence	BioSample ID	Submittion date	Sequencing technology	Source/country
1	MPH	$MP23^T$	1691903	WGS	RefSeq GCF_001655675.1	SAMN05177220	2016/6/3	Illumina MiSeq	India
2	MPL	$MSSRF40^T$	451513	WGS	RefSeq GCF_003182475.1	SAMN09064728	2018/5/31	Illumina HiSeq	India
3	MYI	SaN21-3	1529639	WGS	RefSeq GCF_020523985.1	SAMN21557958	2021/1/13	Illumina	China
4	MBF070	MFB070	1224318	WGS	RefSeq GCF_000705335.1	SAMN02719562	2014/6/12	Illumina MiSeq	India
5	MPH	ASIOC01	1691903	WGS	NA	SAMN37132003	2023/8/24	PacBio Sequel	Taiwan
6	*Salmonella enterica*	LT2	99287	WGS	RefSeq GCF_000006945.2	SAMN02604315	2001/10/26 (revised in 2016/01/13)	Sanger sequencing	USA

MPH: *M. phragmitis*
MPL: *M. plantisponsor*
MYI: *M. yixingensis*
MGB: *Mangrovibcater*
NA: Not applicable

Supplementary Tables

TABLE S1B
List of MGB isolates and type strains.

No	Species	Strain	Accession #	Data type	Country	Source/Host
1	MPH	MP23^T	NR_156930.1	16S rRNA	India	Roots of Phragmites karka
2	MPH	MP23^T	KP319034.1	16S rRNA	India	Roots of Phragmites karka
3	MPH	11	MT397133.1	16S rRNA	China	NA
4	MYI	TULL-A^T	NR_136780.1	16S rRNA	China	Farmland soil
5	MYI	TULL-A^T	KM089834.1	16S rRNA	China	Farmland soil
6	MYI	MS2.4	KY321826.1	16S rRNA	India	Tannery effluent soil
7	MYI	AKS2	OM189530.1	16S rRNA	Bangladesh	Textile Effluent
8	MYI	JvC08	OL347579.1	16S rRNA	India	NA/*Euryphorus nordmannii**
9	MYI	CEMTC 1393	OP143686.1	16S rRNA	Russian	Geothermal lake
10	MPL	CR1	KF530843.1	16S rRNA	China	Tannery effluent sludge
11	MPL	k182	KP686138.1	16S rRNA	China	N/A
12	MPL	UMTKB-3	KT025846.1	16S rRNA	Malaysia	Brackish water
13	MPL	MSSRF40^T	NR_116079.1	16S rRNA	India	Mangrove-associated wild rice
14	MPL	MSSRF N80	KU131265.1	16S rRNA	India	Mangrove rhizosphere
15	MPL	BCRP5	MT422011.1	16S rRNA	India	Mangrove root endophyte
16	MGB sp.	MSSRF N44	KU131266.1	16S rRNA	India	Mangrove rhizosphere
17	MGB sp.	MSSRF N87	KU131264.1	16S rRNA	India	Mangrove rhizosphere
18	MGB sp.	MSSRF N97	KU131267.1	16S rRNA	India	Mangrove rhizosphere
19	MGB sp.	MSSRF N107	KU131263.1	16S rRNA	India	Mangrove rhizosphere
20	MGB sp.	Arv-29-1.1a	KF214973.1	16S rRNA	USA	Pine tree/*Bursaphelenchus xylophilus* (pine wood nematode)
21	MGB sp.	P4	KJ604958.1	16S rRNA	India	Mangrove rhizosphere
22	MGB sp.	P5	KJ604959.1	16S rRNA	India	Mangrove rhizosphere
23	MGB sp.	P6	KJ604967.1	16S rRNA	India	Mangrove rhizosphere
24	MGB sp.	P7	KJ604968.1	16S rRNA	India	Mangrove rhizosphere
25	MGB sp.	P12	KJ604969.1	16S rRNA	India	Mangrove rhizosphere
26	MGB sp.	P13	KJ604962.1	16S rRNA	India	Mangrove rhizosphere
27	MGB sp.	P16	KJ604963.1	16S rRNA	India	Mangrove rhizosphere
28	MGB sp.	P21	KJ604970.1	16S rRNA	India	Mangrove rhizosphere
29	MGB sp.	AB6	JX188076.1	16S rRNA	India	Soil sediment from brackish water
30	MGB sp.	QUEBA02	JQ658399.1	16S rRNA	Brazil	Todos os Santos Bay oil-contaminated mangrove
31	MGB sp.	QUEBA03	JQ658400.1	16S rRNA	Brazil	Todos os Santos Bay oil-contaminated mangrove
32	MGB sp.	WG-1	FJ404760.1	16S rRNA	China	Sewage
33	MGB sp.	1A	MT762841.1	16S rRNA	Taiwan	Copper containing wastewater
34	MGB sp.	Nin4-HG-8 OP393728.1	OP393728.1	16S rRNA	Hong Kong	Hindgut of *Neosarmatium indicum*
35	MGB sp.	Nin4-HG-10 OP393723.1	OP393723.1	16S rRNA	Hong Kong	Hindgut of *Neosarmatium indicum*
36	MGB sp.	Eve2-MG-8 OP393571.1	OP393571.1	16S rRNA	Hong Kong	Midgut of *Episesarma versicolor*
37	MGB sp.	Eve2-HG-1 OP393572.1	OP393572.1	16S rRNA	Hong Kong	Hindgut of *Episesarma versicolor*
38	MGB sp.	Eve2-HG-11 OP393573.1	OP393573.1	16S rRNA	Hong Kong	Hindgut of *Episesarma versicolor*
39	MGB sp.	Eve2-HG-15 OP393574.1	OP393574.1	16S rRNA	Hong Kong	Hindgut of *Episesarma versicolor*
40	MGB sp.	Eve2-HG-19 OP393575.1	OP393575.1	16S rRNA	Hong Kong	Hindgut of *Episesarma versicolor*
41	MGB sp.	Eve2-HG-20 OP393576.1	OP393576.1	16S rRNA	Hong Kong	Hindgut of *Episesarma versicolor*
42	MGB sp.	Eve2-HG-22 OP393577.1	OP393577.1	16S rRNA	Hong Kong	Hindgut of *Episesarma versicolor*
43	MGB sp.	Eve2-HG-24 OP393578.1	OP393578.1	16S rRNA	Hong Kong	Hindgut of *Episesarma versicolor*
44	MGB sp.	Eve2-HG-3 OP393579.1	OP393579.1	16S rRNA	Hong Kong	Hindgut of *Episesarma versicolor*
45	MGB sp.	Eve2-HG-6 OP393580.1	OP393580.1	16S rRNA	Hong Kong	Hindgut of *Episesarma versicolor*
46	MGB sp.	Endophytic bacterium SV706	KP757589.1	16S rRNA	USA	Host:Spartina alterniflora (salt-water cordgrass)
47	MGB sp.	Endophytic bacterium SV708	KP757590.1	16S rRNA	USA	Host:Spartina alterniflora (salt-water cordgrass)
48	MGB sp.	Endophytic bacterium SV717	KP757597.1	16S rRNA	USA	Host:Spartina alterniflora (salt-water cordgrass)
49	MGB sp.	Endophytic bacterium SV812	KP757662.1	16S rRNA	USA	Host:Spartina alterniflora (salt-water cordgrass)
50	MGB sp.	Enterobacteriaceae bacterium JW72.7a	FN556578.1	16S rRNA	UK	Automotive biodiesel
51	MGB sp.	Bacterium N25	EF207561.1	16S rRNA	China	Mangrove system sediment
52	MGB sp.	Klebsiella sp. P23 (99.75% ANI with MYI)	KJ604971.1	16S rRNA	India	Mangrove rhizosphere
53	MGB sp.	Salmonella sp. ZZ-4 (98.99% ANI with MYI)	FJ667502.1	16S rRNA	China	Activated sludge from an oil field wastewater-treating system

MGB sp.: Re-assigned by this work according to their high 16sRNA ANI similarity (>98%) highlighted in blue.
N/A Not applicable

Supplementary Tables

TABLE S2

Subsystem analysis for MGBs. These outcomes were generated using PATRIC v3.6.12 (Comprehensive Genome Analysis).

Features	*M. phragmitis* (subsystems #, Genes #)	*M. phragmitis* (subsystems #, Genes #)	*M. plantisponsor* (subsystems #, Genes #)	*M. yixingensis* (subsystems #, Genes #)	*Mangrovibacter* sp. (subsystems #, Genes #)
GenBank assembly accession	This study	GCA_001655675.1	GCA_003182475.1	GCA_020523985.1	GCA_000705335.1
Strain	ASIOC01	MP23^{T}	MSSRF40^{T}	SaN21-3	MFB070
Metabolism	110, 962	106, 890	115, 1021	113, 996	114, 1030
Protein processing	46, 251	46, 254	46, 253	46, 255	46, 255
Stress response, defense & virulence	38, 186	38, 180	39, 180	39, 180	39, 180
Energy	36, 359	35, 343	35, 335	34, 318	35, 338
Membrane transport	25, 207	23, 189	26, 178	26, 175	25, 158
Cellular processes	21, 178	20, 94	20, 169	19, 90	20, 139
DNA processing	20, 131	19, 161	19, 94	19, 164	20, 102
RNA processing	14, 73	14, 74	14, 73	14, 74	14, 72
Cell envelope	11, 92	11, 84	12, 93	11, 86	11, 95
Miscellaneous	10, 92	8, 36	8, 42	8, 40	8, 40
Regulation and cell signaling	7, 26	7, 26	7, 26	7, 26	7, 26
Prediction tool	PATRIC v3.6.12	PATRIC v3.6.12	PATRIC v3.6.12	PATRIC v3.6.12	PATRIC v3.6.12

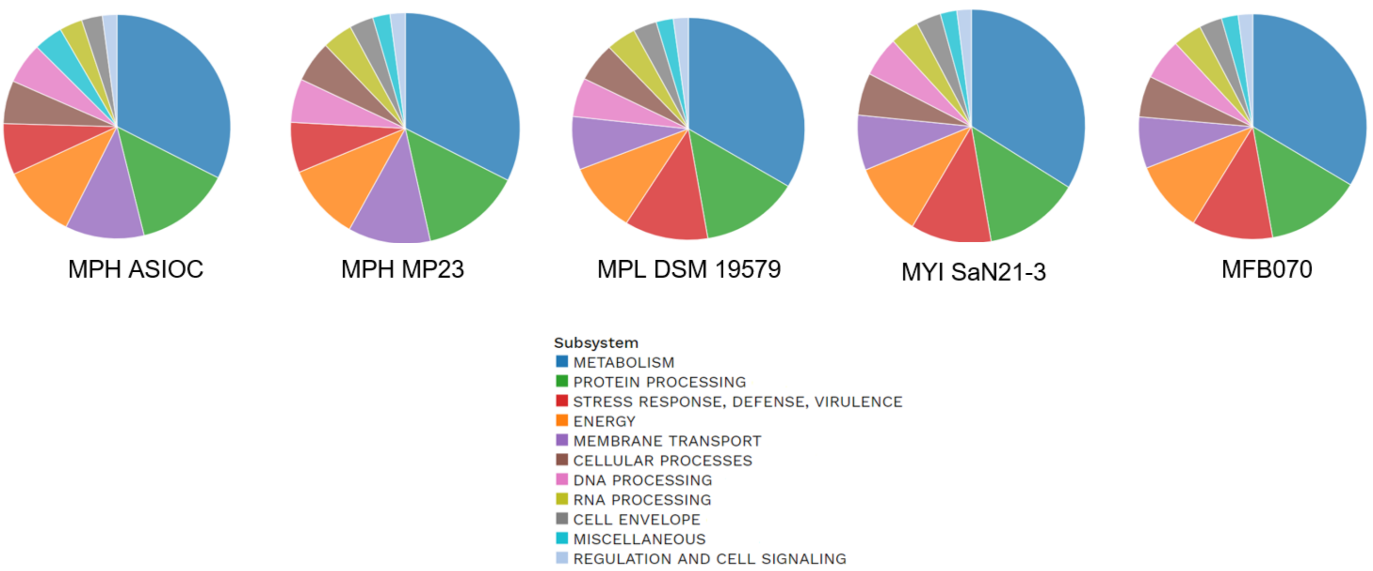

Supplementary Tables

TABLE S3

List of CAZyme annotations using dbCAN3 server (https://bcb.unl.edu/dbCAN2/index.php).

No.	Locus tag (SAMN37132003)	Gene	Gene product	EC #	Length	EC# (dbCAN3 server)	dbCAN_sub (HMMER)
1	MPHASIOC01_004483	*cueO*	multicopper oxidase CueO	1.16.3.1	537	NA	AA1_e20
2	MPHASIOC01_000973	*katG*	catalase/peroxidase HPI	1.11.1.21	721	NA	AA2_e1
3	MPHASIOC01_001255	*betA*	choline dehydrogenase	1.1.99.1	569	NA	AA3_e57
4	MPHASIOC01_000113	NA	flavodoxin family protein	NA	183	NA	AA6_e2
5	MPHASIOC01_004781	NA	discoidin domain-containing protein	NA	159	NA	CBM32_e12
6	MPHASIOC01_004189	*malZ*	maltodextrin glucosidase	3.2.1.20	605	3.2.1.20|3.2.1.1|3.2.1.133	CBM34_e8+GH13_e88
7	MPHASIOC01_003873	*glgB*	1,4-alpha-glucan branching enzyme	2.4.1.18	733	2.4.1.18	CBM48_e2+CBM48_e2+GH13_e200
8	MPHASIOC01_003872	*glgX*	glycogen debranching protein GlgX	3.2.1.196	658	3.2.1.68|2.4.1.25|3.2.1.-	CBM48_e3+GH13_e48
9	MPHASIOC01_003296	NA	peptidoglycan DD-metalloendopeptidase family protein	NA	234	NA	CBM50_e36
10	MPHASIOC01_003173	*nlpD*	murein hydrolase activator NlpD	NA	357	NA	CBM50_e486
11	MPHASIOC01_002955	NA	hypothetical protein	NA	96	NA	CBM50_e505
12	MPHASIOC01_001541	*mepM*	murein DD-endopeptidase MepM	3.4.24.-	447	NA	CBM50_e567
13	MPHASIOC01_004268	*lpxC*	UDP-3-O-acyl-N-acetylglucosamine deacetylase	3.5.1.108	318	3.5.1.108	CE11_e22
14	MPHASIOC01_004510	*lpxC*	UDP-3-O-acyl-N-acetylglucosamine deacetylase	3.5.1.108	305	3.5.1.108	CE11_e22
15	MPHASIOC01_000109	NA	GDSL-type esterase/lipase family protein	NA	529	3.1.1.-	CE12_e2
16	MPHASIOC01_003500	NA	polysaccharide deacetylase	NA	282	NA	CE4_e121
17	MPHASIOC01_004400	*puuE*	allantoinase PuuE	NA	318	3.5.1.41	CE4_e177
18	MPHASIOC01_000108	NA	pectinesterase family protein	NA	361	3.1.1.11	CE8_e1
19	MPHASIOC01_000480	NA	putative acyl-CoA thioester hydrolase	NA	434	NA	CE8_e3
20	MPHASIOC01_003912	*nagA*	N-acetylglucosamine-6-phosphate deacetylase	3.5.1.25	382	3.5.1.25	CE9_e0
21	MPHASIOC01_003309	NA	6-phospho-beta-glucosidase	3.2.1.86	477	3.2.1.86|3.2.1.23|3.2.1.21	GH1_e0
22	MPHASIOC01_005077	NA	glycoside hydrolase family 1 protein	3.2.1.-	462	3.2.1.86|3.2.1.85|3.2.1.21	GH1_e13
23	MPHASIOC01_003247	NA	glycoside hydrolase family 1 protein	3.2.1.-	475	NA	GH1_e42
24	MPHASIOC01_003220	*mltA*	murein transglycosylase A	4.2.2.-	364	4.2.2.n1	GH102_e2
25	MPHASIOC01_003108	*mltB*	lytic murein transglycosylase B	4.2.2.-	361	4.2.2.n1	GH103_e0
26	MPHASIOC01_001560	NA	glycoside hydrolase family 88 protein	NA	379	NA	GH105_e57
27	MPHASIOC01_003590	NA	glycosyl hydrolase 108 family protein	NA	193	NA	GH108_e60
28	MPHASIOC01_000233	NA	glycoside hydrolase family 127 protein	NA	658	3.2.1.185	GH127_e0
29	MPHASIOC01_004872	*treC*	alpha,alpha-phosphotrehalase	3.2.1.93	554	3.2.1.93	GH13_e1
30	MPHASIOC01_000238	NA	alpha-amylase	3.2.1.1	676	3.2.1.-|3.2.1.1	GH13_e62
31	MPHASIOC01_003068	NA	DUF2264 domain-containing protein	NA	580	NA	GH154_e18
32	MPHASIOC01_004143	NA	beta-galactosidase	NA	1037	3.2.1.23	GH2_e92
33	MPHASIOC01_003398	NA	transglycosylase SLT domain-containing protein	NA	196	NA	GH23_e145
34	MPHASIOC01_003354	*mltC*	membrane-bound lytic murein transglycosylase MltC	4.2.2.-	360	4.2.2.n1	GH23_e150
35	MPHASIOC01_004611	NA	lytic transglycosylase domain-containing protein	NA	182	NA	GH23_e2
36	MPHASIOC01_002841	*mltF*	membrane-bound lytic murein transglycosylase MltF	4.2.2.-	481	4.2.2.n1	GH23_e225
37	MPHASIOC01_002052	NA	lytic transglycosylase domain-containing protein	NA	192	NA	GH23_e361
38	MPHASIOC01_001747	NA	lytic transglycosylase domain-containing protein	NA	175	NA	GH23_e410
39	MPHASIOC01_000717	NA	lytic transglycosylase domain-containing protein	4.2.2.n1	639	NA	GH23_e704
40	MPHASIOC01_001465	*emtA*	membrane-bound lytic murein transglycosylase EmtA	4.2.2.-	202	4.2.2.n1	GH23_e734

41	MPHASIOC01_004410	*mltD*	murein transglycosylase D	4.2.2.-	454	NA	GH23_e819+CBM50_e338
42	MPHASIOC01_003404	NA	lytic transglycosylase domain-containing protein	NA	159	NA	GH23_e85
43	MPHASIOC01_004591	*sltY*	murein transglycosylase	4.2.2.-	644	4.2.2.n1	GH23_e96
44	MPHASIOC01_000695	NA	lysozyme	NA	149	NA	GH24_e250
45	MPHASIOC01_003867	NA	glycoside hydrolase family protein	NA	258	NA	GH24_e271
46	MPHASIOC01_000471	NA	glycoside hydrolase family protein	NA	148	NA	GH24_e305
47	MPHASIOC01_002537	NA	lysozyme	NA	165	3.2.1.17	GH24_e32
48	MPHASIOC01_000937	NA	lysozyme	NA	178	NA	GH24_e98
49	MPHASIOC01_002133	NA	glycosyl hydrolase family 28 protein	NA	441	NA	GH28_e5
50	MPHASIOC01_003448	NA	glycosyl hydrolase family 28 protein	NA	450	NA	GH28_e74
51	MPHASIOC01_002426	*bglX*	beta-glucosidase BglX	3.2.1.21	765	3.2.1.21|3.2.1.-	GH3_e1
52	MPHASIOC01_000854	*nagZ*	beta-N-acetylhexosaminidase	3.2.1.52	341	3.2.1.52	GH3_e19
53	MPHASIOC01_001062	NA	glycoside hydrolase family 3 N-terminal domain-containing protein	NA	792	3.2.1.37|3.2.1.55|3.2.1.21	GH3_e88
54	MPHASIOC01_000181	*yicI*	alpha-xylosidase	3.2.1.177	773	3.2.1.177	GH31_e31
55	MPHASIOC01_000521	NA	glycoside hydrolase family 31 protein	NA	788	NA	GH31_e60
56	MPHASIOC01_002161	NA	alpha-glucosidase	3.2.1.20	678	3.2.1.199	GH31_e85
57	MPHASIOC01_000306	NA	sucrose-6-phosphate hydrolase	3.2.1.26	472	3.2.1.26	GH32_e57
58	MPHASIOC01_001463	*treA*	alpha,alpha-trehalase TreA	NA	590	3.2.1.28	GH37_e6
59	MPHASIOC01_004763	NA	alpha-glucosidase/alpha-galactosidase	NA	451	3.2.1.22	GH4_e0
60	MPHASIOC01_002798	NA	6-phospho-beta-glucosidase	3.2.1.86	442	3.2.1.86	GH4_e24
61	MPHASIOC01_004025	NA	6-phospho-alpha-glucosidase	NA	441	3.2.1.122	GH4_e4
62	MPHASIOC01_004777	NA	6-phospho-alpha-glucosidase	NA	441	3.2.1.122	GH4_e4
63	MPHASIOC01_005106	NA	beta-galactosidase	3.2.1.23	673	3.2.1.-	GH42_e0
64	MPHASIOC01_004135	NA	beta-galactosidase	NA	685	3.2.1.23	GH42_e20
65	MPHASIOC01_004496	NA	family 43 glycosylhydrolase	NA	315	3.2.1.-	GH43_e105
66	MPHASIOC01_000013	NA	cellulase family glycosylhydrolase	NA	299	3.2.1.4|3.2.1.8|3.2.1.73	GH5_e77
67	MPHASIOC01_004136	NA	arabinogalactan endo-beta-1,4-galactanase	NA	400	3.2.1.89	GH53_e15
68	MPHASIOC01_000828	*flgJ*	flagellar assembly peptidoglycan hydrolase FlgJ	NA	322	3.2.1.-	GH73_e114
69	MPHASIOC01_000239	NA	protein bax	NA	273	NA	GH73_e253
70	MPHASIOC01_003860	*malQ*	4-alpha-glucanotransferase	2.4.1.25	698	2.4.1.25	GH77_e27
71	MPHASIOC01_000290	*bcsZ*	cellulose synthase complex periplasmic endoglucanase BcsZ	3.2.1.4	369	3.2.1.4|3.2.1.6	GH8_e0
72	MPHASIOC01_000135	NA	glycosyl hydrolase family 8	NA	336	3.2.1.4|3.2.1.6|3.2.1.73	GH8_e29
73	MPHASIOC01_000282	NA	glycosyl hydrolase family 8	NA	333	3.2.1.4|3.2.1.6|3.2.1.73	GH8_e29
74	MPHASIOC01_003064	NA	glycoside hydrolase family 88 protein	NA	393	NA	GH88_e28
75	MPHASIOC01_004436	*lpxB*	lipid-A-disaccharide synthase	2.4.1.182	382	2.4.1.182	GT19_e17
76	MPHASIOC01_000343	*arnC*	undecaprenyl-phosphate 4-deoxy-4-formamido-L-arabinose transferase	2.4.2.53	324	NA	GT2
77	MPHASIOC01_000802	*mdoH*	glucans biosynthesis glucosyltransferase MdoH	2.4.1.-	838	NA	GT2
78	MPHASIOC01_002275	NA	glycosyltransferase family 2 protein	2.4.-.-	339	NA	GT2
79	MPHASIOC01_002280	NA	glycosyltransferase family 2 protein	2.4.-.-	95	NA	GT2
80	MPHASIOC01_002308	*wcaA*	colanic acid biosynthesis glycosyltransferase WcaA	NA	281	NA	GT2
81	MPHASIOC01_004313	NA	glycosyltransferase	2.4.-.-	563	NA	GT2
82	MPHASIOC01_004671	NA	glycosyltransferase family 2 protein	NA	246	NA	GT2
83	MPHASIOC01_005384	NA	glycosyltransferase family 2 protein	2.4.-.-	303	NA	GT2
84	MPHASIOC01_005390	NA	glycosyltransferase	2.4.-.-	333	NA	GT2
85	MPHASIOC01_005392	NA	glycosyltransferase family 2 protein	2.4.-.-	257	2.4.1.-	GT2
86	MPHASIOC01_002304	*wcaE*	colanic acid biosynthesis glycosyltransferase WcaE	2.4.1.-	250	NA	GT2
87	MPHASIOC01_000141	*bcsA*	UDP-forming cellulose synthase catalytic subunit	NA	705	2.4.1.12|2.4.1.-	GT2+GT2
88	MPHASIOC01_000278	*bcsA*	UDP-forming cellulose synthase catalytic subunit	NA	708	2.4.1.12|2.4.1.-	GT2+GT2
89	MPHASIOC01_000288	*bcsA*	UDP-forming cellulose synthase catalytic subunit	2.4.1.12	876	2.4.1.12|2.4.1.-	GT2+GT2

90	MPHASIOC01_001577	*otsA*	alpha,alpha-trehalose-phosphate synthase	2.4.1.15	473	2.4.1.15	GT20_e1
91	MPHASIOC01_005249	*wecG*	lipopolysaccharide N-acetylmannosaminouronosyltransferase	2.4.1.180	246	2.4.1.-	GT26_e141
92	MPHASIOC01_004515	*murG*	undecaprenyldiphospho-muramoylpentapeptide beta-N-acetylglucosaminyltransferase	2.4.1.227	355	2.4.1.227	GT28_e46
93	MPHASIOC01_005391	*waaA*	lipid IV(A) 3-deoxy-D-manno-octulosonic acid transferase	2.4.99.12	424	2.4.99.-	GT30_e0
94	MPHASIOC01_003861	*malP*	maltodextrin phosphorylase	2.4.1.1	800	2.4.1.1	GT35_e0
95	MPHASIOC01_003869	*glgP*	glycogen phosphorylase	2.4.1.1	815	2.4.1.1	GT35_e0
96	MPHASIOC01_002306	NA	glycosyltransferase	2.4.-.-	404	NA	GT4_e1042
97	MPHASIOC01_002299	*wcaI*	colanic acid biosynthesis fucosyltransferase WcaI	NA	408	NA	GT4_e2254
98	MPHASIOC01_005389	NA	glycosyltransferase	2.4.-.-	365	NA	GT4_e2866
99	MPHASIOC01_005388	NA	glycosyltransferase family 4 protein	2.4.-.-	375	NA	GT4_e3707
100	MPHASIOC01_002293	*wcaL*	colanic acid biosynthesis glycosyltransferase WcaL	2.4.1.-	406	NA	GT4_e731
101	MPHASIOC01_002274	NA	glycosyltransferase family 4 protein	NA	384	NA	GT4_e774
102	MPHASIOC01_001626	NA	methyltransferase regulatory domain-containing protein	NA	1134	NA	GT41_e14
103	MPHASIOC01_003870	*glgA*	glycogen synthase GlgA	2.4.1.21	477	2.4.1.21	GT5_e31
104	MPHASIOC01_003709	*mtgA*	monofunctional biosynthetic peptidoglycan transglycosylase	2.4.1.129	242	2.4.1.129	GT51_e0
105	MPHASIOC01_003842	*mrcA*	peptidoglycan glycosyltransferase/peptidoglycan DD-transpeptidase MrcA	2.4.1.129	849	2.4.1.129	GT51_e130
106	MPHASIOC01_004466	*mrcB*	bifunctional glycosyl transferase/transpeptidase	2.4.1.129	849	2.4.1.129	GT51_e28
107	MPHASIOC01_002816	*pbpC*	peptidoglycan glycosyltransferase PbpC	2.4.1.129	778	NA	GT51_e71
108	MPHASIOC01_005251	NA	TDP-N-acetylfucosamine:lipid II N-acetylfucosaminyltransferase	2.4.1.325	358	2.4.1.-	GT56_e0
109	MPHASIOC01_001651	NA	glycosyl transferase	NA	406	NA	GT81_e1
110	MPHASIOC01_000346	*arnT*	lipid IV(A) 4-amino-4-deoxy-L-arabinosyltransferase	2.4.2.43	549	2.4.2.43	GT83_e0
111	MPHASIOC01_004669	NA	glycosyltransferase family 39 protein	2.4.-.-	540	NA	GT83_e31
112	MPHASIOC01_005387	*rfaQ*	putative lipopolysaccharide heptosyltransferase III	NA	357	2.4.99.-	GT9_e18
113	MPHASIOC01_005377	NA	glycosyltransferase family 9 protein	NA	365	NA	GT9_e21
114	MPHASIOC01_005383	NA	glycosyltransferase family 9 protein	2.4.-.-	366	NA	GT9_e292
115	MPHASIOC01_005382	*rfaC*	lipopolysaccharide heptosyltransferase RfaC	2.4.-.-	327	2.4.99.-	GT9_e71
116	MPHASIOC01_005381	*rfaF*	ADP-heptose--LPS heptosyltransferase RfaF	NA	348	2.4.99.-	GT9_e84
117	MPHASIOC01_003478	NA	heparinase II/III family protein	NA	670	NA	PL17_e0
118	MPHASIOC01_003186	NA	heparinase II/III family protein	NA	701	4.2.2.26	PL17_e6+PL17_e6
119	MPHASIOC01_000107	NA	pectate lyase	NA	567	4.2.2.2	PL2_e0
120	MPHASIOC01_003259	NA	oligogalacturonate lyase family protein	NA	376	4.2.2.6	PL22_e0+PL22_e0
121	MPHASIOC01_003261	NA	oligogalacturonate lyase family protein	NA	391	4.2.2.6	PL22_e0+PL22_e0
122	MPHASIOC01_005483	NA	right-handed parallel beta-helix repeat-containing protein	NA	712	4.2.2.9	PL9_e1

NA: No information available

Supplementary Tables

TABLE S4

CAZyme families/subfamilies associated with the cellulose metabolic pathway and their respective EC numbers.

Pathways	Locus tag (SAMN37132003)	Gene	Gene product	Length of AA	EC# (PGAP)	EC# (dbCAN3)	HMMER	dbCAN_sub	DIAMOND	Generalized	Remark
Cellulose degradation	MPHASIOC01_000013	NIA	Cellulase family glycosylhydrolase	299	NIA	3.2.1.4|3.2.1.8|3.2.1.73	GH5_25(41-252)	GH5_e77	GH5	GH5	Cellulase (glycosyl hydrolase family 5), cellulase family A
Cellulose degradation	MPHASIOC01_000135	NIA	Glycosyl hydrolase family 8 (Endoglucanase)	336	NIA	3.2.1.4|3.2.1.6|3.2.1.73	GH8(28-331)	GH8_e29	GH8	GH8	Glycosyl hydrolases family 8, cellulase family D
Cellulose degradation	MPHASIOC01_000290	*bcsZ*	Cellulose synthase complex periplasmic endoglucanase	369	3.2.1.4	3.2.1.4|3.2.1.6	GH8(27-344)	GH8_e0	GH8	GH8	Endo-1,4-D-glucanase
Cellulose degradation	MPHASIOC01_002426	*bglX*	β-glucosidase	765	3.2.1.21	3.2.1.21|3.2.1.-	GH3(98-319)	GH3_e1	GH3	GH3	O-glucosyl hydrolase activity
Cellulose biosynthesis	MPHASIOC01_000141	*bcsA*	UDP-forming cellulose synthase catalytic subunit	705	NIA	2.4.1.12|2.4.1.-	GT2(130-301)	GT2+GT2	GT2	GT2	GO:0016759: cellulose synthase activity
Cellulose biosynthesis	MPHASIOC01_000278	*bcsA*	UDP-forming cellulose synthase catalytic subunit	708	NIA	2.4.1.12|2.4.1.-	GT2(131-302)	GT2+GT2	GT2	GT2	GO:0016759: cellulose synthase activity
Cellulose biosynthesis	MPHASIOC01_000288	*bcsA*	UDP-forming cellulose synthase catalytic subunit	876	2.4.1.12	2.4.1.12|2.4.1.-	GT2(276-446)	GT2+GT2	GT2	GT2	GO:0016760: cellulose synthase (UDP-forming) activity
Cellulose biosynthesis	MPHASIOC01_000140	*bcsB*	Cellulose biosynthesis cyclic di-GMP-binding regulatory protein	778	NIA	NIA	NIA	NIA	GT2	GT2	GO:0030244: cellulose biosynthetic process
Cellulose biosynthesis	MPHASIOC01_000279	*bcsB*	Cellulose biosynthesis cyclic di-GMP-binding regulatory protein	793	NIA	NIA	NIA	NIA	GT2	GT2	GO:0030244: cellulose biosynthetic process
Cellulose biosynthesis	MPHASIOC01_000289	*bcsB*	Cellulose biosynthesis cyclic di-GMP-binding regulatory protein	782	NIA	NIA	NIA	NIA	GT2	GT2	GO:0030244: cellulose biosynthetic process

NIA: No information available

Supplementary Tables

TABLE S5

An overview of the MGB's secondary metabolite biosynthetic gene clusters (BGCs) distribution predicted using antiSMASH version 7.0.0 (https://antismash.secondarymetabolites.org/#!/start).

Strain	Region	Type	From	To	Most similar known cluster & type	Similarity	Size (bp)	Biological activities
MPH ASIOC01	Region 1	Thiopeptide	624,326	650,698	O-antigen (Saccharide)	14%	26,372	Major component of the surface lipopolysaccharide (LPS) of Gram-negative bacteria
MPH MP23	Region 43.1	Thiopeptide	111,096	137,468	O-antigen (Saccharide)	14%	26,372	Major component of the surface lipopolysaccharide (LPS) of Gram-negative bacteria
MYI	Region 1.2	Thiopeptide	797,886	824,260	O-antigen (Saccharide)	14%	26,374	Major component of the surface lipopolysaccharide (LPS) of Gram-negative bacteria
MPL	Region 4.1	Thiopeptide	289,826	316,200	O-antigen (Saccharide)	14%	26,374	Major component of the surface lipopolysaccharide (LPS) of Gram-negative bacteria
MFB070	Region 20.1	Thiopeptide	80,832	107,239	O-antigen (Saccharide)	14%	26,407	Major component of the surface lipopolysaccharide (LPS) of Gram-negative bacteria
MPH ASIOC01	Region 2	NRP-metallophore	3,389,865	3,439,730	Enterobactin (NRP)	60%	49,865	The strongest siderophore known, binding to the ferric ion (Fe^{3+})
MPH MP23	Region 49.1	NRP-metallophore	75,890	125,755	Enterobactin (NRP)	60%	49,865	The strongest siderophore known, binding to the ferric ion (Fe^{3+})
MYI	Region 2.1	NRP-metallophore	300,463	350,324	Enterobactin (NRP)	60%	49,861	The strongest siderophore known, binding to the ferric ion (Fe^{3+})
MPL	Region 1.1	NRP-metallophore	336,102	385,964	Enterobactin (NRP)	60%	49,862	The strongest siderophore known, binding to the ferric ion (Fe^{3+})
MFB070	Region 2.1	NRP-metallophore	111,826	161,688	Enterobactin (NRP)	60%	49,862	The strongest siderophore known, binding to the ferric ion (Fe^{3+})
MPH ASIOC01	Region 3	Arylpolyene	4,462,440	4,506,036	Aryl polyenes	100%	43,596	Bacteria pigment, anti-oxidative properties are similar to those of carotenoids
MPH MP23	Region 5.1	Arylpolyene	17,610	61,206	Aryl polyenes	100%	43,596	Bacteria pigment, anti-oxidative properties are similar to those of carotenoids
MYI	Region 1.1	Arylpolyene	66,864	110,460	Aryl polyenes	100%	43,596	Bacteria pigment, anti-oxidative properties are similar to those of carotenoids
MPL	Region 15.1	Arylpolyene	18,612	62,208	Aryl polyenes	100%	43,596	Bacteria pigment, anti-oxidative properties are similar to those of carotenoids
MFB070	Region 10.1	Arylpolyene	23,183	66,779	Aryl polyenes	100%	43,596	Bacteria pigment, anti-oxidative properties are similar to those of carotenoids
MPH ASIOC01	Region 4	RiPP-like	5,697,114	5,707,737	N/A	0%	10,623	Ribosomally synthesized and post-translationally modified peptides
MPH MP23	Region 4.1	RiPP-like	124,602	133,372	N/A	0%	8,770	Ribosomally synthesized and post-translationally modified peptides
MYI	Region 9.1	RiPP-like	122,023	130,478	N/A	0%	8,455	Ribosomally synthesized and post-translationally modified peptides
MPL	Region 13.1	RiPP-like	137,407	146,025	N/A	0%	8,618	Ribosomally synthesized and post-translationally modified peptides
MFB070	Region 47.1	RiPP-like	1	8,518	N/A	0%	8,517	Ribosomally synthesized and post-translationally modified peptides

Supplementary Tables

TABLE S6

Genes associated with antimicrobial resistance for MPH MP23^T, MPH ASIOC01, MYI SaN21-3, MPL MSSRF40^T, and MGB sp. MFB070. These strains have exactly a similar set of genes associated with antimicrobial resistance. The outcomes were generated using PATRIC v3.6.12 (Comprehensive Genome Analysis).

AMR Mechanism	Genes
Antibiotic activation enzyme	*KatG*
Antibiotic resistance gene cluster, cassette, or operon	*MarA, MarR*
Antibiotic target in susceptible species	*Alr, Ddl, dxr, EF-G, EF-Tu, folA, Dfr, folP, gyrA, gyrB, inhA, fabI, Iso-tRNA, kasA, MurA, rho, rpoB, rpoC, S10p, S12p*
Antibiotic target protection protein	*BcrC*
Antibiotic target replacement protein	*fabV*
Efflux pump conferring antibiotic resistance	*AcrAB-TolC, AcrAD-TolC, AcrZ, EmrAB-OMF, EmrAB-TolC, MacA, MacB, MdfA/Cmr, MdtABC-OMF, MdtABC-TolC, TolC/OpmH*
Gene conferring resistance via absence	*gidB*
Protein altering cell wall charge conferring antibiotic resistance	*GdpD, PgsA*
Protein modulating permeability to antibiotic	*OccD6/OprQ, OprB*
Regulator modulating expression of antibiotic resistance genes	*AcrAB-TolC, EmrAB-TolC, H-NS, OxyR*

<u>Supplementary Tables</u>

TABLE S7

The counts of specialized genes are related to MGBs, and the databases were employed in this investigation.

Features	Source	*M. phragmitis*	*M. phragmitis*	*M. plantisponsor*	*M. yixingensis*	*Mangrovibacter* sp.	Average
GenBank assembly accession		This study	GCA_001655675.1	GCA_003182475.1	GCA_020523985.1	GCA_000705335.1	N/A
Strain ID		ASIOC01	MP23^T	MSSRF40^T	SaN21-3	MFB070	N/A
Antibiotic Resistance	CARD	32	33	33	36	32	33
Antibiotic Resistance	PATRIC	53	56	64	57	60	58
Drug target	DrugBank	209	208	208	213	207	209
Drug target	TTD	42	44	42	44	42	43
Transporter	TCDB	328	315	336	334	330	329
Virulence Factor	PATRIC_VF	83	78	78	76	77	78
Virulence Factor	VFDB	12	11	11	11	11	11
Virulence Factor	Victors	111	107	106	106	104	107
Prediction tool		PATRIC v3.6.12	PATRIC v3.6.12	PATRIC v3.6.12	PATRIC v3.6.12	PATRIC v3.6.12	N/A

N/A: Not applicable

Supplementary Tables

TABLE S8

Locus tags corresponding to genes of interest specified in Table 3.

<table>
<tr><th></th><th></th><th colspan="5">Locus tag* (RefSeq ID or BRC ID[#])</th></tr>
<tr><th>Phenotype</th><th>Gene ID</th><th>MPH ASIOC01 (SAMN37132003)</th><th>MPH MP23 (BioSample ID: SAMN05177220)</th><th>MPL (BioSample ID: SAMN09064728)</th><th>MYI (BioSample ID: SAMN21557958)</th><th>MFB070 (BioSample ID: SAMN02719562)</th></tr>
<tr><td>Cellulose-degradation</td><td>bcsZ</td><td>MPHASIOC01_000290</td><td>A9B99_07335</td><td>DES37_1102</td><td>LC084_RS17965</td><td>DT73_09180</td></tr>
<tr><td>Cellulose-degradation</td><td>NA</td><td>MPHASIOC01_000013</td><td>A9B99_05970</td><td>DES37_11630</td><td>LC084_RS18590</td><td>DT73_14835, fig|1224318.5.peg.4324[#]</td></tr>
<tr><td>Cellulose-degradation</td><td>bglX</td><td>MPHASIOC01_002426</td><td>A9B99_04435</td><td>DES37_10226</td><td>LC084_RS10415</td><td>DT73_22310</td></tr>
<tr><td>Cellulose-degradation</td><td>NA</td><td>MPHASIOC01_000282, 000135</td><td>A9B99_07290, 06560</td><td>DES37_11010, 110163</td><td>fig|1529639.12.peg.3844[#], fig|1529639.12.peg.4112[#]</td><td>DT73_09135, 26120</td></tr>
<tr><td>Cellulose biosynthesis</td><td>bcsA</td><td>MPHASIOC01_000141, 000278, 000288</td><td>A9B99_06590, 07325, 07270</td><td>DES37_11014, 110157, 1104</td><td>LC084_RS18025, 17975, 19250</td><td>DT73_09170, fig|1224318.5.peg.5408[#], fig|1224318.5.peg.1882[#]</td></tr>
<tr><td>Cellulose biosynthesis</td><td>bcsB</td><td>MPHASIOC01_000279, 000289</td><td>A9B99_07330, 07275</td><td>DES37_11013, 1103</td><td>LC084_RS18020, 17970</td><td>fig|1224318.5.peg.1883[#], DT73_09175</td></tr>
<tr><td>Cellulose biosynthesis</td><td>bcsC</td><td>MPHASIOC01_000291</td><td>A9B99_07340</td><td>DES37_1101</td><td>LC084_RS17960</td><td>DT73_09185</td></tr>
<tr><td>Cellulose biosynthesis</td><td>bcsD</td><td>MPHASIOC01_000138, 000281</td><td>A9B99_07285, 06575</td><td>DES37_11011, 110160</td><td>fig|1529639.12.peg.3845[#], fig|1529639.12.peg.4115[#]</td><td>DT73_09130, 26135</td></tr>
<tr><td>Cellulose biosynthesis</td><td>bcsE</td><td>MPHASIOC01_000285</td><td>A9B99_07310</td><td>DES37_1107</td><td>LC084_RS17990</td><td>DT73_09155</td></tr>
<tr><td>Cellulose biosynthesis</td><td>bcsF</td><td>MPHASIOC01_000284</td><td>A9B99_07305</td><td>DES37_1108</td><td>LC084_RS17995</td><td>DT73_09150</td></tr>
<tr><td>Cellulose biosynthesis</td><td>bcsG</td><td>MPHASIOC01_000283</td><td>A9B99_07300</td><td>DES37_1109</td><td>LC084_RS18000</td><td>DT73_09145</td></tr>
<tr><td>Cellulose biosynthesis</td><td>bcsO</td><td>MPHASIOC01_000276</td><td>A9B99_07260</td><td>DES37_11016</td><td>LC084_RS18035</td><td>DT73_09110</td></tr>
<tr><td>Cellulose biosynthesis</td><td>bcsQ</td><td>MPHASIOC01_000277, 000287</td><td>A9B99_07320, 07265</td><td>DES37_1105, 11015</td><td>fig|1529639.12.peg.3837[#], fig|1529639.12.peg.3849[#]</td><td>DT73_09165, 09115</td></tr>
<tr><td>Cellulose biosynthesis</td><td>dgcQ</td><td>MPHASIOC01_001652</td><td>A9B99_02725</td><td>DES37_108222</td><td>fig|1529639.12.peg.1843[#]</td><td>DT73_00160</td></tr>
<tr><td>Nitrogen-fixation</td><td>nifA</td><td>MPHASIOC01_000881</td><td>A9B99_17330</td><td>DES37_10425</td><td>LC084_RS05000</td><td>DT73_11540</td></tr>
<tr><td>Nitrogen-fixation</td><td>nifB</td><td>MPHASIOC01_000882</td><td>A9B99_17325</td><td>DES37_10424</td><td>LC084_RS05005</td><td>DT73_11545</td></tr>
<tr><td>Nitrogen-fixation</td><td>nifD</td><td>MPHASIOC01_000866</td><td>A9B99_17405</td><td>DES37_10440</td><td>LC084_RS04925</td><td>DT73_11465</td></tr>
<tr><td>Nitrogen-fixation</td><td>nifE</td><td>MPHASIOC01_000870</td><td>A9B99_17385</td><td>DES37_10436</td><td>LC084_RS04945</td><td>DT73_11485</td></tr>
<tr><td>Nitrogen-fixation</td><td>nifH</td><td>MPHASIOC01_000865</td><td>A9B99_17410</td><td>DES37_10441</td><td>LC084_RS04920</td><td>DT73_11460</td></tr>
<tr><td>Nitrogen-fixation</td><td>nifJ</td><td>MPHASIOC01_000864, 001342</td><td>A9B99_01105, 17415</td><td>DES37_10442, 103151</td><td>LC084_RS07020, 04915</td><td>DT73_11455, 19275</td></tr>
<tr><td>Nitrogen-fixation</td><td>nifK</td><td>MPHASIOC01_000867</td><td>A9B99_17400</td><td>DES37_10439</td><td>LC084_RS04930</td><td>DT73_11470</td></tr>
<tr><td>Nitrogen-fixation</td><td>nifL</td><td>MPHASIOC01_000880</td><td>A9B99_17335</td><td>DES37_10426</td><td>fig|1529639.12.peg.1067[#]</td><td>DT73_11535</td></tr>
<tr><td>Nitrogen-fixation</td><td>nifM</td><td>MPHASIOC01_000878</td><td>A9B99_17345</td><td>DES37_10428</td><td>LC084_RS04985</td><td>DT73_11525</td></tr>
<tr><td>Nitrogen-fixation</td><td>nifN</td><td>MPHASIOC01_000871</td><td>A9B99_17380</td><td>DES37_10435</td><td>LC084_RS04950</td><td>DT73_11490</td></tr>
<tr><td>Nitrogen-fixation</td><td>nifS</td><td>MPHASIOC01_000874</td><td>A9B99_17365</td><td>DES37_10432</td><td>LC084_RS04965</td><td>DT73_11505</td></tr>
<tr><td>Nitrogen-fixation</td><td>nifU</td><td>MPHASIOC01_000873</td><td>A9B99_17370</td><td>DES37_10433</td><td>LC084_RS04960</td><td>DT73_11500</td></tr>
<tr><td>Nitrogen-fixation</td><td>nifV</td><td>MPHASIOC01_000875</td><td>A9B99_17360</td><td>DES37_10431</td><td>LC084_RS04970</td><td>DT73_11510</td></tr>
<tr><td>Inorganic phosphate-solubilizing</td><td>ppx</td><td>MPHASIOC01_002786</td><td>A9B99_18825</td><td>DES37_10577</td><td>LC084_RS16715</td><td>DT73_25265</td></tr>
<tr><td>Inorganic phosphate-solubilizing</td><td>ppa</td><td>MPHASIOC01_004901</td><td>A9B99_08645</td><td>DES37_12343</td><td>LC084_RS22575</td><td>DT73_16785</td></tr>
<tr><td>Inorganic phosphate-solubilizing</td><td>gcd</td><td>MPHASIOC01_004482</td><td>A9B99_10280</td><td>DES37_10669</td><td>fig|1529639.12.peg.3053[#]</td><td>DT73_07290</td></tr>
<tr><td>Organic phosphorus mineralizing</td><td>phoA</td><td>MPHASIOC01_004203</td><td>A9B99_11030</td><td>DES37_1217</td><td>fig|1529639.12.peg.288[#]</td><td>DT73_15170</td></tr>
<tr><td>Phosphate transporter</td><td>pitA</td><td>MPHASIOC01_000317</td><td>A9B99_07490</td><td>DES37_11766</td><td>LC084_RS17830</td><td>DT73_21540</td></tr>
<tr><td>Phosphate transporter</td><td>pstA</td><td>MPHASIOC01_005489, 005494</td><td>A9B99_05860</td><td>DES37_11654</td><td>LC084_RS18465</td><td>DT73_20990</td></tr>
<tr><td>Phosphate transporter</td><td>pstB</td><td>MPHASIOC01_005490, 005495</td><td>A9B99_05865</td><td>DES37_11653</td><td>LC084_RS18470</td><td>DT73_20985</td></tr>
<tr><td>Phosphate transporter</td><td>ugpQ</td><td>MPHASIOC01_000377</td><td>A9B99_07790</td><td>DES37_1178</td><td>LC084_RS17500</td><td>DT73_16610</td></tr>
<tr><td>Phosphate intake regulatory</td><td>phoB</td><td>MPHASIOC01_004193</td><td>A9B99_11080</td><td>DES37_107324</td><td>LC084_RS01395</td><td>DT73_15115</td></tr>
<tr><td>Phosphate intake regulatory</td><td>phoR</td><td>MPHASIOC01_004192</td><td>A9B99_11085</td><td>DES37_107323</td><td>LC084_RS01400</td><td>DT73_15110</td></tr>
<tr><td>PolyP synthesis</td><td>ppk1</td><td>MPHASIOC01_002785</td><td>A9B99_18820</td><td>DES37_10578</td><td>LC084_RS16710</td><td>DT73_25260</td></tr>
<tr><td>Cr reduction</td><td>nfsA</td><td>MPHASIOC01_000547</td><td>A9B99_12925</td><td>DES37_104340</td><td>LC084_RS03630</td><td>DT73_10050</td></tr>
<tr><td>Cr reduction</td><td>nfsB</td><td>MPHASIOC01_001267</td><td>A9B99_00775</td><td>DES37_103216</td><td>LC084_RS06690</td><td>DT73_19615</td></tr>
<tr><td>Cr reduction</td><td>nemA</td><td>MPHASIOC01_001090</td><td>A9B99_18035</td><td>DES37_11232</td><td>fig|1529639.12.peg.1220[#]</td><td>DT73_12375</td></tr>
<tr><td>Dye degradation</td><td>AzrG</td><td>MPHASIOC01_001328</td><td>A9B99_01070</td><td>DES37_103158</td><td>fig|1529639.12.peg.1489[#]</td><td>DT73_19310</td></tr>
</table>

*Souce of MGB's genome were listed in Table S1A.

[#]BRC IDs were generated by BV-BRC information system using BLASTP feature (protein > protein database) when no corresponding RefSeq ID was found.
